# Novel enzymes for amino acid biosynthesis in diverse bacteria and archaea: GapMind 2026

**DOI:** 10.1371/journal.pgen.1012372

**Published:** 2026-11-12

**Authors:** Morgan N. Price, Valentine V. Trotter, Markus de Raad, Suzanne M. Kosina, Hira P. Lesea, Anthony L. Shiver, Leslie A. Day, Surya Tripathi, Marta Torres, Trenton K. Owens, Audrey Liwen Wang, Anna Lisa Fear, Xuanyu Tao, Jizhong Zhou, Adam M. Deutschbauer, Adam P. Arkin

**Affiliations:** 1 Environmental Genomics and Systems Biology, Lawrence Berkeley National Lab, Berkeley, California, United States of America; 2 Department of Bioengineering, Stanford University, Stanford, California, United States of America; 3 Geisel School of Medicine, Dartmouth College, Hanover, New Hampshire, United States of America; 4 Institute for Environmental Genomics, School of Biological Sciences, University of Oklahoma, Norman, Oklahoma, United States of America; Indiana University Bloomington, UNITED STATES OF AMERICA

## Abstract

We previously described GapMind, an automated web-based tool for annotating amino acid biosynthesis pathways in bacterial and archaeal genomes. We used GapMind to identify gaps in biosynthetic pathways and systematically used comparative genomics and high-throughput genetics to identify candidate genes to fill these gaps. We confirmed the activity of ten of the proposed enzymes by using cross-species complementation assays. Highlights include a route to glycine via glyoxylate, two families that can replace phosphoserine phosphatase, an alternative N-succinyl-L,L-diaminopimelate desuccinylase, an alternative N-acetylornithine deacetylase, and a putative bifunctional MetB/MetC. We updated GapMind to include these additional enzymes. Across 208 prokaryotes that have high-quality genomes and can grow in minimal media, the average number of unexplained missing steps or gaps in amino acid biosynthesis dropped from 1.4 per genome to 0.7 per genome. The majority of remaining gaps involve the gain or loss of phosphate groups.

## Author summary

All living organisms use a standard set of 20 amino acids to build their proteins, and most bacteria and archaea can synthesize all of these amino acids. For the best-studied bacteria, we understand all of the enzymatic reactions for their biosynthesis as well as the genes that encode them. But for most bacteria and archaea that can make all 20 amino acids, there are apparent gaps in the biosynthetic pathways, which implies that novel enzymes remain to be discovered. We studied these gaps with large-scale genetic experiments and by comparing the genomes of these microbes. We predicted many new biosynthetic enzymes, and we confirmed some of these predictions with genetic experiments. We then incorporated the new enzymes into a tool for annotating biosynthetic pathways. For diverse bacteria and archaea that can make all 20 amino acids, the average number of gaps dropped from 1.4 to 0.7. This will help scientists understand the nutritional requirements of diverse microbes.

## Introduction

Knowing the growth requirements of microorganisms is important for understanding their ecological roles [1]. Most free-living bacteria can probably make all of the standard amino acids [1], but in many of their genomes, the genes for some biosynthetic enzymes cannot be identified. These gaps occur even in the genomes of bacteria and archaea that are experimentally confirmed to be prototrophic [2]. These gaps make it challenging to predict the growth requirements of an organism from its genome sequence, except for well-studied groups such as Enterobacteria or *Pseudomonas* [3]. For other bacteria, predictions of amino acid auxotrophies are often incorrect [4, 5]. These gaps in biosynthetic pathways also imply that many novel biosynthetic enzymes remain to be discovered.

We previously described GapMind, a fast web-based tool for annotating amino acid biosynthesis pathways in bacteria and archaea [2]. Because many of the biosynthetic enzymes are not described in standard databases such as Swiss-Prot, MetaCyc, or BRENDA [6–8], we curated lists of experimentally-characterized enzymes that can carry out each biosynthetic step. We also included novel enzymes that we identified using randomly-barcoded transposon sequencing (RB-TnSeq) [2, 4]. Given a genome of interest, GapMind uses these characterized enzymes, along with curated families (hidden Markov models) from TIGRFams [9], to identify candidates for each step, at varying levels of confidence (Fig 1A). GapMind also uses similarity to proteins with computationally-predicted functions (from Swiss-Prot) to identify additional candidates, but these are never considered high-confidence candidates. Most often, a high-confidence candidate is at least 40% identical to a characterized enzyme, and is less similar to characterized proteins with other functions. GapMind then reports the highest-confidence candidates for each step. Finally, if there are alternate pathways, GapMind selects the pathway that has the most high-confidence candidates and the fewest gaps (steps with only low-confidence candidates or no candidates). An example of GapMind’s results is shown in Fig 1B.

**Fig 1:**
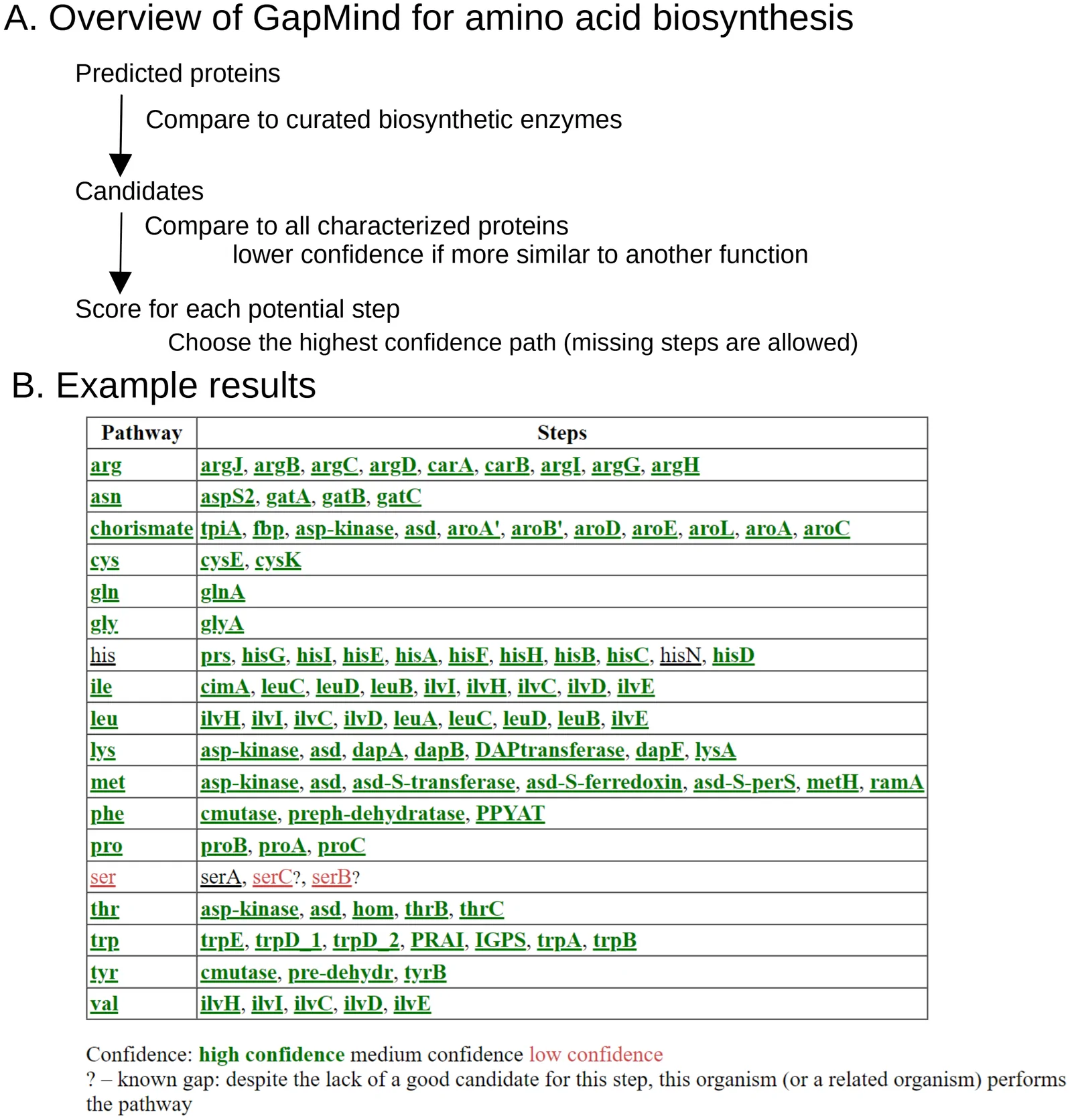
Overview of GapMind. The example results are for *Nitratidesulfovibrio vulgaris* Hildenborough. In this screenshot, steps and pathways are color-coded by confidence level. All steps are high-confidence except for histidinol-phosphate phosphatase (*hisN*) and the three steps of serine synthesis. Despite these potential gaps, *N. vulgaris* grows in a defined minimal medium [10] and can synthesize all of the standard amino acids.

Since the original publication of GapMind in 2020, over six hundred relevant proteins have been curated in MetaCyc, BRENDA, Swiss-Prot, or the Fitness Browser reannotations [6–8, 11]. Also, novel enzymes involved in amino acid biosynthesis were predicted based on comparative genomics [12, 13], and a novel pathway for methionine synthesis was identified in *Streptomyces* [14]. Furthermore, we have collected RB-TnSeq data from many more organisms, including additional phyla of bacteria and archaea [15, 16]. As we will show, this allowed us to identify 17 novel or diverged biosynthetic genes. Finally, we used cross-species complementation assays to confirm the functions of 10 other genes. Together, we provide experimental evidence for novel or diverged enzymes involved in the biosynthesis of arginine, chorismate (the precursor to the aromatic amino acids), glycine, isoleucine, leucine, lysine, methionine, serine, and threonine. Based on this new knowledge, we updated GapMind and improved its ability to explain amino acid biosynthesis in diverse bacteria and archaea. GapMind is available at http://papers.genomics.lbl.gov/gaps.

## Results and discussion

We systematically tried to fill gaps in amino acid biosynthesis pathways across diverse prototrophic prokaryotes, using two main approaches. Our first approach relies on large-scale genetic data from RB-TnSeq. The biosynthetic genes for an amino acid should be important for fitness in defined media unless the amino acid is added. Alternatively, the biosynthetic genes should have similar fitness patterns with each other (“cofitness”). This approach can usually fill the gap, but it is limited to organisms that have RB-TnSeq libraries. Our second approach is comparative genomics: this mostly relies on the fact that biosynthetic pathways are often organized into operons. So, given a gap in an organism that is known to grow in a minimal medium, we can search for genes that are in conserved operons together with other parts of the biosynthetic pathway, and that could plausibly replace the missing gene. Either way, we can test the proposed function of the novel gene by expressing it in an auxotrophic mutant of *Escherichia coli*. If the gene allows an otherwise auxotrophic strain to grow in minimal medium, this suggests that it provides the missing biochemical function.

We will first describe the novel enzymes that we identified (listed in Table 1). We consider these putative enzymes to be novel because we do not think their roles could have been predicted by their similarity to known biosynthetic proteins. For all but one of the putative novel enzymes, we have genetic data to support their roles in amino acid biosynthesis, but we do not have biochemical evidence. Second, we will discuss how we incorporated previously-published predictions from comparative genomics into GapMind [12, 13]. Third, we will describe how we improved GapMind’s coverage by annotating diverged enzymes that are under 40% identical to a characterized protein; these would otherwise be identified as lower-confidence candidates or might be missed entirely. Finally, we will assess how well the revised GapMind covers amino acid biosynthesis in prototrophic bacteria and archaea, and, for auxotrophic bacteria, whether GapMind incorrectly identifies complete biosynthetic pathways.

**Table 1:** Putative novel enzymes for amino acid biosynthesis. The experimental evidence for the function of these genes is genetic; biochemical studies will be needed to prove their molecular functions.

Pathway	Name	Description	Evidence	Genus	UniProt
gly	GlyXL	Glyoxylate synthesis, catalytic component	yes	*Bifidobacterium*	Q8G510
gly	GlyXL	‘’	yes	*Methanococcus*	Q6LXC5
gly	GlyXS	Glyoxylate synthesis, regulatory component	yes	*Bifidobacterium*	Q8G509
gly	GlyXS	‘’	yes	*Methanococcus*	Q6LZH1
gly	Agx1	Glyoxylate aminotransferase	yes	*Bifidobacterium*	Q2VPW8
ser	SerBK	Alternative to phosphoserine phosphatase	yes	*Clostridium*_*F*_	A5I0W9
ser	SerB	IMPase-like phosphoserine phosphatase	yes	*Rhodanobacter*	A0A522SXD8
lys	DapE	Alternative N-succinyl-L,L- diaminopimelate desuccinylase	yes	*Echinicola*	L0FXC2
lys	DapE	‘’	yes	*Pedobacter*	A0A0T5VTP7
met	MetBX	Bifunctional cystathionine gamma-synthase and beta-lyase	yes	*Rhodanobacter*	I4WT24
met	MetBX	‘’	yes	*Xanthomonas*	Q5H1U9
arg	ArgE	Alternative N-acetylornithine hydrolase	yes	*Veillonella*	W3Y6L2
arg	ArgA	Alternative N-succinyl- L-glutamate synthase	no	*Steroidobacter*	A0A127FCT3

### Novel genes for glycine synthesis via glyoxylate

The standard pathway for glycine biosynthesis in bacteria and archaea involves a single enzyme, serine hydroxymethyltransferase (GlyA), which converts serine plus tetrahydrofolate (THF) to glycine plus 5,10-methylene-THF. (For detailed pathway renderings that include compound structures, we recommend using EcoCyc: http://ecocyc.org [17].) *Bifidobacterium breve*, a human gut bacterium, and *Methanococcus maripaludis*, a methanogen, are capable of glycine synthesis despite lacking any apparent *glyA* gene. We examined previously-published RB-TnSeq data from both organisms grown in defined media that lack glycine [15, 16]. In both organisms, these experiments highlighted two genes which are important for fitness whenever glycine is not available (Fig 2A & 2B). We will call them *glyXL* and *glyXS*. (GlyXL is MMP_RS07345 or BBR_RS12920, which are 61% amino acid identical; GlyXS is MMP_RS03450 or BBR_RS12915, which are 46% identical.)

**Fig 2:**
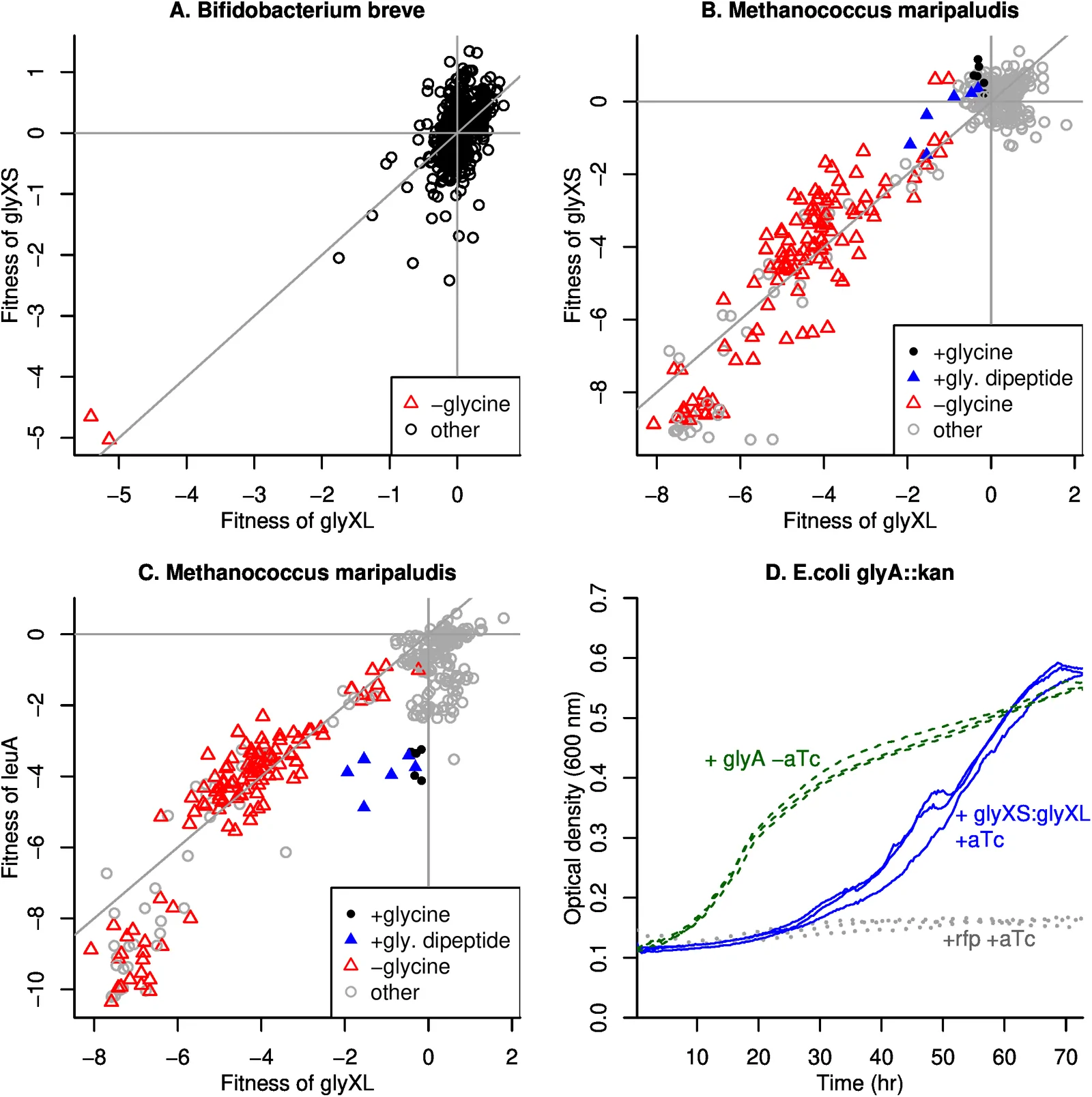
GlyXL and GlyXS are involved in glycine biosynthesis in *Bifidobacterium* and *Methanococcus.* **(A,B)** We show RB-TnSeq data from *B. breve* UCC2003 [16] and *M. maripaludis* S2 [15]. Each point shows the fitness values of *glyXL* and *glyXS* from a different experiment, where a gene’s fitness is the estimated log2 change in the relative abundance of mutants of that gene during growth [18]. Experiments using a defined medium that lacks glycine are highlighted (red triangles). For *M. maripaludis*, data from defined media with glycine, or with glycine-containing dipeptides, are also highlighted. **(C)** Fitness of *glyXL* (*x* axis) and *leuA* (MMP_RS05500, *y* axis) from *M. maripaludis*, with the same color coding as in panel **B.** In each panel, the lines show *x* = 0, *y* = 0, and *x* = *y*. **(D)** G*lyA::kan* strains of *E. coli,* carrying various plasmids, were grown in minimal M9 medium with glucose. The *glyA* plasmid carries the gene from *E. coli*; the *glyXS-glyXL* plasmid carries the genes from *B. breve* with the naturally-occurring spacer between them; and the control plasmid carries *rfp* (red fluorescent protein). Expression of all of these is driven by a pTet promoter. For strains that express *glyXS-glyXL* or *rfp,* 4 nM of the inducer anhydrotetracycline (aTc) was added. For each strain, all three technical replicates are shown.

As shown in Fig 2B, in *M. maripaludis*, *glyXL* and *glyXS* are important for fitness in most of the experiments without added glycine, but there are a few exceptions. These exceptions occur because there was relatively little growth in these conditions, and hence relatively little decrease in the relative abundance of auxotrophic mutants. This can be seen by comparing the fitness pattern of *glyXL* to another amino acid biosynthesis gene, *leuA* (Fig 2C). The two patterns are very similar, except that mutants of *glyXL* are rescued by added glycine or (to a lesser extent) by dipeptides that contain glycine.

GlyXL is distantly related to anaerobic ribonucleotide reductases, which use a glycyl radical and two nearby cysteine residues for catalysis. When we aligned the predicted structure of GlyXL from *B. breve* (AlphaFold version 2.0 for UniProt Q8G510; (Varadi et al. 2022)) to the experimental structure of an anaerobic ribonucleotide reductase (PDB:4COM) using the RCSB web site [19], the structures were not that similar (root mean square deviation 4.38 Å, TM score 0.65), and these catalytic residues were not conserved. So GlyXL may use a different mechanism. GlyXS is an ACT domain protein (PFam PF13740; [20]); members of this family often bind amino acids to regulate metabolism.

A role for the GlyXL family in glycine synthesis was previously reported in *Streptococcus pneumoniae* [21]. Specifically, *S. pneumoniae* D39 is auxotrophic for glycine and has a truncation in *glyXL* (*spr0218*), and glycine prototrophy can be restored by replacing *glyXL* with the full-length version from a prototrophic strain [21]. A complication in interpreting these data is that *S. pneumoniae* D39 also encodes *glyA*. However, metabolite labeling patterns imply that strain D39 uses GlyA in reverse, to convert glycine to serine [22]. Furthermore, although strain D39 seems to lack the standard pathway for forming serine (no 3-phosphoglycerate dehydrogenase is apparent in the genome), it does not require serine for growth [21, 22]; this is consistent with the conversion of glycine to serine.

Across diverse bacteria and archaea, GlyXL and GlyXS usually co-occur and are usually in a putative operon. For example, in the *fast.genomics* database of representative genomes [23], potential orthologs of GlyXL and GlyXS (above 30% of the best possible bit score) are found in 978 genera, out of 1,131 genera that contain either gene (86%). Among the 978 genera, *glyXL* and *glyXS* are encoded within 5 kb and on the same strand in 815 genera (83%). (But they are not encoded nearby in *M. maripaludis.*) Furthermore, in *Bacillus methanolicus*, expression of the *glyXS-glyXL* operon appears to be regulated by a glycine riboswitch (see BMMGA3_03000 in [24]). Although many of the organisms with GlyXL and GlyXS are anaerobic, *B. methanolicus* is obligately aerobic [25], so we predicted that GlyXL-GlyXS would function in the presence of oxygen.

To test if GlyXL-GlyXS can contribute to glycine biosynthesis, we cloned *glyXS* and *glyXL* from *B. breve* into an anhydrotetracycline-inducible expression vector (pBbA2c [26]) and introduced them into a *glyA::kan* strain of *E. coli* [27]. We found that *glyXS-glyXL* enabled growth in minimal M9 medium in the absence of *glyA* (Fig 2D). These experiments were conducted aerobically, which confirms that GlyXL is not a glycyl radical enzyme.

What is the product of GlyXL-GLyXS? Besides these two proteins, fitness data for *B. breve* suggests that glycine synthesis also involves a putative transaminase (BBR_RS14535, fitness = -4.3 or -5.1 in the glycine-free experiments and fitness ≥ -1.1 in the other experiments). The fitness data for *B. breve* did not identify any other candidates for glycine synthesis genes (there were no other genes with fitness under -3 in both no-glycine experiments and above -2 in all other 84 experiments with a defined medium.) So, we predict that BBR_RS14535 is a glyoxylate transaminase (forming glycine) and that GlyXL-GlyXS produce glyoxylate rather than glycine. Although the gene encoding glyoxylate transaminase in *E. coli* is not known, *E. coli* does have an active glyoxylate transaminase [28], so this proposal is consistent with the complementation data. Also, the cofolding prediction tool Boltz-2x [29] identified a positively-charged binding pocket for glyoxylate in GlyXL (see Methods), with three arginines (R22, R75, R403) and one negatively-charged residue (E339). This is consistent with a negatively-charged substrate. (Boltz-2 also predicts that GlyXL binds glyoxylate more tightly than it binds glycine, with affinities of 17 μM versus 822 μM.) Finally, many of the genomes that encode *glyXL* and *glyXS* encode *glyA* as well, but unlike *S. pneumoniae*, most of the genomes with all three of these genes seem to encode the standard pathway for forming serine as well. This is consistent with independent pathways with different starting points for glycine synthesis. In the revised GapMind, glycine can be formed by GlyXL-GlyXS along with a glyoxylate transaminase, and BBR_RS14535 is annotated as a glyoxylate transaminase.

### An alternative to phosphoserine phosphatase from the DUF1015 family

In most prokaryotes, serine is formed from 3-phosphoglycerate, which is an intermediate of glycolysis or gluconeogenesis, in three steps (Fig 3A): 3-phosphoglycerate dehydrogenase (SerA), phosphoserine transaminase (SerC) in reverse, and phosphoserine phosphatase (SerB). No phosphoserine phosphatase (SerB) is apparent in the genome of *Clostridioides difficile* strain 630*Δerm*, but cell extracts of *C. difficile* can convert 3-phosphoglycerate to serine [30]. In *C. difficile*, SerA and SerC are encoded in an operon with a DUF1015 family protein, CDIF630erm_01132 (ARE61905.1), so it was proposed that CDIF630erm_01132 is the missing phosphatase [30].

**Fig 3:**
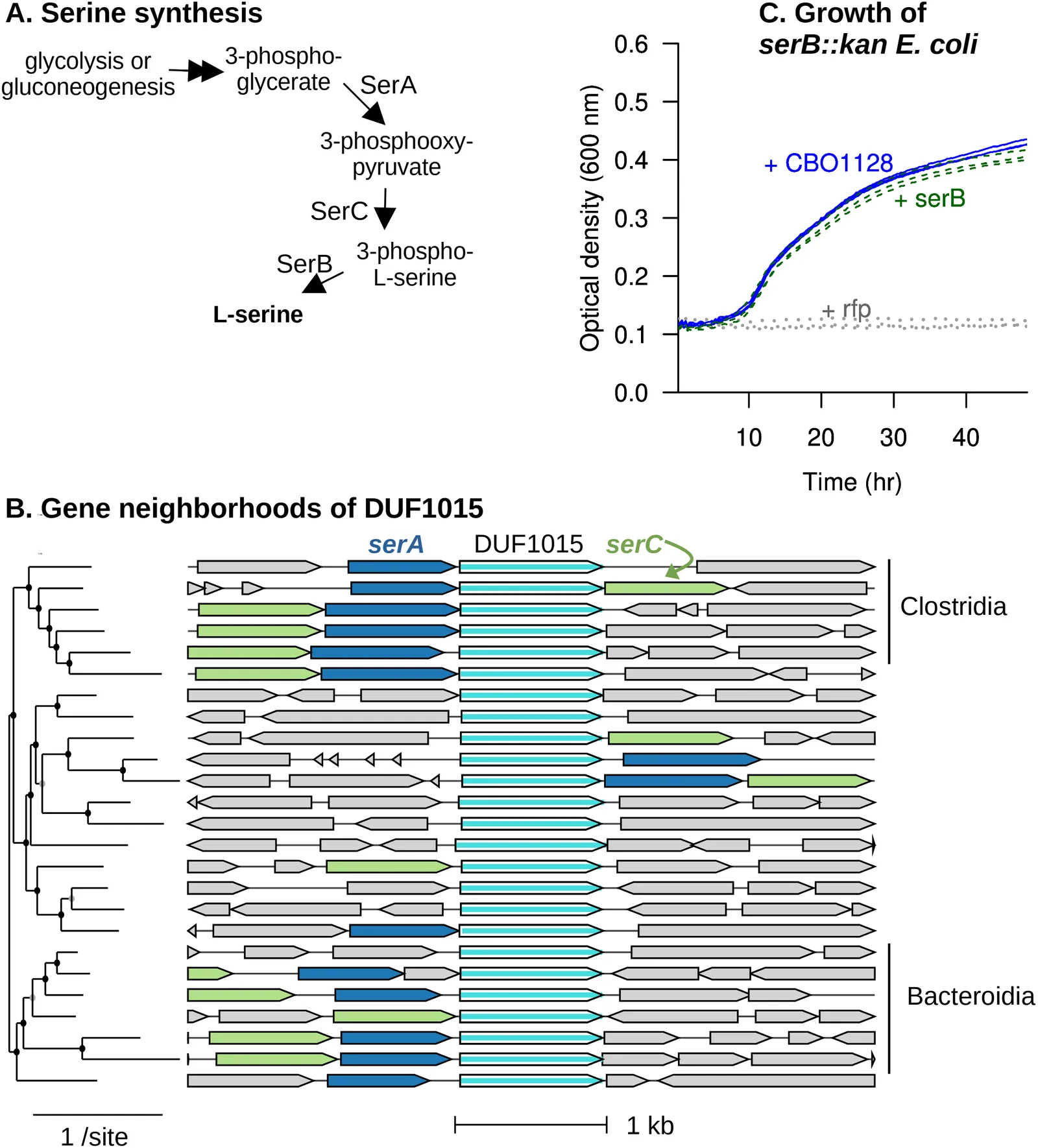
An alternative to phosphoserine phosphatase (SerB) from the DUF1015 family. (**A)** The standard pathway for serine synthesis. **(B)** Each row shows a homolog of the putative alternative enzyme (central column) from a different genus, along with its nearby genes. Genes for *serA* and *serC* are color coded. Homologs from *fast.genomics* [23] were randomly selected from among those whose similarity to CDIF630erm_01132 is at least 30% of the maximum bit score. The phylogenetic tree (from *fast.genomics*) shows the relationships between the DUF1015 proteins. **(C)** Growth of *serB::kan* strains of *E. coli* carrying various plasmids in minimal M9 medium. (No inducer was added.) CBO1128 is a DUF1015 protein, *serB* was taken from *E. coli*, and *rfp* was used as a control. For each strain, all three technical replicates are shown.

Given this hypothesis, we examined the gene neighbors of CDIF630erm_01132 in diverse bacteria. As shown in Fig 3B, homologs of CDIF630erm_01132 are often encoded near *serA*, and are often encoded near a likely *serC* as well. Also, if CDIF630erm_01132 is a replacement for SerB, then its close homologs should be found in genomes that lack the previously-known forms of SerB. We randomly selected 50 representative genomes (all from different genera) that have relatively high-scoring homologs of the putative phosphatase (at least 445 bits, corresponding to roughly 50% identity or above) encoded near *serA*. We ran the updated version of GapMind on these 50 genomes and it identified high-confidence candidates for SerB (at least 40% amino acid identity to a characterized protein) in just three of those 50 genomes. (One of these three was a metagenome-assembled genome, GCA_022072225.1, that also contained two *serA* genes, so it may reflect contamination.) Overall, the vast majority of the genomes that encode close homologs of CDIF630erm_01132 in proximity to *serA* lack *serB*, which again suggests that CDIF630erm_01132 could be a replacement for SerB.

To test this hypothesis experimentally, we cloned the corresponding gene from *Clostridium*_*F*_ *botulinum* (CBO1128, whose amino acid sequence is 50% identical to CDIF630erm_01132) into the aTc-inducible expression vector pBbA2c. We found that CBO1128 allowed a *serB::kan* strain of *E. coli* [27] to grow in minimal medium (Fig 3C).

What is the molecular function of the DUF1015 family? The original study suggested that CDIF630erm_01132 “shows weak homologies to hydrolases” [30], but we did not identify any sequence similarity to characterized proteins. To identify structural homologs, we used the predicted structure (AlphaFold version 2.0 for UniProt A0A6B4WGC7; [31]) as a query in Foldseek [32]. The top hit from Foldseek was the predicted structure of serine kinase SbnI (UniProt Q2G1M5, root mean square deviation 7.2, e-value 2.7· 10^-7^). Although this homology is remote, the structural alignment suggested that the ATP binding and catalytic residues are conserved. Also, when we docked CDIF630erm_01132 with ATP using AlphaFold 3 [33], it made a confident prediction of a binding site (interface predicted template modeling score ipTM = 0.91). Thus, even though this family can replace *serB*, they may be kinases rather than phosphatases.

The human gut bacterium *Bacteroides thetaiotaomicron* VPI-5482 encodes a DUF1015 protein, BT1151, which is 47% identical to CDIF630erm_01132. As discussed in S1A Text, we found that BT1151 was important for the utilization of L-serine as a nitrogen source (gene fitness = -2.2 to -2.6), but it is not important for fitness in most other conditions (data of [34]). It is difficult to see how phosphatase activity would support the utilization of serine. In contrast, serine kinase would form phosphoserine, which could be transformed by SerC to 3-phosphohydroxypyruvate and then by SerA (in reverse) to 3-phosphoglycerate, which is an intermediate in glycolysis. A similar pathway for serine utilization was proposed in *Thermococcus kodakarensis* (but not involving DUF1015; [35]). Also, although *B. thetaiotaomicron* encodes *serB* (BT0832), *serB* is not important for fitness in most defined media conditions (median fitness = -0.2 across 116 experiments, data of [34]). It is also striking that BT1151 is encoded just downstream of *serA* (BT1152) and they are apparently co-transcribed, as no transcript start was identified between BT1151 and BT1152 using Terminator Exonuclease-treated RNA-Seq [36]. The simplest explanation is that the BT1151 can replace *serB* for serine synthesis, despite being a kinase rather than a phosphatase.

We propose that CDIF630erm_01132, CBO1128, BT1151, and related proteins from DUF1015 are serine kinases that can participate in both serine synthesis and serine utilization. We named this family *SerBK*, as it seems to provide the capabilities of both the phosphatase (SerB) and the kinase (SerK). However, it is not clear how the serine kinase reaction in reverse could yield sufficient flux to serine for growth, as this is thermodynamically unfavorable (estimated equilibrium constant of 4 ⋅ 10^-4^ [37],). Furthermore, growing cells usually have a higher concentration of ATP than ADP, which would push the equilibrium further towards phosphoserine. Another uncertainty is that previously-identified serine kinases are either ATP-dependent or ADP-dependent [35, 38]. Biochemical studies will be necessary to clarify the molecular function of SerBK.

### An alternative phosphoserine phosphatase from the inositol monophosphatase family

In various strains of *Rhodanobacter* and in *Dyella japonica* UNC79MFTsu3.2, which belongs to a related genus, the original GapMind did not identify a convincing candidate for *serB*. However, we noticed that these genomes encode two putative histidinol-phosphate phosphatases (*hisN*). Each genome encodes a *hisN* fused to *hisB* (imidazoleglycerol-phosphate dehydratase), which is presumably annotated correctly, as well as another potential histidinol-phosphate phosphatase from the inositol monophosphatase (IMPase) family. The IMPase family proteins are 38–40% identical to the primary histidinol-phosphate phosphatase (Cg0910) of *Corynebacterium glutamicum* [39]. Both *hisBN* and the IMPase family protein are important for fitness in minimal medium (for instance, in *Rhodanobacter denitrificans* FW104-10B01 and in *D. japonica*). If these IMPase family proteins were histidinol-phosphate phosphatases, then they should be genetically redundant with the *hisN* part of *hisBN*. So we inferred that these IMPase-like proteins have another function. In particular, we predicted that these enzymes would be the missing phosphoserine phosphatases.

To test this hypothesis, we collected RB-TnSeq data for *Rhodanobacter sp.* FW510-R12 in minimal glucose medium, with or without added L-serine. As shown in Fig 4, one of the genes whose fitness was most increased by the addition of L-serine was the IMPase-like protein (LRK53_RS05785), and *serA* and *serC* had similar phenotypes. This supports our prediction that these IMPase-like proteins are alternative phosphoserine phosphatases.

**Fig 4:**
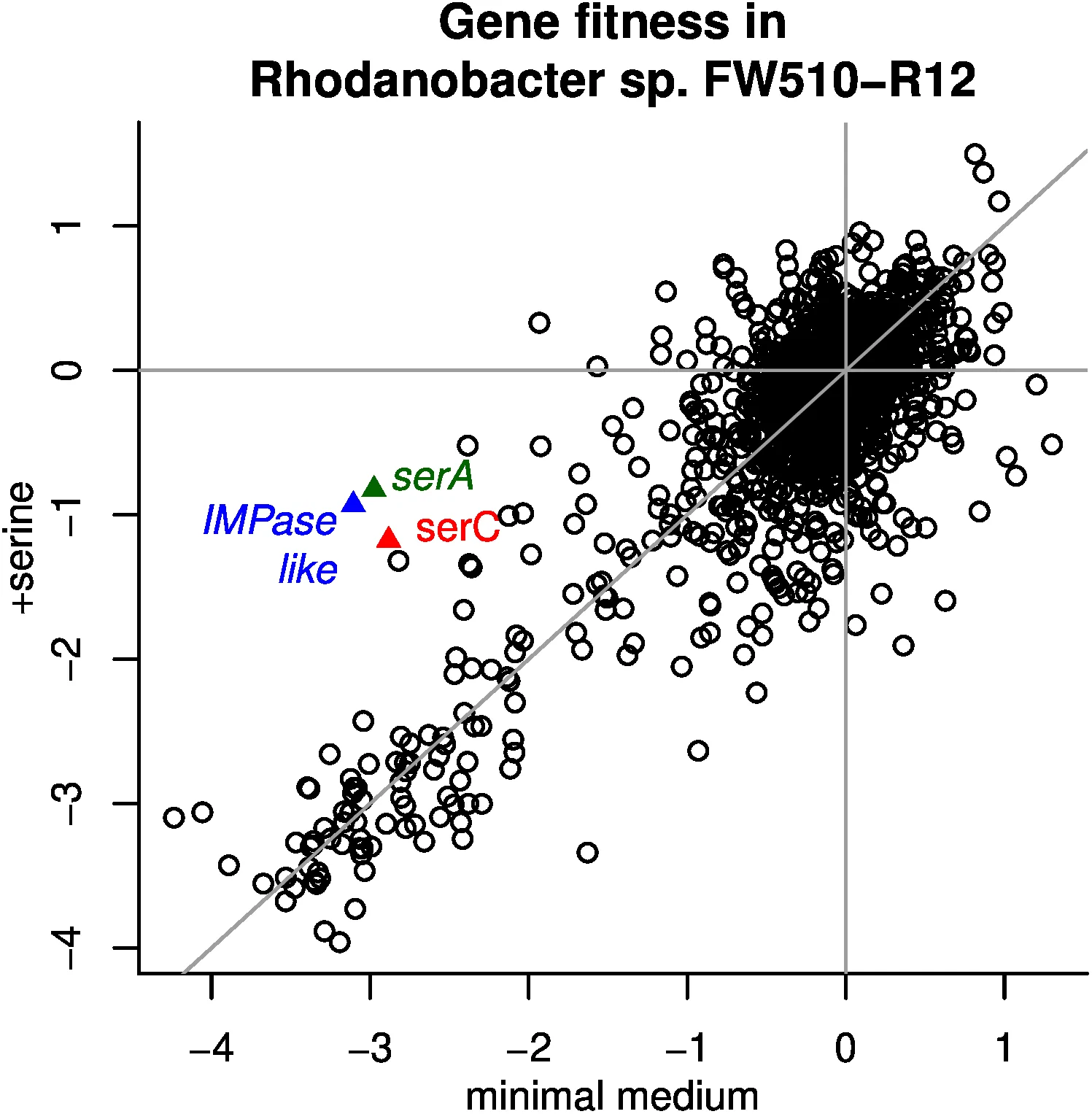
Genetic evidence for an IMPase-like phosphoserine phosphatase in *Rhodanobacter sp.* **FW510-R12.** Each point shows the average fitness of the gene across two replicate RB-TnSeq experiments, either from minimal glucose medium or from minimal glucose medium with 1 mM L-serine. *SerA* (LRK53_RS06795), *serC* (LRK53_RS09750) and *IMPase-like* (LRK53_RS05785) are highlighted.

### An alternative N-succinyl-L,L-diaminopimelate desuccinylase

In most bacteria, the N-succinyl-L,L-diaminopimelate desuccinylase DapE is required for the synthesis of diaminopimelate (DAP), which is a precursor to both lysine and peptidoglycan. DapE is missing in several prototrophic members of the phylum Bacteroidota that encode the tetrahydrodipicolinate succinylase DapD and hence are expected to use the N-succinyl-DAP pathway (*Echinicola vietnamensis* DSM 17526, *Mucilaginibacter yixingensis* YX-36*,* and *Pedobacter sp.* GW460-11-11-14-LB5). Because peptidoglycan synthesis is essential for growth, the gene that replaces *dapE* is expected to be essential. Using RB-TnSeq data for all three of these bacteria [40, 41]; see Methods), we searched for conserved essential genes that did not have known functions, and we identified the putative amidohydrolase Echvi_1427 as a candidate to replace DapE (Fig 5A–5C). The homolog from another genus of Bacteroidota, *Pontibacter actiniarum* KMM 6156*,* is also essential (Fig 5D; data of [40]).

**Fig 5:**
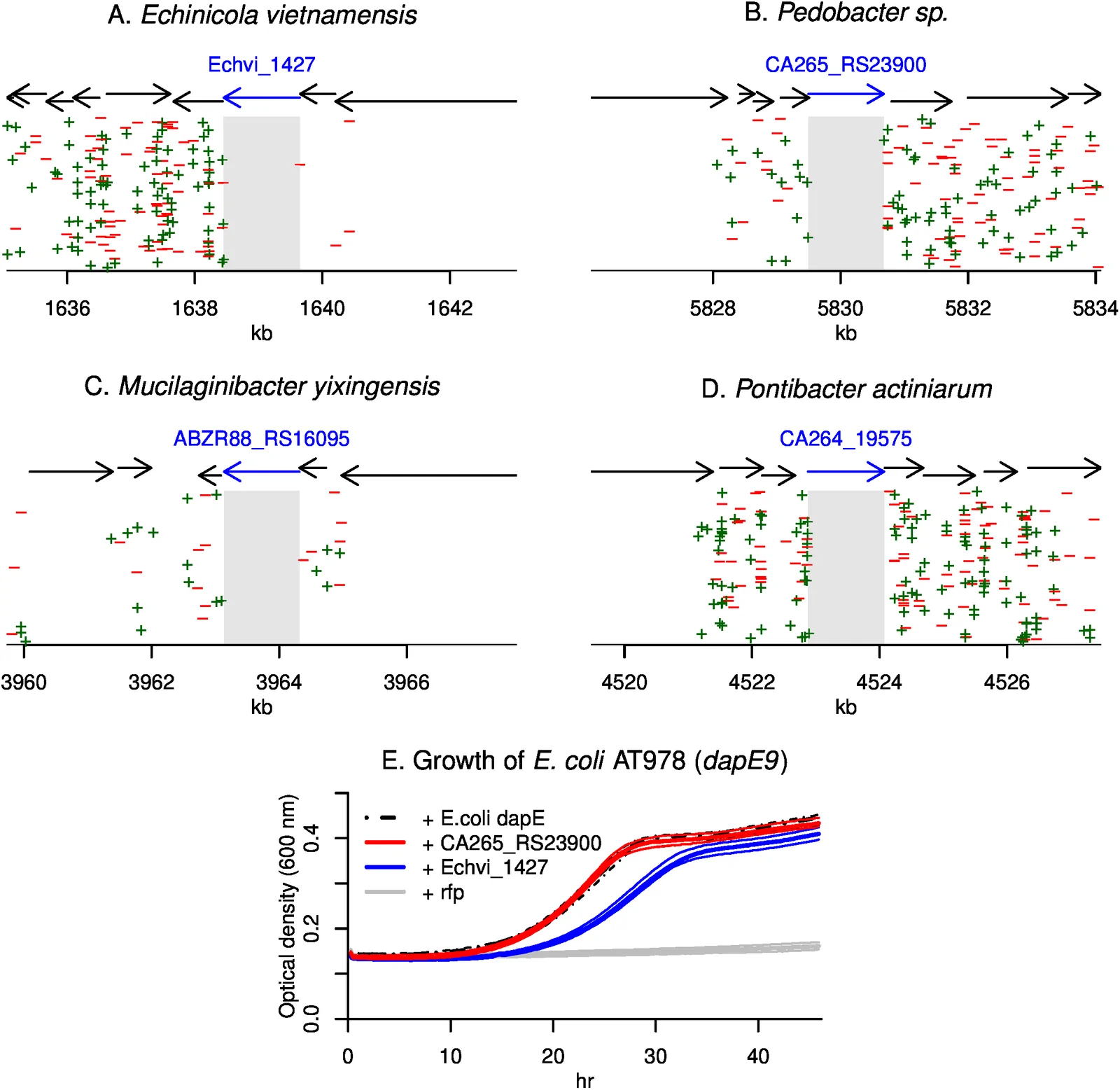
An essential amidohydrolase is an alternative to *dapE.* **(A-D)** The locations of transposon insertions near Echvi_1427 or its homologs. A “+” symbol indicates that the antibiotic resistance marker was inserted on the forward strand. The region covered by the putative amidohydrolase gene is highlighted in grey, and the *y* axis is random. **(E)** Growth of *E. coli* AT978, which carries the *dapE9* allele, in M9 minimal medium when carrying various plasmids. Each thick line is the median of six technical replicates, while thin lines show the minimum and maximum values.

Echvi_1427 is similar to several characterized enzymes that cleave amide bonds, with 42%-43% identity to metal-dependent dipeptidases [42, 43] and more distant homology to N-acetyl-L,L-diaminopimelate deacetylase. Echvi_1427 has conserved the metal-binding residues as well as an arginine that binds a terminal carboxylate of the substrate (R264 aligns to R260 from 4EWT; [42]). These features are consistent with activity on N-succinyl-L,L-diaminopimelate.

To test the role of Echvi_1427 and its homolog CA265_RS23900 (from *Pedobacter sp.* GW460-11-11-14-LB5), we used AT978, a strain of *E. coli* that is auxotrophic for lysine due to the *dapE9* mutation of *dapE* [44]. We sequenced *dapE9* and found that codon 78 (out of 375) was mutated to a stop codon (TGG to TGA). In minimal M9 medium, AT978 carrying a control plasmid (expressing RFP) grew slightly or not at all, while AT978 carrying either Echvi_1427, CA265_RS23900, or wild-type *dapE* from *E. coli* grew robustly (Fig 5E). (These experiments used the aTc-inducible vector pBbA2c, but no aTc was added.) This confirms that Echvi_1427 and its homologs are the missing N-succinyl-L,L-diaminopimelate desuccinylases. Of the representative genomes of Bacteroidota in *fast.genomics* [23], the majority (391 of 690) encode orthologs of Echvi_1427 and not *E. coli* DapE (BLAST bit score ratios of at least 0.4 for Echvi_1427 and under 0.3 for E. coli DapE).

### Putative bifunctional MetB/MetC in Xanthomonadales

Methionine biosynthesis requires the synthesis of homocysteine, which is usually formed from activated homoserine. In most Gammaproteobacteria, the homoserine is first activated to O-succinylhomoserine by MetA. Then, O-succinylhomoserine is converted to homocysteine, either by direct sulfhydrylation, which is catalyzed by MetZ, or by transsulfuration, in which MetB (cystathionine gamma-synthase) condenses O-succinylhomoserine with cysteine to form cystathionine and MetC (cystathionine beta-lyase) cleaves cystathionine to yield homocysteine (Fig 6A). However, genetic data from Xanthomonadales suggests that methionine synthesis in these bacteria involves a homolog of MetB, but no MetC-like protein.

**Fig 6:**
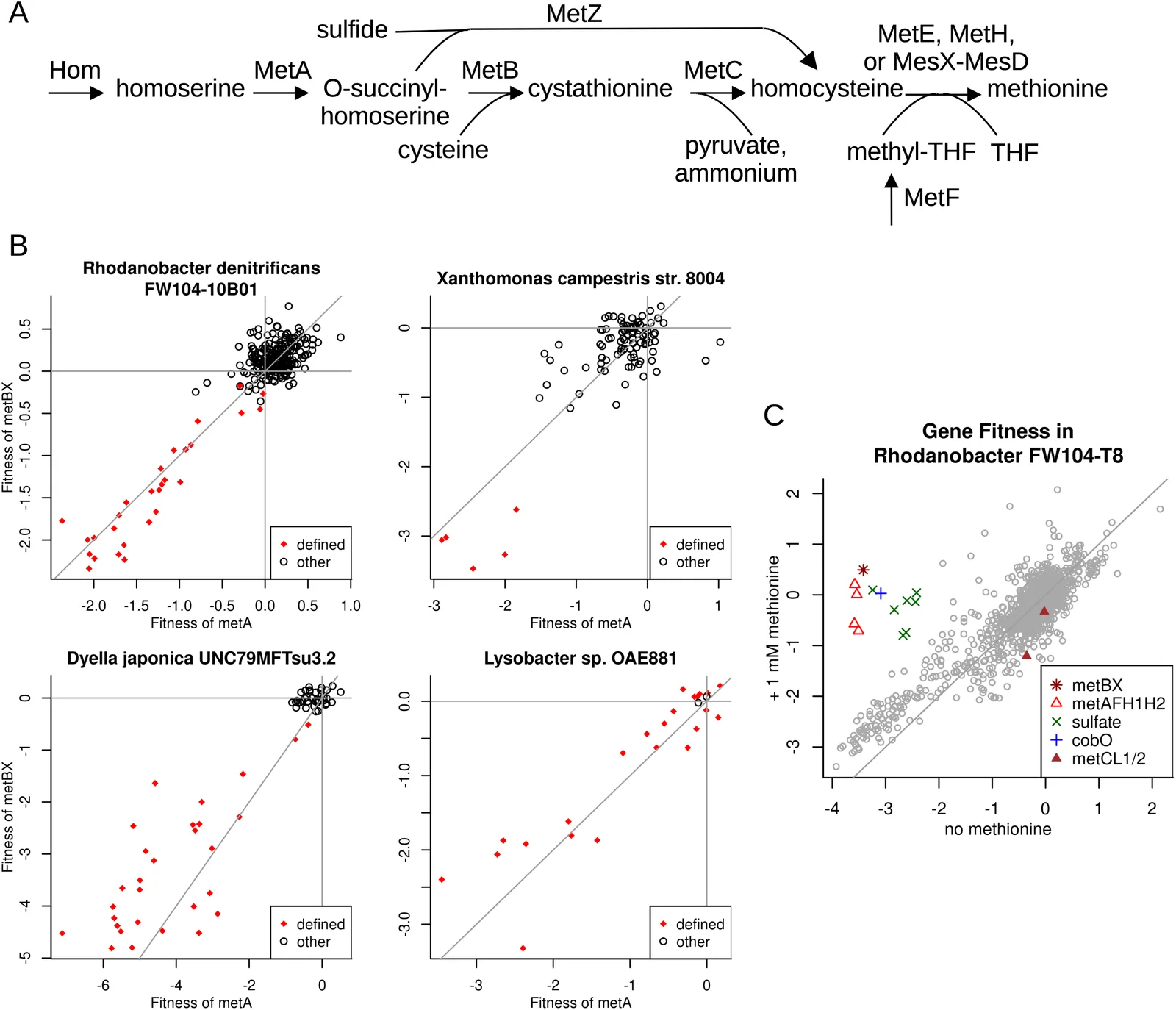
Homocysteine synthesis in Xanthomonadales requires *metBX.* **(A)** Two alternative pathways for the conversion of O-succinylhomoserine to homocysteine in Gammaproteobacteria. **(B)** Fitness patterns of *metA* and *metBX* from representatives of four genera of Xanthomonadales. Each point is an independent assay, and experiments conducted in defined media are highlighted in red. For *Rhodanobacter denitrificans* FW104-10B01, the defined medium often included 100 μM of each amino acid (including L-methionine). **(C)** Fitness data from *Rhodanobacter sp.* FW510-T8 grown in a defined minimal medium with or without 1 mM added L-methionine. The medium included 20 mM D-glucose as a carbon source, ammonium as the nitrogen source, sulfate as the sulfur source, vitamins, and inorganic salts. The *x* axis shows the average of 4 replicate experiments, and the *y* axis shows the average of 3 replicate experiments. Lines show *x* = 0, *y* = 0, or *x =* ***y*.** For the identifiers of the genes highlighted in panels B and C, see S1A Table.

First, in four genera of Xanthomonadales, we identified a *metB*-like gene that has a similar fitness pattern as *metA* (Fig 6B). We named these genes *metBX*, for Xanthomonadales *metB*. (The encoded proteins are 57–60% identical to MetB from *E. coli* and were annotated as MetB by the original GapMind.) To quantify the similarity of fitness patterns of *metA* and *metBX*, we use the “cofitness” or linear (Pearson) correlation, which ranged from 0.79 to 0.94. *MetBX* is usually encoded between *metA* and *hom* (which is responsible for the synthesis of homoserine).

Second, we did not find a *metC*-like gene that was involved in methionine synthesis in these four Xanthomonadales. Each of these genomes does encode two MetC-like proteins, which we will call MetCL1 and MetCL2 (for MetC-like). However, none of these genes were cofit with *metA* (cofitness = -0.65 to -0.30) or with *metBX* (cofitness = -0.78 to -0.26). Prior studies confirm that MetCL1 is not involved in methionine synthesis. In *Xanthomonas oryzae,* a mutant of MetCL1 (PXO_03157) is not required for growth in minimal media, and the mutant has a slight deficiency in growth in the absence of cysteine, not methionine [45]*.* A study of another strain of *X. oryzae* found that a mutant of MetCL1 (XOO0778) had reduced exopolysaccharide and a loss of O antigen [46]. In a large-scale screen for genes that can complement Δ*metB* or Δ*metC* strains of *E. coli* when overexpressed, the *metCL1* gene from *Rhodanobacter denitrificans* (LRK54_RS05660) did not complement Δ*metC*, although it did complement *ΔmetB* [47]. A biochemical study of MetCL1 from *X. oryzae* found that when incubated with cystathionine, it catalyzed the formation of both cysteine and homocysteine [48]; if this enzyme were adapted to a role in methionine synthesis, then it should form homocysteine only, as is thought to be the case for MetC from *E. coli* [49]. Although we did not find prior data on MetCL2, MetCL2 is often encoded in the same apparent operon as MetCL1, and they have similar fitness patterns in three of the bacteria we studied (cofitness = 0.75 to 0.84; fitness data is not available for *metCL1* in *Dyella japonica*). Overall, it seems that MetCL1 and MetCL2 are involved in cell wall synthesis and are unlikely to provide the missing cystathionine beta-lyase activity.

Third, to search for additional genes that might be involved in methionine synthesis, we assayed a mutant library of *Rhodanobacter sp.* FW510-T8 in minimal media with or without 1 mM of added methionine (Fig 6C). We identified strong effects of methionine on gene fitness for *metA, metBX,* cobalamin-dependent methionine synthase *metH* (split into two genes), and *metF* (which forms 5-methyltetrahydrofolate, the methyl donor for methionine synthase). We also found strong differences for genes that are involved in sulfate assimilation (the enzymes *cysDGHIJNC* and a putative *cysB*-like regulator OKGIIK_05975); sulfate assimilation may no longer be necessary if sulfur is obtained from methionine. The only remaining gene with a strong effect of methionine on its fitness was a putative ATP:cob(I)alamin adenosyltransferase (*cobO*, OKGIIK_11600), which is not explained. We did not find any new candidate genes for methionine synthesis.

Based on the genetic data, it appears that MetBX alone, without the aid of another protein, catalyzes the conversion of O-succinylhomoserine to homocysteine in Xanthomonadales. Because MetB and MetC are related enzymes, MetBX could be bifunctional, with cysteine as the sulfur source and cystathionine as an intermediate. Alternatively, it could be an O-succinylhomoserine sulfhydrylase like MetZ, since MetZ is also similar to MetBX (for instance, MetZ from *Pseudomonas aeruginosa* is 42% identical to MetBX from FW510-T8). Structures of MetBX from *X. oryzae* in complexes with aminoacrylate and cysteine or with cystathionine (PDB:6LD8, PDB:6LD9; there is no associated publication) suggest that MetBX could use cysteine as a substrate and that cystathionine could be an intermediate. (MetBX from *X. oryzae* is 94% identical to the MetBX from *X. campestris* in Fig 6B.) So, we predict that Xanthomonadales use a single bifunctional protein to perform both steps in the transsulfuration pathway. This would represent the ancestral state before the divergence of MetB and MetC.

### An alternative N-acetylornithine deacetylase

A recent study proposed that an uncharacterized protein in *Veillonella denticariosi* AS16 (UniProt W3Y6L2) is an alternate N-acetylglutamate synthase because of its conserved proximity to arginine synthesis genes (“ArgA3” in [12]). However, W3Y6L2 is 42% identical to an N-acetyl-cysteine deacetylase (SndA from *Bacillus subtilis*; [50,51]), and the only analogous reaction in arginine synthesis is N-acetylornithine deacetylase (ArgE). Indeed, using *fast.genomics*, we found that representative genomes with closer homologs of W3Y6L2 (11 genera contain a homolog with a bit score ratio above 0.4) never contained likely ArgE proteins (homologs of *E. coli* ArgE with a bit score ratio above 0.2). So, we propose that W3Y6L2 is an alternative N-acetylornithine deacetylase.

We cloned W3Y6L2 into the aTc-inducible expression vector pBbA2c and found that it rescued growth of an *argE::kan* strain of *E. coli* in M9 minimal medium under anaerobic conditions (Fig 7). The *argE::kan* + W3Y6L2 strain did not grow in the same medium aerobically. Since *V. denticariosi* is strictly anaerobic [52], its enzymes might not have evolved to tolerate oxygen. Alternatively, homologs of W3Y6L2 are variously reported to use a Zn^2+^ or Co^2+^ cofactor (SndA, 42% identity [51],) or a Ni^2+^ cofactor (PDB:1YSJ, 38% identity). Since *E. coli* only uses nickel under anaerobic conditions (for [NiFe] hydrogenases), it is possible that W3Y6L2 requires a Ni^2+^ cofactor which is not available in aerobically-grown *E. coli*.

**Fig 7:**
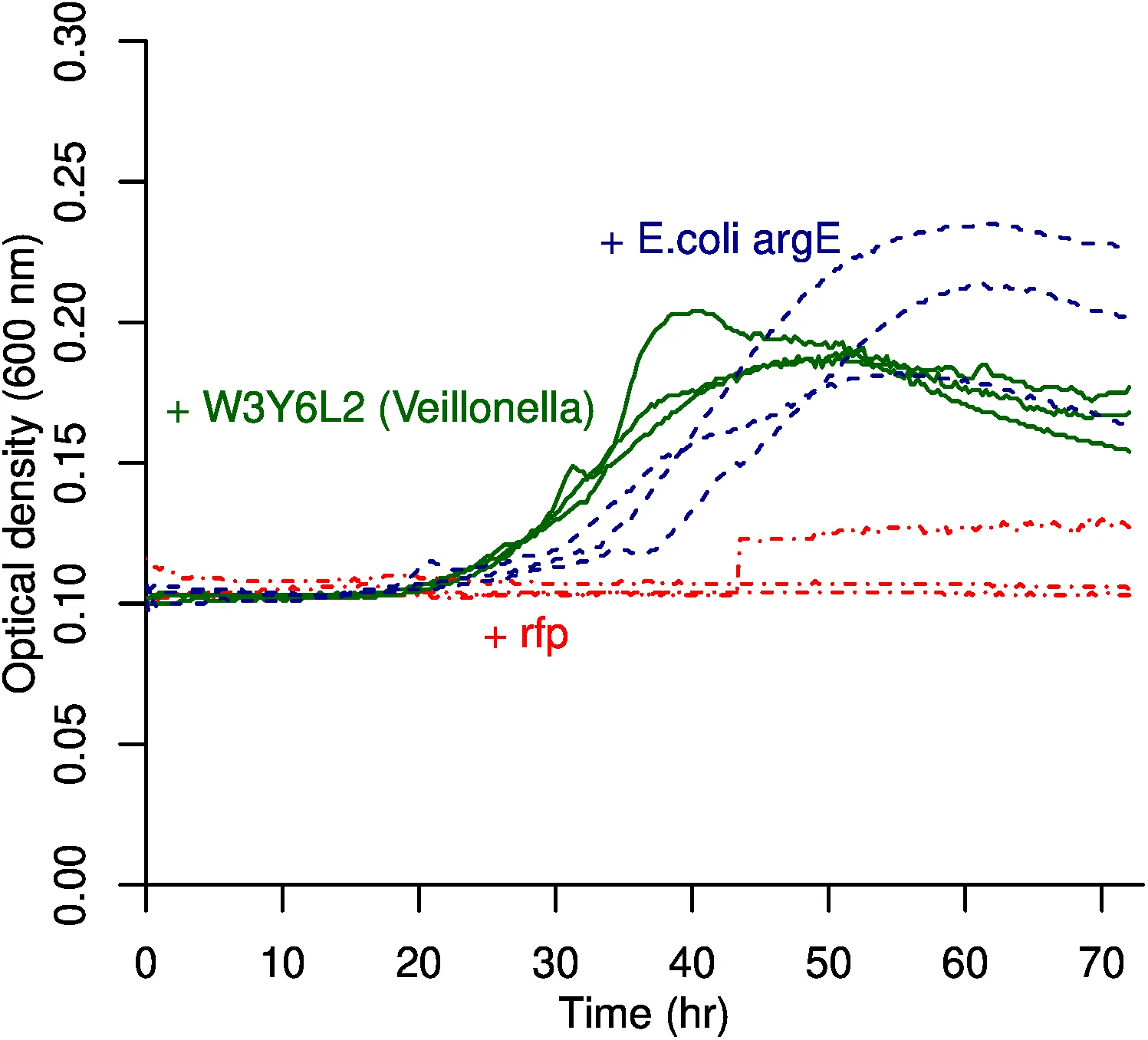
W3Y6L2 from *Veillonella denticariosi* complements an *argE* mutation of *E. coli.* We show the growth of *argE::kan E. coli* with plasmids carrying *rfp*, *argE* from *E. coli,* or W3Y6L2 from *Veillonella denticariosi*. All strains were grown anaerobically in M9 minimal medium with 20 mM glucose, Wolfe’s minerals, Wolfe’s vitamins, 4 nM anhydrotetracycline as an inducer, and 20 μg/ml chloramphenicol to retain the plasmid. For each strain, all three technical replicates are shown.

### A predicted alternative N-succinyl-L-glutamate synthase

In *Steroidobacter denitrificans* DSM 18526, the N-succinyl-L-glutamate synthase (ArgA) is missing. The gene cluster for arginine biosynthesis in *Steroidobacter* and related bacteria includes a very distant homolog (ACG33_RS14135) of a D-glutamate acetyltransferase [53], so we predict that ACG33_RS14135 encodes the missing N-succinyl-L-glutamate synthase. For details, see S1B Text.

### Previously-proposed alternate enzymes

The revised GapMind also includes a number of previously-proposed alternate enzymes (Table 2). Six of these already had experimental evidence for their role; we obtained experimental evidence for three additional enzymes; and the other four await experimental tests. All of the predictions are described in S1C Text and S1D Text; here we briefly describe the new experimental evidence.

**Table 2:** Alternate enzymes that were previously proposed and are included in the revised GapMind.

Pathway	Step	Evidence	Genus	UniProt	Reference
Met	Sulfur carrier protein (MetO)	yes	*Streptomyces*	A0A059W7F4	[14]
Met	[MetO]-L-cysteine hydrolase (Mec)	yes	*Streptomyces*	A0A059W3G7	[14]
Met	[MetO]-sulfur ligase (MoeZ)	yes	*Streptomyces*	A0A401R754	[14]
Met	MetO-thiocarboxylate-dependent homocysteine synthase (MetM)	yes	*Streptomyces*	A0A059WBP2	[14]
Pro	Ornithine cyclodeaminase	yes	*Methanococcus*	Q6LXX7	[54]
Ser	Phosphoserine transaminase (SerC2)	yes	*Staphylococcus*	Q2FXK2	[12,38]
Thr	Homoserine phospho- transferase (HomK)	this work	*Bacteroides*	Q8A542	[2]
Ser	Phosphoserine transaminase (SerC3)	this work	*Clostridium*_*F*_	A5I0W7	[12]; this work
Lys	Diaminopimelate epimerase (DapF2)	this work	*Staphylococcus*	A0A9Q5JK70	[12]
Lys	N-acetyl-diamino-pimelate deacetylase (DapL)	predicted	*Staphylococcus*	Q2FH40	[12,55]
Met	Split MetE, folate- binding component	predicted	*Pyrolobus*	G0EDA1	[13]
Met	Split MetE, catalytic component	predicted	*Pyrolobus*	G0EFB7	[13]
Met	Corrinoid-dependent methionine synthase (MesC)	predicted	*Methanosarcina*	Q8TUL3	[13]

First, we previously reported that a putative alternative to homoserine kinase (BT2402) is required for threonine synthesis in *Bacteroides thetaiotaomicron* [2]. Close homologs of BT2402 (over 80% identical) are involved in threonine synthesis in the genus *Phocaiecola* (data of (Huang et al. 2024); S1C Text). We cloned BT2402 into a *thrB::kan* strain of *E. coli* and found that it could rescue growth in minimal medium glucose medium with aTc: OD_600_ increased by 0.14 after 47 hours (3 replicates; see S1C Text). This confirms that BT2402 and related proteins (also known as TIGR02535 [9] or ThrB2 [12]) are alternatives to homoserine kinase. BT2402 is related to cofactor-independent phosphoglycerate mutases and probably uses a different phosphate donor than ATP, perhaps phosphoserine (S1C Text). Because we predict that BT2402 catalyzes a different reaction than homoserine kinase (ThrB), it is included in GapMind as a different step. We chose the name HomK, as it is an alternative to the usual homoserine kinase (ThrB).

Second, Ashniev and colleagues associated a family of putative transaminases, which they named SerC3, with serine synthesis [12]. We cloned the representative of this family from *Clostridium*_*F*_ *botulinum* (UniProt A5I0W7) into a *serC::kan* strain of *E. coli* [27] and found that it rescued growth in minimal medium: in M9 glucose medium with aTc, the OD_600_ increased by 0.16-0.18 after 48 hours (3 replicates; see S1D Text). This confirms that *serC3* proteins are phosphoserine transaminases.

Third, Ashniev and colleagues associated a family of putative epimerases, which they named DapF2, with lysine synthesis, and predicted that they are diaminopimelate epimerases (Ashniev et al. 2022). This family includes the Alr2 protein from *Staphylococcus aureus*, which is often annotated as alanine racemase, but Alr2 is not involved in D-alanine synthesis in *S. aureus* (Panda et al. 2024). To test the role of *dapF2*, we cloned the gene from *S. epidermidis* (UniProt A0A9Q5JK70 = HMPREF2922_03580) into a Δ*dapF* strain of *E. coli* (see Methods). The Δ*dapF* strain does not have a growth defect, but strains of *E. coli* that lack *dapF* are reported to secrete L,L-diaminopimelate (which is the substrate of DapF) into the medium [56]. It is thought that in Δ*dapF* cells, L,L-diaminopimelate is overproduced, and some other racemase converts L,L-diaminopimelate to *meso*-diaminopimelate (the precursor of both peptidoglycan and lysine) with low efficiency, while the excess L,L-diaminopimelate is secreted [56].

We used LC-MS/MS to examine the supernatant from various strains of *E. coli*, including a *dapF* deletion and strains with *E. coli dapF* or *dapF2* expressed from the aTc-inducible vector pBbA2c. Diaminopimelate (DAP) was identified in the supernatant from the *dapF* mutant by comparing the mass to charge ratio, retention time and fragmentation spectra of samples to a pure reference standard (Fig 8A & 8B; see Methods for the identification of DAP from other supernatants). (Mengin-Lecreulx and colleagues reported that L,L-DAP, which is the substrate of DapF, was secreted, while our method did not distinguish between L,L-DAP or *meso*-DAP.) Furthermore, complementation with *dapF2* yielded similar results as wild-type cells, whether or not the inducer aTc was added (Fig 8C & 8D). In the absence of inducer, complementation with *dapF* from *E. coli* yielded similar results as wild-type, but in the presence of inducer, expression of *dapF* from a plasmid yielded intermediate results; we speculate that this results from overly high expression of DapF. Overall, this experiment confirmed that *dapF2* can complement a *dapF* mutant, and supports the prediction that DapF2 is a diaminopimelate epimerase.

**Fig 8:**
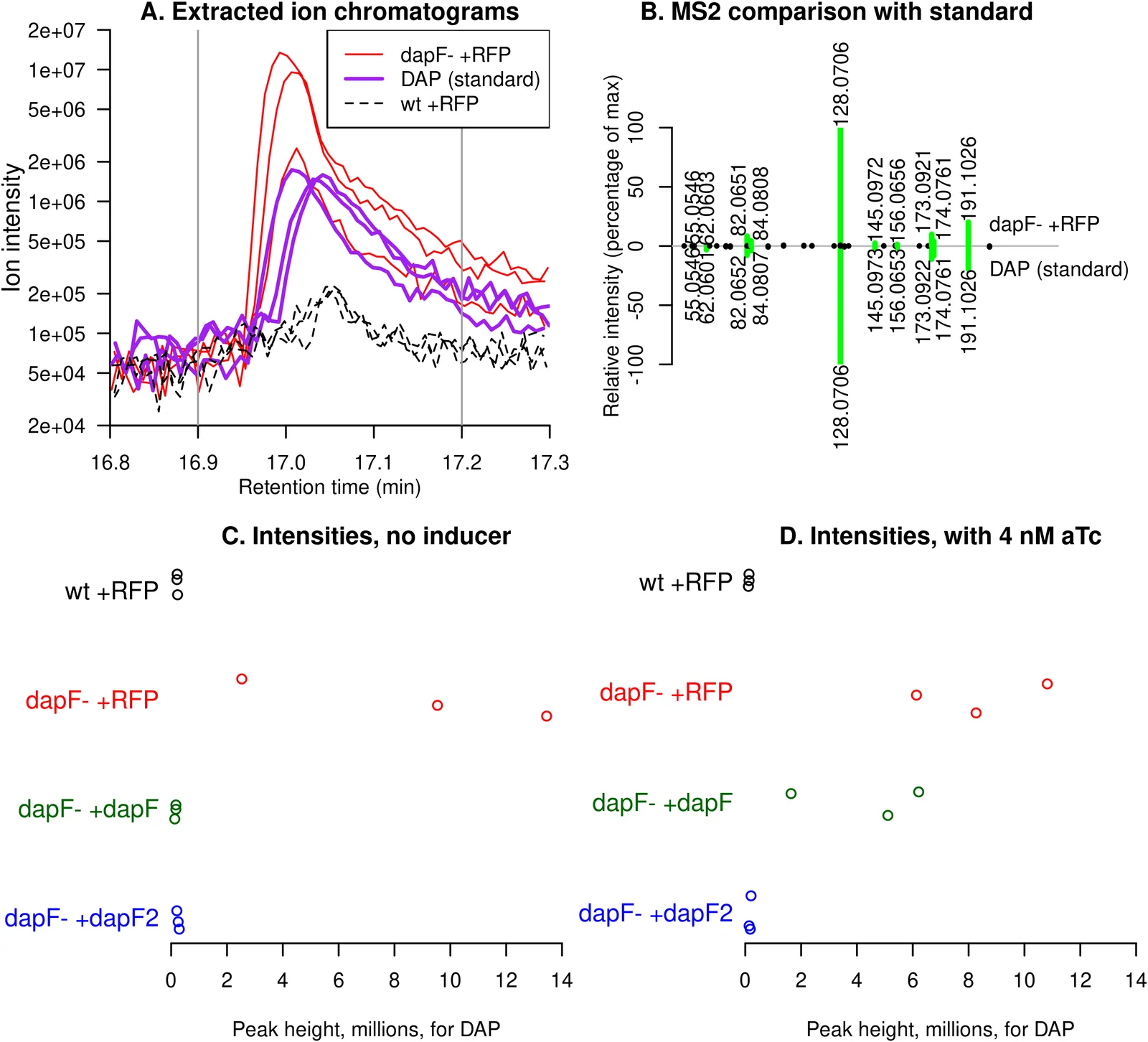
LC/MS analysis of DAP secretion by *dapF* mutants of *E. coli* and the effect of *dapF2.* **(A)** Extracted ion chromatograms for the DAP **[M-H]**+ adduct (*m/z* = 191.1026, 10ppm window) in supernatant from a *dapF* mutant. Note log *y* axis. A peak at the theoretical mass to charge ratio for DAP elutes at the same retention time as a DAP standard. The vertical bars show the range of retention times used to measure the peak heights (below). **(B)** A mirror plot compares the fragmentation spectrum from ΔdapF supernatant (above the line) and the standard (below the line). Both spectra are normalized to their respective maximum intensities. Matching fragments (*m/z* within 15 ppm) are in green; others are in black. **(C)** Peak heights for the supernatant from various strains grown without inducer. **(D)** Similarly for cultures grown with 4 nM aTc. The strains were: wild-type (*E. coli* MG1655) with a control plasmid expressing RFP; a clean deletion of *dapF* in the MG1655 background, also with the control plasmid; ΔdapF with E. coli *dapF* expressed on a plasmid; and ΔdapF with *dapF2* from *S. epidermis* (UniProt:A0A9Q5JK70, HMPREF2922_03580) expressed on a plasmid. Expression from all plasmids was inducible by aTc.

DapF2 is in a conserved operon with putative amidohydrolases, such as AA076_RS07060 (UniProt Q2FH40). These amidohydrolases have been proposed to be the missing N-acetyl-diaminopimelate deacetylase DapL [12,55]. AA076_RS07060 is 35% identical to N-acetyl-L-cysteine deacetylase SndA, which acts on a similar substrate, and has conserved catalytic residues.

### Diverged enzymes

GapMind only considers a protein to be a high-confidence candidate for a step if it is at least 40% identical to a characterized enzyme. (For enzymes that have curated models in TIGRFam, high-confidence candidates can also be identified via the hidden Markov model, but 38% of steps in GapMind are not associated with any TIGRFam, and non-canonical enzymes are usually not described in TIGRFam.) To improve the coverage of GapMind, we searched for diverged enzymes that had experimental evidence but were missing from the curated databases that GapMind relies on. We also used fitness data to confirm seven enzymes, and we used complementation assays to confirm another three (S1E Text). One of the enzymes that we confirmed by complementation is a phosphoserine transaminase (SerC, UniParc A0A843E9R6) from a metagenome-assembled genome of a Methanomethylophilaceae. As far as we know, this family of archaea has not yet been cultured yet, but we were still able to fill this gap in serine biosynthesis. Overall, we added 19 diverged enzymes with experimental evidence to GapMind (Table 3).

**Table 3:** Diverged enzymes with experimental evidence that are included in the revised GapMind.

Pathway	Step	Genus	Identifier	Reference
Arg, Lys	LysZ	*Thermococcus*	TK0276	[57]
chorismate	AroA	*Bacteroides*	BT_RS11065	[47]
chorismate	AroE	*Sphingomonas*	BDW16_RS10815	[47]
His	HisC	*Acidovorax*	AAFF19_05770	[47]
His	HisN	*Bifidobacterium*	A0A0L7BRC5	[16]
Ile, Val	IlvH	*Rhodanobacter*	LRK54_RS10305	This work
Ile, Val	IlvH	*Lysobacter*	ABIE51_RS17555	This work
Ile, Val	IlvH	*Xanthomonas*	Xcc-8004.1058.1	This work
Met, Thr	Hom	*Brevundimonas*	A4249_RS00005	This work
Phe	Prephenate dehydratase	*Brevundimonas*	A4249_RS06835	This work
Pro	ProB	*Pedobacter*	AAFF35_11135	[47]
Ser	SerB	*Lysobacter*	GGC55_RS00335	This work
Ser	SerB	*Xanthomonas*	A0A0H2XA29	This work
Ser	SerC	*Paucidesulfovibrio*	A0A1T4W7T3	This work
Ser	SerC	*Methanomethylo-philaceae*	A0A843E9R6	This work
Thr	ThrB	*Bacillus*	BSU_32240	[47,58]
Thr	HomK	*Dehalococcoides*	DET_RS08350	This work
Trp	TrpA	*Pedobacter*	AAFF35_06155	[47]
Trp	TrpA	*Rhodanobacter*	LRK54_RS01680	[47]

We identified another 28 diverged enzymes that lack experimental evidence but are confirmed by conserved gene context and are required to explain the biosynthesis of the amino acid in a prototrophic prokaryote (Table 4). For these predicted enzymes, where possible, we also checked that catalytic residues were present, using SitesBLAST ([59]; for functional residues, see S1B Table). The highly-diverged regulatory subunit for acetolactate synthase (IlvH) from *Korarchaeum cryptofilum* was checked using structural analyses (S1F Text). Three diverged HomK enzymes were identified by the combination of being encoded near *hom* (Table 4) and functional residues (see S1C Text).

**Table 4:** Diverged enzymes that are included as predictions in the revised GapMind.

Pathway	Step	Organism	Identifier
Arg	LysK	*Nitrosopumilus maritimus*	NMAR_RS06940
chorismate	AroD	*Pyrolobus fumarii*	PYRFU_RS04235
chorismate	AroA	*Pyrolobus fumarii*	PYRFU_RS00635
Gln	GlnA	*Clostridium kluyveri*	CKL_RS00630
Lys	LysA	*Chitinovibrio alkaliphilus*	CALK_RS03395
Lys	LysZ	*Haloferax volcanii*	D4GYN9
Ile, Val	IlvH	*Brevundimonas sp.*	A4249_RS10015
Ile, Val	IlvH	*Chryseobacterium sp.*	A0A316WEF5
Ile, Val	IlvH	*Korarchaeum cryptofilum*	KCR_RS03285
Met	Split MetE, folate-binding	*Haloferax volcanii*	D4GW95
Met	Split MetE, catalytic	*Haloferax volcanii*	D4GW90
Met	B12 reactivation	*Heliobacterium modesticaldum*	H1S01_RS06050
Met	L-aspartate semialdehyde S-transferase, NIL/ferredoxin	*Methanococcus vannielii*	MEVAN_RS03425
Met	L-aspartate semialdehyde S-transferase, persulfide-form.	*Hippea alviniae*	G415_RS0107280
Met	Mec	*Dissulfurispira thermophila*	A0A7G1H0A1
Phe	Prephenate dehydratase	*Carboxydothermus pertinax*	A0A1L8CUL6
Ser	SerA	*Ideonella sp.*	A0A257CND8
Ser	SerA	*Roseburia faecis*	A0A374I5P9
Ser	SerA	*Rhodoferax sp.*	A0A975E934
Ser	SerBK	*Sediminibacterium sp.*	A0A521P7R6
Ser	SerC	*Thermoanaerobacter kivui*	A0A097AUI2
Thr	HomK	*Paramuribaculum intestinale*	A0A2V1IX31
Thr	HomK	*Microtrichaceae sp.*	DCC48_11680
Thr	HomK	*Megalodesulfovibrio gigas*	T2GDP6
Tyr	Prephenate dehydrogenase	*Dehalococcoides mccartyi*	DET_RS02500
Tyr	Prephenate dehydrogenase	*Desulfarculus baarsii*	E1QE65
Tyr	Prephenate dehydrogenase	*Desulfobulbus oligotrophicus*	A0A7T5VEL0
Tyr	Prephenate dehydrogenase	*Nitrosopumilus maritimus*	A9A228

### The accuracy of GapMind for prototrophs

To test the coverage of the revised GapMind, we analyzed the predicted proteomes of 208 diverse bacteria and archaea that can grow in defined minimal medium (see Methods). We ensured that the growth data and the genome sequence were from the same strain. These 208 prototrophs cover 20 phyla and 163 genera from the Genome Taxonomy Database [60]. Across 3,744 pathway x organism combinations, 84% were fully complete and consisted only of high-confidence steps. Another 11% of pathways contained one or more medium-confidence steps, such as a comparative genomics prediction or a diverged enzyme. (Unless the step is described by a hidden Markov model, high-confidence candidates must be at least 40% identical to a characterized enzyme.) 5% of pathways had one or more gaps, with no high- or medium-confidence candidate for at least one step.

Overall, we identified 187 gaps in these 208 genomes. However, for 37 of these gaps, a high- or medium-confidence candidate was identified when analyzing the six-frame translation of the genome. Most of these discrepancies were due to missing gene calls, but these cases also include seven distinct frame shifts that led to 13 gaps. (Some steps occur in more than one pathway.) We previously showed that three putative frame shifts in amino acid biosynthesis genes from prototrophic bacteria were due to errors in their genome sequences [2,4]. That includes one of the seven frame shifts in our current analysis, but we expect that the other six are spurious as well. For example, one of the genomes with the most gaps was *Desulfotalea psychrophila* LSv54, which is a sulfate-reducing bacterium that grows with lactate as the sole carbon source [61]. The genome has apparent frameshifts in *ilvC, ilvD*, *aroB*, and L,L-diaminopimelate aminotransferase. The genome was sequenced at only 6.4x coverage [61], which suggests that these frameshifts could be spurious. This genome also has three genuine gaps: phosphoribosyl-ATP pyrophosphatase *(hisI*), histidinol-phosphate phosphatase (*hisN*), and phosphoserine phosphatase (*serB*) are all missing. Overall, we found 150 genuine gaps, corresponding to 0.7% of all steps on the best paths.

If we consider each step once, even if it occurs in multiple pathways, then the 150 genuine gaps become 143 unique gaps across 208 genomes, for an average of 0.7 per genome. In contrast, the original GapMind had 282 unique gaps for these genomes (an average of 1.4). Just four steps account for 65% of the 143 remaining gaps: histidinol-phosphate phosphatase *hisN* is missing in 42 genomes (20% of prototrophs), phosphoribosyl-ATP pyrophosphatase *hisI* is missing in 19 genomes (9%), phosphoserine phosphatase *serB* is missing in 16 genomes (8%), and homoserine kinase *thrB* is missing in 16 genomes (8%). All four of these enzymes catalyze the gain or loss of phosphate or pyrophosphate groups, which can be carried out by many different protein families. We speculate that other kinases, phosphatases, or pyrophosphatases have evolved to act on these substrates and sometimes replace the known families. In any case, the absence of these genes should not be used to predict auxotrophies.

As shown in Fig 9, most prototrophic genomes from the phylum Pseudomonadota do not have any genuine gaps. This is probably because Pseudomonadota is the best-studied phylum. (Of the 163 known prototrophic genera in our collection, 77 are Pseudomonadota.) In contrast, most of the other bacteria and archaea that we studied have at least one genuine gap in their amino acid biosynthesis pathways. Even for the Pseudomonadota, the true rate of gaps may be higher, as we used the same set of prototrophic genomes to help us fill gaps in biosynthetic pathways. If we exclude all of the genera that we have studied, either in the experiments or computational analyses above or in other RB-TnSeq studies, then the average number of gaps per genome in Pseudomonadota rises from 0.18 to 0.26, which suggests some bias. In other words, we filled a higher proportion of the gaps in the studied Pseudomonadota than in the typical Pseudomonadota. But for other taxa, excluding the genera that we studied makes little difference (the average goes from 1.27 to 1.26).

**Fig 9:**
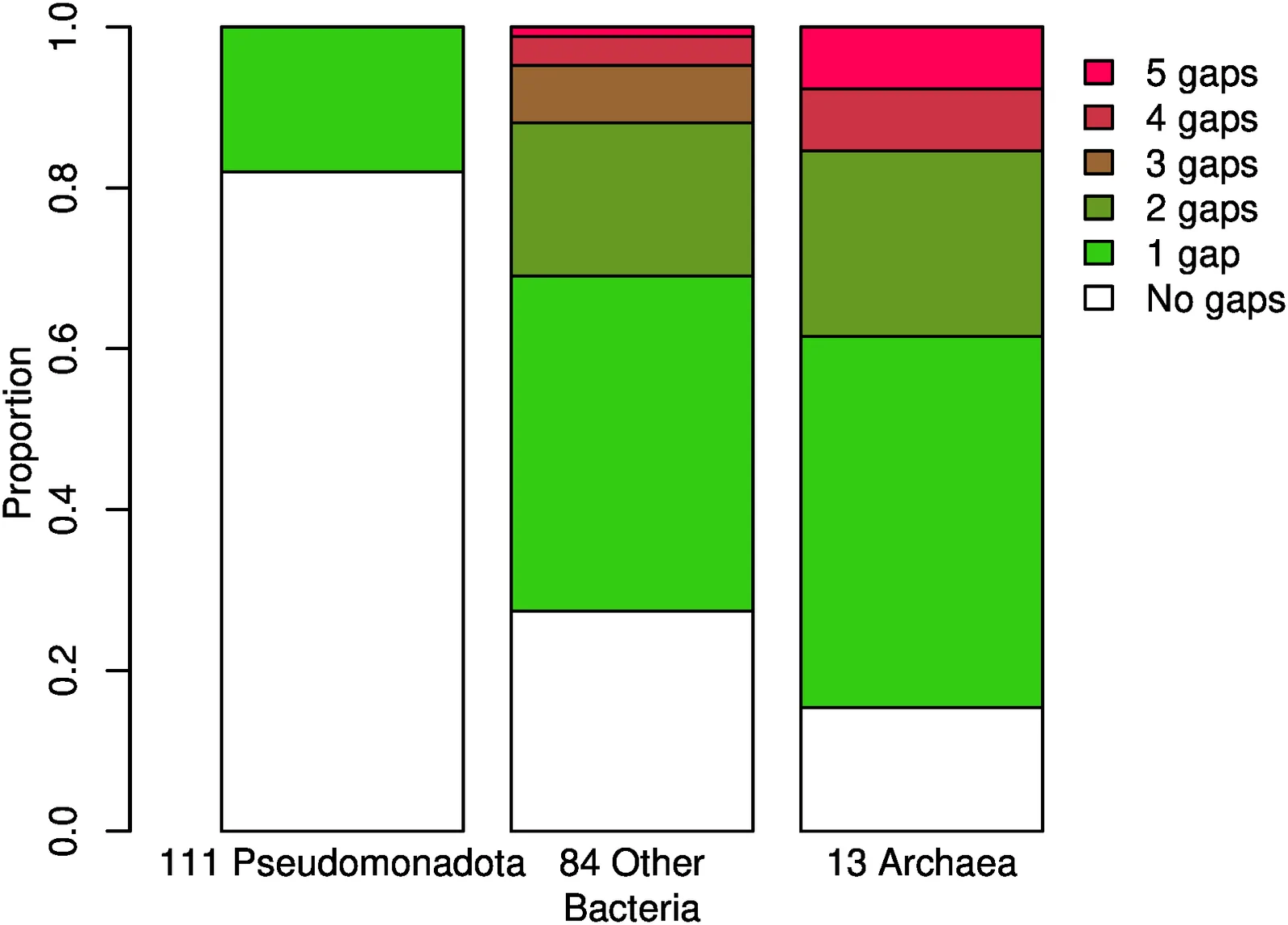
Gaps per genome in the amino acid biosynthesis pathways of prototrophic bacteria and archaea. Gaps that are due to missing gene calls or frameshifts (which may be spurious) were not included. If the same step was missing in multiple pathways, it was counted just once.

### The accuracy of GapMind for auxotrophic bacteria

We also tested GapMind on prokaryotes with known auxotrophies. We identified 26 prokaryotes (all bacteria) with experimentally-confirmed requirements for one or more amino acids (see Methods). These gave a total of 106 genome x pathway combinations for which the pathway was reported not to be functional. GapMind found that 102 of these (96%) had at least one gap or low-confidence step. This contrasts to the rate of 5% for pathways in prototrophic bacteria. Most of the non-functional pathways (76%) had more than one gap, versus just 0.4% of pathways from prototrophic bacteria.

We manually examined the four cases where the GapMind results might incorrectly imply that an auxotrophic strain was prototrophic for that amino acid. Three of these cases involve *Streptococcus pneumoniae* D39. As previously reported, *S. pneumoniae* appears to contain all of the genes necessary for isoleucine and valine synthesis, and it is not clear why these amino acids are required for growth [21]. Also, *S. pneumoniae* is auxotrophic for glycine even though it encodes a likely serine hydroxymethyltransferase (*glyA,* SPD_RS04890), which in most bacteria converts serine to glycine. As discussed above, it appears that *S. pneumoniae* does not synthesize or take up serine, and its GlyA functions in reverse, to convert glycine to serine [22].

Finally, *Fusobacterium varium* ATCC 27725 requires methionine for growth [62]. Although GapMind did not identify any low-confidence steps in methionine synthesis in *F. varium*, two steps were medium-confidence: homoserine dehydrogenase and cystathionine gamma-synthase (*metB*). The potential homoserine dehydrogenase (C4N18_RS12320) is only 39% identical to a characterized homoserine dehydrogenase, but the active-site residues are conserved and C4N18_RS12320 is encoded in an apparent operon with two other genes for homoserine biosynthesis (aspartate kinase and aspartate-semialdehyde dehydrogenase). So we expect that C4N18_RS12320 is truly a homoserine dehydrogenase. Furthermore, *F. varium* grows in the absence of threonine [62], which suggests that homoserine dehydrogenase (which is usually required for threonine synthesis) is present. For *metB*, GapMind identified two candidates, which are medium-confidence because they have greater similarity to methionine gamma-lyases (73–77% identity) than to known MetB proteins (44–48%). Given the growth data for *F. varium*, these proteins probably lack MetB activity. Overall, if a bacterium was reported to require an amino acid for growth, GapMind identified at least one low-confidence step in that pathway 96% of the time.

## Conclusions

The revised GapMind for amino acid biosynthesis includes novel genes for the synthesis of arginine, glycine, lysine, methionine, serine, and branched-chain amino acids, as well as many diverged enzymes. Across prototrophic bacteria and archaea, 95% of pathways are complete, with no low-confidence steps, while for experimentally-identified auxotrophies, 96% of pathways had at least one low-confidence step. The majority of the remaining gaps in functioning pathways are due to just four steps: *hisN*, *hisI, serB,* and *thrB*.

## Materials and methods

### Updating GapMind

Besides including the additional characterized enzymes and predictions described above, we also updated GapMind to use newer versions of the curated databases Swiss-Prot [6], MetaCyc [7], BRENDA [8], and the Fitness Browser reannotations [11,40]. These were updated in November 2023 (Swiss-Prot release 2023_05, BRENDA release 2023.1, and MetaCyc 27.1, which is the last public release). (GapMind also uses CharProtDB, which, as far as we know, has not been updated [63].)

GapMind for amino acid biosynthesis now includes 159 different biosynthetic steps. These are described by 2,261 characterized proteins from curated databases, 138 characterized proteins that we curated, 4,469 curated predictions from Swiss-Prot, and 30 proteins whose function we predicted. Candidates that were identified from their similarity to proteins with predicted functions -- either from the predictions described above, or from curated annotations in Swiss-Prot that lack experimental evidence -- are treated as one level of confidence lower than if they were based on similarity to a characterized protein. For instance, if a candidate is at least 40% identical to a protein with a predicted function, and the alignment covers at least 70% of the protein with a predicted function, then the candidate will be considered medium confidence.

GapMind also uses hidden Markov models of protein families to describe some steps. These are based on 141 TIGRFams [9] and four PFams [20].

When run from the command-line, GapMind now provides the option to use DIAMOND [64] instead of UBLAST [65] for pairwise searches. DIAMOND is not as fast as UBLAST, but is more sensitive. Because GapMind ignores homologs that are less than 30% identity or that cover less than half of the characterized protein, this makes little difference to the final results of GapMind. For the 208 prototrophic genomes, the steps on the best path (according to GapMind’s analysis of the annotated proteins using UBLAST) receive identical confidence scores 99.4% of the time, and DIAMOND reports a similar total number of gaps (199 instead of 187).

### Essential proteins

Essential proteins for *Echinicola vietnamensis* KMM 6221 (DSM 17526), *Pedobacter sp.* GW460-11-11-14-LB5, and *Pontibacter actiniarum* KMM 6156 (DSM 19842) were reported previously [40]. Essential proteins were defined as proteins with unusually low coverage by transposon mutants [40]. Mutants of these genes might grow very slowly, rather than being truly essential. A RB-TnSeq library for *Mucilaginibacter yixingensis* YX-36 was described elsewhere [41]; from this library, 447 essential proteins were identified, using the same approach. Similarly, based on an RB-TnSeq library for *Lysobacter soli* OAE881 (see below), we identified 253 essential proteins.

### Sequence analysis

Characterized homologs were identified using PaperBLAST [66]. Functional residues were identified using SitesBLAST [59]. Conserved gene neighbors and gene co-occurence were identified using the spring 2025 release of *fast.genomics* [23], which has one representative genome for each of 7,312 genera of prokaryotes. RB-TnSeq data was examined using the Fitness Browser [11].

For finding homologs within GapMind, we used UBLAST [65] from 64-bit usearch 11.0.667 (available at https://github.com/rcedgar/usearch_old_binaries/) and HMMer 3.3.1 (http://hmmer.org/; [67]).

Boltz-2x, which is the co-folding prediction tool Boltz-2 with a physical steering potential [29], was invoked with a protein sequence and a SMILES string for the small molecule, using foldy [68]. The predicted structure of GlyXL from *Bifidobacterium breve* (bound to glyoxylate) was very similar to the experimental structure (PDB:2HA9) for GlyXL from *Streptococcus pneumoniae* (root mean square deviation of 1.21 Å, TM-score 0.97, 59% sequence identity), as assessed using RCSB [19].

### Prokaryotes that grow in defined media

We previously reported a list of 148 strains of prokaryotes that grow in minimal media and have complete genome sequences available ([2]; derived from [69,70]). One of those genomes (GCF_000195895.1, for *Methanosarcina barkeri* str. Fusaro) has been removed from Genbank, so we removed it from our list. We also reported a list of 35 bacteria that had RB-TnSeq data during growth in minimal media [2]. Recent studies reported RB-TnSeq data for four additional bacteria growing in minimal media: *Magnetospirillum magneticum* AMB-1 [71], *Mucilaginibacter yixingensis* YX-36 (DSM 26809) [41], *Nitratidesulfovibrio vulgaris* Hildenborough [10], and *Xanthomonas campestris pv. campestris* strain 8004 [72]. For this study, we conducted RB-TnSeq assays for *Brevundimonas vesicularis*_*C*_ GW460-12-10-14-LB2, *Lysobacter soli* OAE881 (DSM 113522), and *Rhodanobacter denitrificans* FW104-10B01 (see below).

We identified additional prototrophic prokaryotes from the literature: *Thermoanaerobacter kivui* LKT-1 [73], *Thermus aquaticus* YT-1 [74], *Haloferax volcanii* DS2 [75], *Nitrosopumilus maritimus* SCM1 [76], *Streptomyces albulus*_*C*_ NBRC14147 [14], and *Dissulfurispira thermophila* T55J [77]. Also, a recent study identified several isolates from the human gut that are prototrophic [78]; we included *Anaerobutyricum hallii* DSM 3353, *Roseburia faecis* M72, and *Clostridium tyrobutyricum* FAM22553. A caveat for these three strains is that cysteine was included in the growth medium as a reductant; however, cysteine synthesis appears to be complete in all three strains, so we expect that they are truly prototrophic. Similarly, cysteine was present in the growth medium for *T. kivui*, but since it is autotrophic [73], it is presumably able to make cysteine.

Finally, to increase the diversity of prokaryotes in our list, we used the IJSEM database [79] to identify representatives of taxonomic orders that were not included in the list, but had been reported to utilize specific carbon sources. We then checked the species descriptions to see if they used a truly defined medium with no added amino acids. By this approach, we identified ten prototrophic type strains (*Algiphilus aromaticivorans* DG1253, *Aquimarina longa* SW024, *Carboxydothermus pertinax* Ug1, *Halococcus hamelinensis* 100A6, *Hippea alviniae* EP5-r, *Methanospirillum lacunae* Ki8–1, *Nocardioides dokdonensis* FR1436, *Phaeacidiphilus oryzae* TH49, *Thiohalospira halophila* DSM 15071 HL 3, and *Tistlia consotensis* USBA 355).

### Experimentally-determined amino acid auxotrophies

Most of the experimentally-determined auxotrophies were taken from previous reports [1,12]. We removed some species because the original reference did not identify which exact strain was auxotrophic, and we wanted to ensure that the genome sequence analyzed was from an auxotrophic strain. We removed some strains because the precise amino acids that were required for growth were not determined. We also identified several strains that were proposed to be auxotrophic for cysteine, but based on experiments with sulfate as the sole source of sulfur, and the pathway for sulfate assimilation is absent from the genome (i.e.[80,81]). In these cases, reduced sulfur compounds such as sulfide may be the natural sulfur source, and if a suitable sulfur source was provided, cysteine synthesis may well occur. Finally, we removed *Klebsiella pneumoniae* KP11 because growth data was not reported and we removed *Yersinia ruckeri* YRB because there was significant residual growth after individual amino acids were removed from the media [3].

We added some additional auxotrophies from the literature: *Cysteiniphilum litorale* DSM 101832 is auxotrophic for cysteine [82]; *Bacillus subtilis* 168 is auxotrophic for tryptophan [83]; *Myxococcus xanthus* DK101 is auxotrophic for all three branched-chain amino acids [84]; *Kytococcus sedentarius* DSM 20547 is auxotrophic for methionine [85]; *Legionella pneumophila* Philadelphia 3 is auxotrophic for arginine, methionine, serine, threonine, and valine [86]; and *Streptococcus pneumoniae* D39 is auxotrophic for arginine, glycine, histidine, isoleucine, leucine, and valine [21].

In total, we had 26 auxotrophic bacteria, with 106 auxotrophies for amino acids. Four of these bacteria were auxotrophic for all three aromatic amino acids. In principle, these bacteria could be auxotrophic for chorismate (the common precursor for the aromatic amino acids) while being capable of converting chorismate to all three aromatic amino acids. This would complicate the comparison of the growth requirements to GapMind’s pathways, because GapMind describes chorismate synthesis separately from the biosynthetic pathways for phenylalanine, tyrosine, and tryptophan. In reality, all four of the bacteria have gaps in all three pathways downstream of chorismate. Two of the four have gaps in chorismate synthesis as well (*Lactobacillus delbrueckii subsp. lactis* CRL581 and *Lactobacillus paracasei* LC2W).

### RB-TnSeq libraries

The mutant libraries for *Brevundimonas vesicularis*_*C*_ GW460-12-10-14-LB2 and *Bacteroides thetaiotaomicron* VPI-5482 were described previously [87].

To construct a mutant library in *Lysobacter soli* OAE881 [88], we used conjugation from *E. coli* WM3064 harboring the pHLL250 *mariner* transposon vector library (strain AMD290), which was previously built via Golden Gate assembly [87]. Specifically, we grew 10 mL of wild-type OAE881 in LB overnight at 30ºC. The next morning, we recovered a 2 mL freezer stock of strain AMD290 in 50 mL LB supplemented with 50 mg/mL carbenicillin (Cb) and 300 µM diaminopimelic acid (DAP) at 37ºC. When the OD_600_ of the *E. coli* donor strain reached 1, we harvested 20 OD_600_· mL units of the culture and washed the cells three times with fresh LB supplemented with DAP. Then, 20 OD_600_ units of wild-type OAE881 cells were harvested, mixed with the washed donor cells, centrifuged, and resuspended in a final volume of 0.5 mL with LB supplemented with DAP. The resuspension was spotted onto 0.45-µm membrane filters (Millipore, United States) and incubated overnight on LB agar plates supplemented with DAP at 30ºC. After 16 h, the conjugation mixture was scraped from the membrane, resuspended in 10 mL LB with 50 µg/mL kanamycin (Km) and plated at different dilutions on LB plates supplemented with 50 µg/mL Km.

Plates were incubated at 30ºC for 48 h to let visible colonies develop. We then pooled the colonies and grew the library in liquid LB supplemented with 50 µg/mL Km for 2 population doublings. We then added glycerol to a final volume of 15%, made multiple 1 mL −80°C freezer stocks (~10^8^ cells/mL) of the final library for subsequent experiments, and collected cell pellets to extract genomic DNA for TnSeq mapping. To map the genomic locations of the transposon insertions and link these insertions to their associated DNA barcodes, we used a variation of the previously described TnSeq protocol [18], where we use a splinkerette adaptor instead of a Y adapter and we used two rounds of PCR to selectively enrich for transposon junctions [89]. We mapped 118,245 barcodes to insertions in the genome.

To construct a mutant library in *Rhodanobacter sp.* FW510-R12, we followed the same conjugation procedure as described above for *Lysobacter soli* OAE881, except that mutants were selected on R2A with 5 µg/mL Km at 25℃. We mapped 478,711 barcodes to insertions in the genome of FW510-R12.

To construct a mutant library in *Rhodanobacter denitrificans* FW104-10B01, we first developed a custom mariner transposon delivery vector, pTKO49_NN1. pTKO49_NN1 was modified from our standard mariner vector (pHLL250_NN1 [90],), to avoid methylated motifs that were identified in the genome of FW104-10B01 from the PacBio sequencing data [91] and which may be targeted by restriction enzymes. These motifs were: GACCAG (removed 1 instance), CTCGAG (removed 1 instance), ATCNNNNNNTGGA (removed 2 instances), TCCANNNNNNGAT (removed 2 instances), GAANNNNNTTGG (removed 2 instances), TTCANNNNNCTC, and GAGNNNNNTGAA, where the last two motifs did not appear in pHLL250_NN1. Compared to pHLL250_NN1, pTKO49_NN1 has eight single nucleotide changes and was made in a two step process, using our previously described parts-based “Magic Pools” approach [87]. First, we used Golden Gate assembly with five parts to make the non-barcoded vector pTKO49. The part1 vector (pTKO2) containing the majority of the mariner transposase gene was generated from gBlock gHL47 (Integrated DNA Technologies) and cloned into the pJW52 holding vector via Gibson assembly as previously described [87]. The part2 vector pHLL216 [87] contains the kanamycin resistance gene promoter. The part3 vector (pTKO1) contains the kanamycin resistance gene and was generated from gBlock gHL48. The part4 vector pTKO22 contains the plasmid backbone, a region for incorporating DNA barcodes, a carbenicillin resistance gene, the colE1 origin of replication, and GFP. pTKO22 is two nucleotides different from the part4 in pHLL215 [87] and the two mutations were introduced by amplifying two parts of pHLL215, one with oAD474 and oAD475 (where the middle of oAD475 introduces the two mutations) and the second with oAD476 and oAD477; fusing these two PCR products via PCR with oAD477 and oAD478; and cloning this final PCR product into pJW52. The part5 vector pJW14 [87] contains the promoter for the transposase. pTKO49 was generated with BbsI-mediated Golden Gate assembly with the 5 part vectors as described [87] and was sequence verified. We then incorporated millions of DNA barcodes into pTKO49 to make pTKO49_NN1 using BsmBI-mediated Golden Gate assembly, as described [87]. We moved the pTKO49_NN1 plasmid library into the *E. coli* conjugation donor WM3064 via electroporation to make the conjugation donor strain AMD1385. Using barcode sequencing, we estimate that AMD1385 has over 7 million barcodes. For primer sequences, see S1C Table; for gBlock sequences, see S1D Table; and for plasmids, see S1E Table.

The mutant library of FW104-10B01 was made by conjugation between recipient wild-type FW104-10B01 and strain AMD1385. An aliquot of the AMD1385 donor was grown overnight in 50 mL of LB supplemented with DAP and carbenicillin. To remove residual carbenicillin, we pelleted (via centrifugation) and washed the AMD1385 cells two times with LB supplemented with DAP only. These washed donor cells were mixed in a 1:1 ratio with wild-type FW104-10B01, and the combined cell mix was pelleted by centrifugation. We resuspended the combined pellet in 1 mL of LB with DAP, spread this cell slurry across 4 LB plates supplemented with DAP, and incubated these plates for 12 hours at 30°C. After conjugation, we harvested the cell mixture by scraping up the cells into 10 mL of R2A supplemented with 15% glycerol, and froze down 10 1-mL aliquots at -80°C. One of these conjugation aliquots was used to estimate the number of mutant colony forming units (CFU) in our conjugation mixture. Briefly, we thawed this tube, performed 10-fold serial dilution in R2A, plated these dilutions on R2A plates supplemented with 10 μg/mL kanamycin, incubated these plates for 4 days at 30°C, and counted the number of CFUs. Based on these numbers, we thawed additional aliquots of the conjugation mixture and plated across 84 R2A plates supplemented with 10 μg/mL kanamycin. After 4 days incubation at 30°C, we scraped up all of the mutant colonies into 10 mL of R2A, measured the OD_600_ of this mixture, and inoculated these cells into 100 mL fresh R2A with 10 μg/mL kanamycin at a starting OD_600_ of 0.25. The cells were grown for 12 hours at 30°C to allow a few population doublings, glycerol was added to a final concentration of 15%, and we made single-use aliquots of the final mutant library (rhodanobacter_10B01_ML12). We also extracted genomic DNA at this point for TnSeq mapping of the transposon insertions. We mapped 507,095 barcodes to insertions in the genome of FW104-10B01.

To construct a mutant library in *Rhodanobacter* sp. FW510-T8, we used conjugation with *E. coli* donor strain AMD1385 carrying pTKO49_NN1. Transconjugants were selected on R2A agar with 10 µg/mL kanamycin at 25°C and pooled to generate the FW510-T8 RB-TnSeq library. The final library was constructed by collecting colonies from approximately 150 selection plates, corresponding to an estimated >500,000 mutants. We mapped 719,225 barcodes to insertions in the genome of FW510-T8.

### RB-TnSeq assays

We performed fitness assays as described previously [18]. Briefly, the mutant library was recovered from the freezer in the rich media that was used to make the library, inoculated at OD_600_ = 0.02 into the condition of interest, grown until saturation, genomic DNA was extracted, and barcodes were amplified with PCR and sequenced using Illumina. Fitness values (log2 ratios) were calculated as described previously [18].

For *Brevundimonas vesicularis*_*C*_ GW460-12-10-14-LB2, we performed experiments in 24 well culture plates with a volume of 2 mL, Breathe Easy seals, at 20℃ or 30℃, and shaken at 200 rpm. We used a defined minimal medium with 20 mM glucose or 0.5% v/v Tween-20 as the carbon source. The basal medium (RCH2_defined_noCarbon) included 0.25 g/L Ammonium chloride, 0.1 g/L Potassium Chloride, 0.6 g/L Sodium phosphate monobasic monohydrate, 30 mM PIPES sesquisodium salt, Wolfe’s mineral mix (ATCC), and Wolfe’s vitamins (ATCC), and was adjusted to pH 7.0 using 2N NaOH.

For *Lysobacter soli* OAE881, we performed fitness assays with 17 defined carbon sources (each at 20 mM) as well as casamino acids at 30℃ and with 200 rpm shaking, again with RCH2_defined_noCarbon as the basal medium. We used 96-well deep well (V bottom) blocks with a 0.8 mL culture volume.

For *Rhodanobacter denitrificans* FW104-10B01, we performed 30 fitness experiments in defined media. Two of these experiments were conducted in a minimal defined medium (RCH2_defined_noCarbon) with 20 mM glucose, at either 20℃ or 30℃; these were grown in 24 well culture plates with a volume of 2mL, Breath Easy seals, and 200 rpm shaking. For the remainder of the experiments, we used 13 different carbon sources, and we added 100 µM of each amino acid to the medium. These were grown in 96-well deep well blocks with a 0.8 mL culture volume and 700 pm shaking. We also performed a variety of stress experiments in a rich medium (R2A).

For *Rhodanobacter sp.* FW510-R12*,* we performed fitness assays in NLDM_defined_nocarbon medium with 20 mM glucose ± 1 mM L-serine at room temperature. (NLDM_defined_nocarbon includes 1 mM ammonium chloride, 3.3 mM potassium chloride, 0.812 mM magnesium sulfate, 0.68 mM calcium chloride, 4.05 mM disodium phosphate, 0.95 mM sodium phosphate monobasic, ATCC Wolfe’s mineral mix, and ATCC Wolfe’s vitamin mix.) Experiments were performed in glass test tubes (10 ml culture volume) and shaken at 200 rpm.

For *Rhodanobacter sp.* FW510-T8, we performed fitness assays in NLDM_defined_nocarbon medium with 20 mM glucose ± 1 mM L-methionine at room temperature. Experiments were performed in 96-well deep well blocks with a culture volume of 1 mL, sealed with Aeraseals, and shaken at 700 rpm.

For *Bacteroides thetaiotaomicron* VPI-5482, fitness assays were performed anaerobically in four different nitrogen sources using Varel Bryant medium (Liu et al., 2021). The basal medium was modified to minimize or eliminate nitrogen by replacing the L-methionine and L-cysteine with either (134 µM L-methionine + 3mM L-cysteine) or (134 µM L-methionine + 3mM dithiothreitol + 3mM sodium sulfide) or (0.1 ng/L cyanocobalamin + 3mM dithiothreitol + 3mM sodium sulfide).

### Cloning of putative biosynthetic genes

Candidate biosynthetic genes were cloned to replace RFP in pBbA2c-RFP (AddGene plasmid 35326; [26]), which contains a p15A origin, CmR for chloramphenicol resistance,the *tetR* repressor, and a *tet*-inducible promoter driving the expression of RFP. As previously described [13], the pBbA2c-RFP vector was linearized by PCR and the target genes’ inserts were either PCR amplified from genomic DNA or ordered as gBlocks (Integrated DNA Technologies). Sequences for primers are in S1C Table; sources of genomic DNA for cloning are listed in S1F Table; and sequences for gBlocks are in S1D Table. After cloning using the Gibson Assembly Mastermix (New England Biolabs), plasmids with the correct sequence were identified by Sanger sequencing (Elim Biopharmaceuticals) and are listed in S1E Table. See S2 Data for plasmid maps and sequences.

### Complementation assays

Many of the complementation assays used strains of *E. coli* from the Keio collection in which a gene of interest (*thrB, glyA, serB, serC,* or *argE*) is replaced with a kanamycin resistance gene [27]. To study the alternative *dapE* from *Echinicola* or *Pedobacter*, we used the AT978 strain of *E. coli* [44]. AT978 was obtained from the Coli Genetic Stock Center and its genotype is LAM- e14- *dapE9 relA1 spoT1 thiE1*.

To construct the *dapF* knockout strain in *E. coli* strain MG1655, we used a modified CRISPR/Cas9 system described previously [92]. Briefly, the recipient strain containing the pEcCas plasmid encoding Cas9 and λ-Red recombinase is transformed by electroporation with the pEcgRNA plasmid carrying a dapF-specific sgRNA and a specific linear DNA template. The specific N20 spacer was predicted using the CCTop website (https://cctop.cos.uni-heidelberg.de/) [93,94].The knockout DNA template was created by combining the upstream and downstream region of *dapF* using overlapping PCR. The transformants were selected on kanamycin and spectinomycin (both at 50 μg/mL). After verification by PCR and sequencing, the positive clones were cured of both plasmids.

For all the strains used in the complementation assays, plasmids were introduced in the appropriate strains by electroporation and transformants were selected on LB supplemented with 20 μg/mL chloramphenicol.

Growth assays were run in 96-well flat-bottom plates with a culture volume of 200 μL in either Tecan Sunrise or Agilent Epoch2 plate readers. The temperature ranged from 27℃ to 30℃ and the shaking speed from ~300–550 rpm depending on the instrument. All strains for complementation assays (Table 5) were grown in minimal M9 medium with 20 mM glucose and 20 µg/mL chloramphenicol to maintain the plasmid. We sometimes added 4 nM of anhydrotetracycline (aTc) as an inducer. We found that aTc at this concentration moderately inhibited the growth of *E. coli* carrying the control plasmid (which expresses RFP), and in general, strains complemented with the *E. coli* gene grew better without aTc, but aTc was often needed for good growth of strains with heterologous complementation.

**Table 5:** Strains for complementation assays.

Strain	Background	Plasmid	Description
AMD806	BW25113	pBbA2c-RFP	wild-type with RFP expressed from pBbA2c
AMD3424	BW25113 *thrB::ka*n	pBbA2c-RFP	*ΔthrB* with pBbA2c-RFP
AMD3425	BW25113 *thrB::kan*	pALF4	*ΔthrB* with BT2402 cloned into pBbA2c
AMD3426	BW25113 *thrB::ka*n	pALF5	*ΔthrB* with DET_RS08350 cloned into pBbA2c
AMD3427	BW25113 *thrB::ka*n	pALF17	*ΔthrB* with *E. coli thrB* cloned into pBbA2c
AMD3428	BW25113 *glyA::kan*	pBbA2c-RFP	*ΔglyA* with pBbA2c-RFP
AMD3429	BW25113 *glyA::kan*	pALF1	*ΔglyA* with BBR_RS12915-BBR_RS12920 cloned into pBbA2c
AMD3431	BW25113 *glyA::kan*	pALF15	*ΔglyA* with *E. coli glyA* cloned into pBbA2c
AMD3432	BW25113 *serB::kan*	pBbA2c-RFP	*ΔserB* with pBbA2c-RFP
AMD3433	BW25113 *serB::kan*	pALF3	*ΔserB* with CBO1128 cloned into pBbA2c
AMD3434	BW25113 *serB::kan*	pALF16	*ΔserB* with *E. coli serB* cloned into pBbA2c
AMD3451	BW25113 *serC::kan*	pBbA2c-RFP	*ΔserC* with pBbA2c-RFP
AMD3452	BW25113 *serC::kan*	pALF7	*ΔserC* with CBO1126 cloned into pBbA2c
AMD3453	BW25113 *serC::kan*	pALF8	*ΔserC* with A0A843E9R6 cloned into pBbA2c
AMD3454	BW25113 *serC::kan*	pALF9	*ΔserC* with A0A1T4W7T3 cloned into pBbA2c
AMD3455	BW25113 *serC::kan*	pALF19	*ΔserC* with *E. coli serC* cloned into pBbA2c
AMD3456	BW25113 *argE::kan*	pBbA2c-RFP	*ΔargE* with pBbA2c-RFP
AMD3457	BW25113 *argE::kan*	pALF6	*ΔargE* with W3Y6L2 cloned into pBbA2c
AMD3458	BW25113 *argE::kan*	pALF18	*ΔargE* with *E. coli argE* cloned into pBbA2c
AMD3644	MG1655	pBbA2c-RFP	wild-type with RFP expressed from pBbA2c
AMD3442	MG1655 *ΔdapF*	pBbA2c-RFP	*ΔdapF* with RFP expressed from pBbA2c
AMD3443	MG1655 *ΔdapF*	pALF11	*ΔdapF* with HMPREF2922_03580 cloned into pBbA2c
AMD3444	MG1655 *ΔdapF*	pALF21	*ΔdapF* with *E. coli dapF* cloned into pJB00059
AMD3914	AT978	pBbA2c-RFP	AT978 *(dapE9*) with RFP expressed from pBbA2c
AMD3917	AT978	pALF23	AT978 with *E. coli dapE* cloned into pBbA2c
AMD3916	AT978	pALF14	AT978 with Echvi_1427 cloned into pBbA2c
AMD3915	AT978	pALF13	AT978 with CA265_RS23900 cloned into pBbA2c

For W3Y6L2 from *Veillonella denticariosi* cloned into an *argE::kan* strain of *E. coli*, our standard aerobic assay did not yield any growth. We were unsure about the metal cofactor of W3Y6L2, and M9 medium does not contain any added metals except magnesium. (We used M9 salts from BD Difco (provided at 5x), which contains 33.9 g/L anhydrous disodium phosphate; sodium phosphate often contains impurities of iron and other transition metals, which are required for the growth of wild-type *E. coli* [95].) We tried adding Wolfe’s minerals, but the strain complemented with W3Y6L2 still did not grow aerobically. For anaerobic assays, we used a plate reader housed in a Coy anaerobic chamber (90% N_2_, 5% CO_2_ and 2.5% H_2_ atmosphere) with no shaking.

### LC/MS analysis of diaminopimelate secretion

Various strains of *E. coli* were grown in M9 glucose medium containing 20 µg/mL chloramphenicol, with or without 4 nM of aTc, for 16 hours at 30℃. Cultures were centrifuged at 7,000 rpm to remove cells and supernatants were filtered (0.22um PVDF), then lyophilized until dry. Dried materials were stored at -80℃ until just prior to analysis, and then resuspended in 150µL of 100% methanol containing internal standards (S1 Data). Samples were vortexed 2x10 seconds, sonicated in an ice water bath for 15 minutes, centrifuged (10,000 rcf, 5 min, 10℃), filtered (0.22um PVDF) and then filtrates were transferred to amber glass vials for LC-MS/MS analysis. Metabolites were separated using hydrophilic interaction liquid chromatography (HILIC) for polar metabolomics. Analyses were performed using an InfinityLab Poroshell 120 HILIC-Z column (Agilent) on an Agilent 1290 HPLC connected to a Q Exactive hybrid quadrupole-Orbitrap mass spectrometer (Thermo Fisher Scientific) using ElectroSpray Ionization (ESI). LC-MS/MS methods were previously described [96]; run specific parameters are provided in S1 Data. Peak heights and MS2 were extracted using the RaMS 1.4.3 package [97] in R4.5.1; intensities and annotation evidence are provided in S1 Data; extracted ion chromatograms for selected samples are in Fig 8A and for all samples are in Fig 10; the MS2 mirror plot is in Fig 8B.

**Fig 10:**
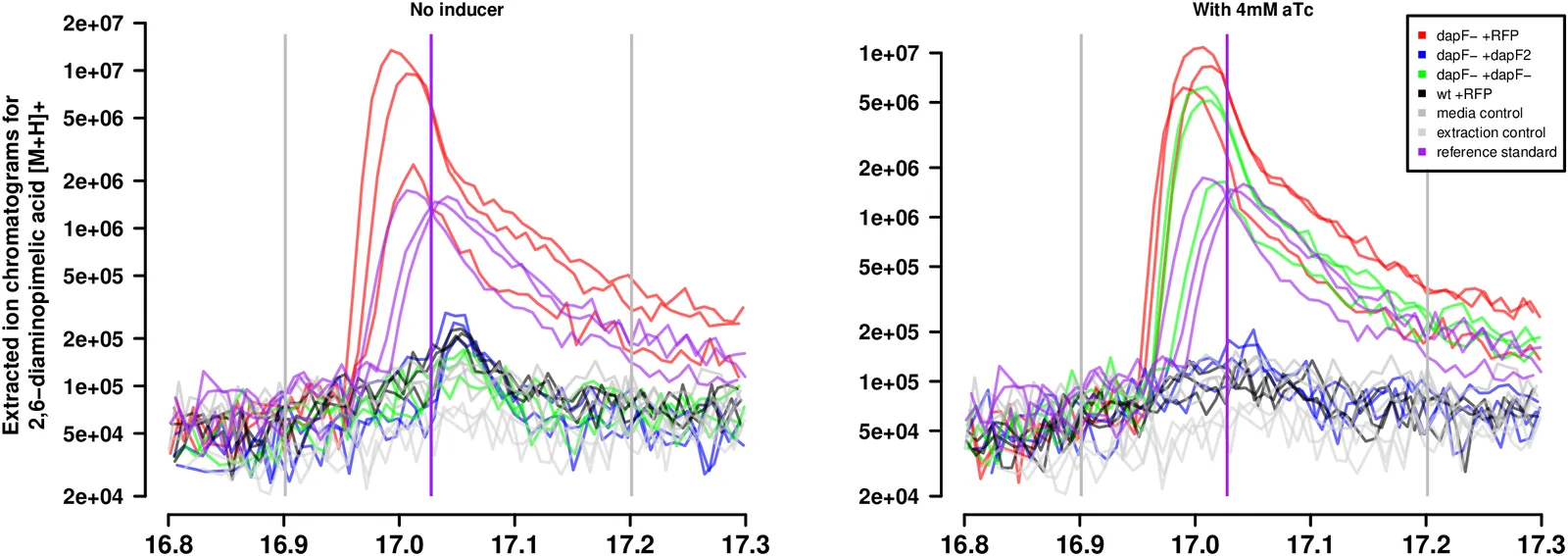
LC/MS extracted ion chromatograms for the [M + H]+ adduct of 2,6-diaminopimelic acid. The left panel shows supernatants from strains grown without added inducer, and the right panel shows supernatants from strains grown with 4 nM aTc. The vertical purple line shows the average peak retention time for the standard (across 3 replicates). The vertical grey lines show the range of retention times used for integration (in Fig 8C & 8D).

## Supporting information

S1 Text Additional rationales for gene functions. (A) Roles of *serB* and *serBK* in *Bacteroides thetaiotaomicron*. (B) Rationale for a putative alternative N-succinyl-L-glutamate synthase. (C) Homoserine phosphotransferase HomK. (D) Rationales for other previously-proposed alternate enzymes. (E) Diverged enzymes with experimental evidence. (F) Highly-diverged regulatory subunits of acetolactate synthase.
(PDF)

S1 Table Supplementary tables S1A-S1F. (A) Locus tags for methionine synthesis in Xanthomonadales. (B) Functional residues in predicted diverged enzymes. (C) Primers. (D) gBlocks. (E) Plasmids. (F) Base strains for complementation assays and strains used as sources of DNA.
(XLSX)

S1 Data LC/MS data for diaminopimelate secretion. (XLSX)

S2 Data Plasmid maps in Genbank and snapgene formats. (ZIP)

S3 Data Growth curves from complementation assays. (ZIP)
