# Supplementary material for: Novel enzymes for amino acid biosynthesis in diverse bacteria and archaea: GapMind 2026
Source: PLoS Genet. 2026 Nov 12;22(11):e1012372. doi: 10.1371/journal.pgen.1012372 (PMC13759103; doi:10.1371/journal.pgen.1012372)
Supplement: Supplementary file 1 [file pgen.1012372.s001.pdf]

Supporting Text for “Novel enzymes for amino acid biosynthesis in diverse bacteria and archaea: GapMind 2026”

Morgan N. Price[1], Valentine V. Trotter[1], Markus de Raad[1], Suzanne M. Kosina[1], Hira P. Lesea[1], Anthony L. Shiver[2], Leslie A. Day[3], Surya Tripathi[1], Marta Torres[1], Trenton K. Owens[1], Audrey Liwen Wang[1], Anna Lisa Fear[1], Xuanyu Tao[4], Jizhong Zhou[4], Adam M. Deutschbauer[1], and Adam P. Arkin[1]

1 Environmental Genomics and Systems Biology, Lawrence Berkeley National Lab, Berkeley, CA, USA
2 Department of Bioengineering, Stanford University, Stanford, CA, USA
3 Geisel School of Medicine, Dartmouth College, Hanover, NH, USA
4 Institute for Environmental Genomics, School of Biological Sciences, University of Oklahoma, Norman, OK, USA

S1A Text: Roles of *serB* and *serBK* in *Bacteroides thetaiotaomicron*

As part of a study of nitrogen source utilization by *Bacteroides thetaiotaomicron* VPI-5482, we collected fitness data with three amino acids as the sole sources of nitrogen: L-serine, L-asparagine, and L-glutamine. We found that *serBK* from *B. thetaiotaomicron* (BT1151; DUF1015 family) was important for fitness when L-serine was the nitrogen source (Figure S1). In previously-published experiments (Liu et al. 2021), *serBK* was also somewhat important for the utilization of D-serine, which is not explained.

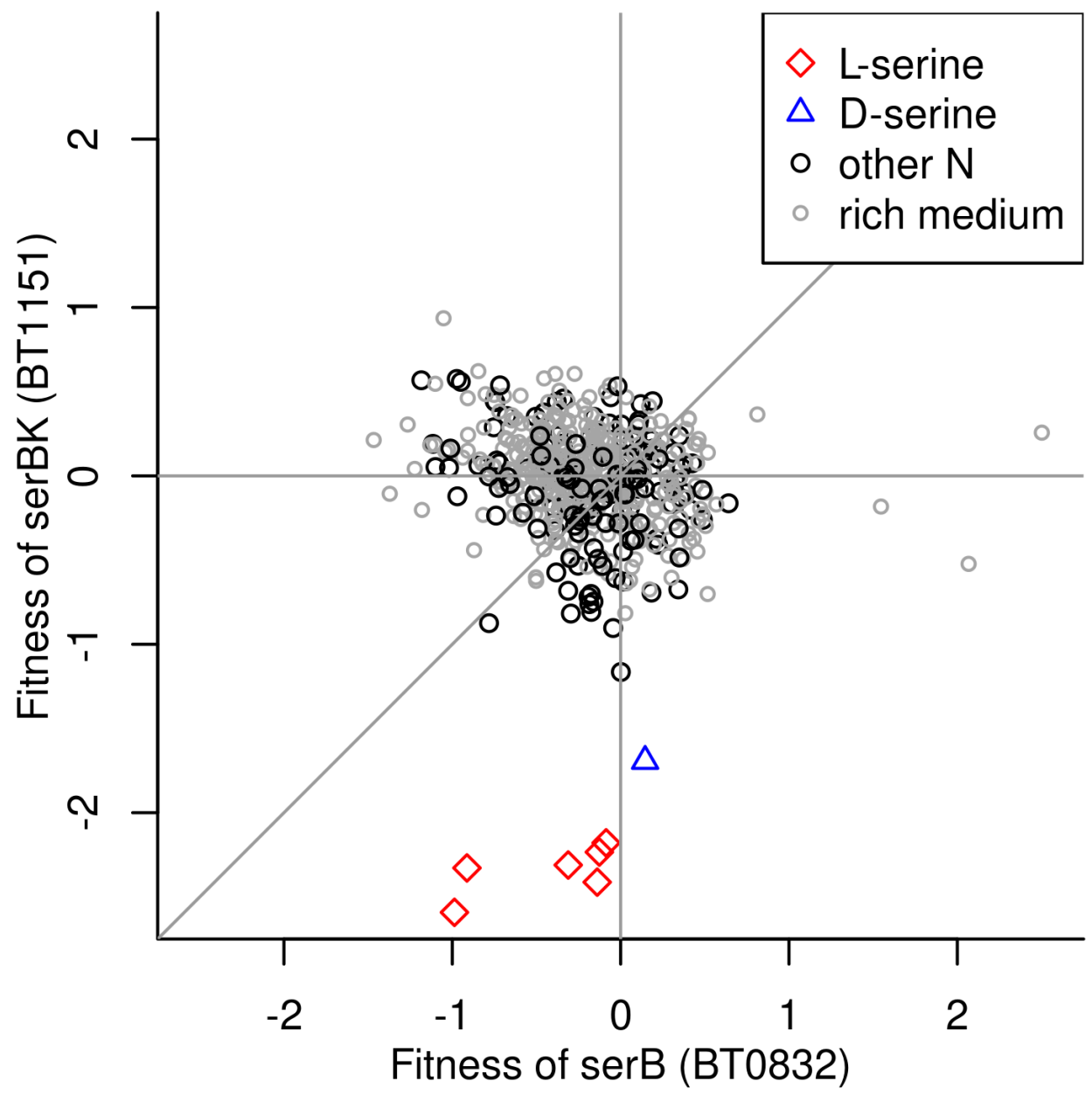

Figure S1: Fitness of serB and serBK in *Bacteroides thetaiotaomicron*. We highlight experiments with L-serine or D-serine as the sole source of nitrogen. Experiments with a defined medium with another nitrogen source (usually ammonium) are in black and experiments with a rich medium are in grey. Except for the new nitrogen source data, the data is from (Liu et al. 2021) and mouse experiments are not shown.

In most defined media conditions, neither *serB* (BT0832) nor *serBK* was important for fitness (Figure S1). It appears that either gene suffices for converting O-phospho-L-serine to L-serine.

S1B Text: Rationale for a putative alternative N-succinyl-L-glutamate synthase

Steroidobacter denitrificans DSM 18526 was reported to grow in defined minimal medium ((Fahrbach et al. 2008); DSMZ medium 1116), but its pathway for synthesizing arginine appears to be incomplete. In particular, from its genome, we could not identify any proteins with similarity to characterized N-acyl-L-glutamate synthases (ArgA). However, its genome does encode a cluster of genes for arginine synthesis, which includes a hypothetical protein (ACG33_RS14135; UniProt A0A127FCT3). Homologs of this hypothetical protein are virtually always found in arginine synthesis operons (Figure S2), which suggests a role in arginine synthesis. *S. denitrificans* is also missing the expected N-acylcitrulline hydrolase (ArgE), but some homologs of ACG33_RS14135 are found near plausible candidates for *argE* (purple genes in Figure S2). So we propose that ACG33_RS14135 is the missing ArgA.

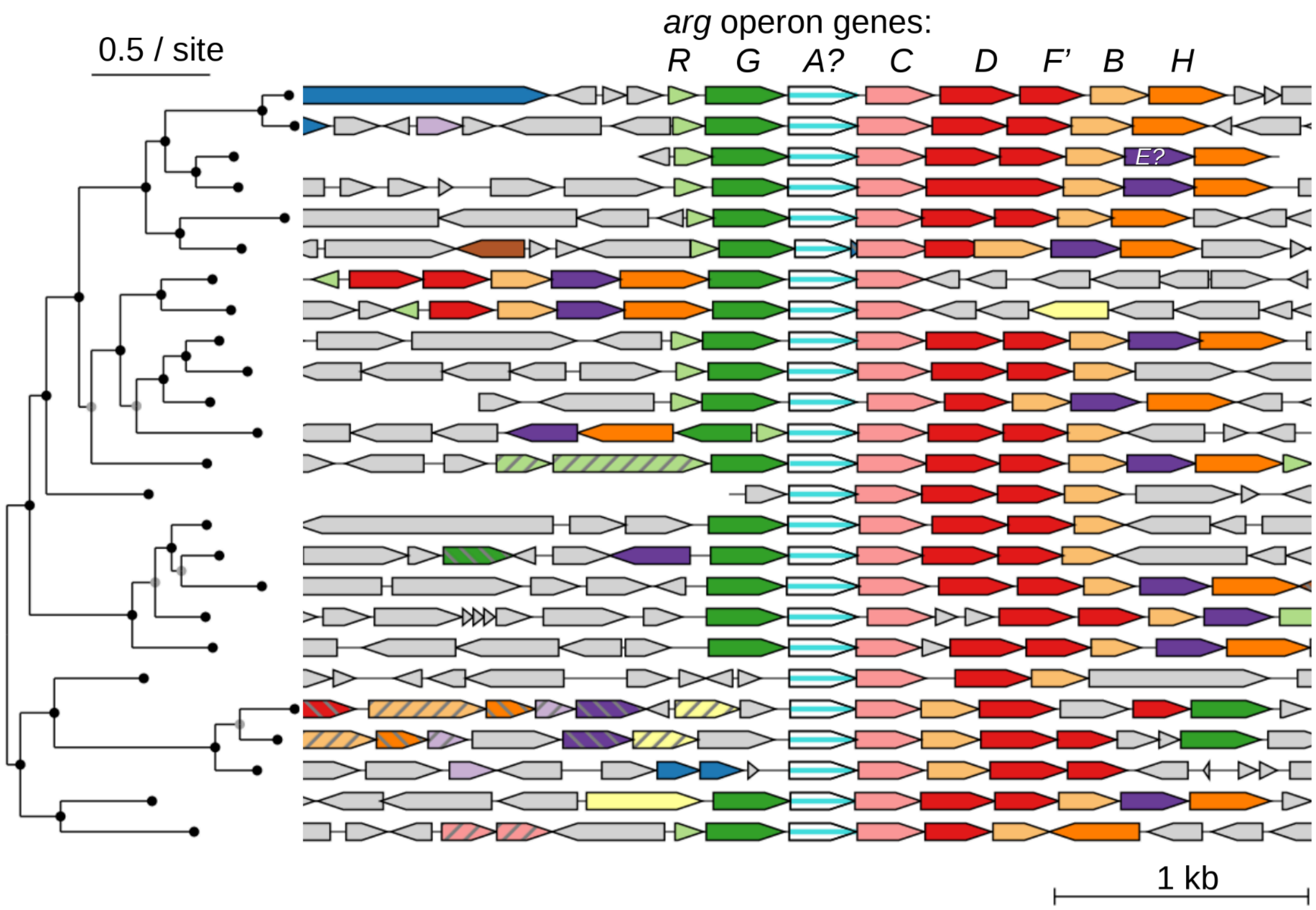

Figure S2: Gene neighbors for a putative alternate N-succinylglutamate synthase. Each row shows a homolog of the putative alternate *argA* (central column) from a different genus, along with its nearby genes. Other protein-coding genes are color-coded by sequence similarity. This figure was created using *fast.genomics* (Price and Arkin 2024) and the central column includes 25 random proteins whose similarity to ACG33_RS14135 is at least 30% of the maximum bit score. The phylogenetic tree (at left) is based on the protein sequences of its homologs. Besides the proposed alternate *argA* (labeled *A?*), the genes labeled are: N-acylglutamate kinase *argB*; N-acyl-γ-glutamyl phosphate reductase *argC*; N-acylornithine aminotransferase *argD*; N-succinylornithine transcarbamoylase *argF*'; N-acylcitrulline hydrolase *argE* (in some of the clusters, in purple); argininosuccinate synthetase *argG*; argininosuccinate lyase *argH*; and transcriptional regulator *argR*.

If ACG33_RS14135 is replacing the usual ArgA, then homologs of both proteins should not be found in the same genome. We compared the distribution of homologs of ACG33_RS14135 to that of homologs of ArgA from *Pseudomonas aeruginosa* (AT700_RS27105), using *fast.genomics* (Price and Arkin 2024). Potential functional orthologs (above 25% of the maximum bit score) were never found together in the same genome. (*Steroidobacter argA* was found in 109 genera and *Pseudomonas argA* was found in 970 genera.)

Using Foldseek (van Kempen et al. 2024), we found weak similarity between the predicted structure of ACG33_RS14135 (UniProt A0A127FCT3) and the experimentally-determined structure of a D-glutamate acetyltransferase ((Yu et al. 2023); PDB:7XRJ). Although the

structural similarity is remote (root mean square deviation = 5.6 Å, e-value = $1.4 \cdot 10^{-7}$), it supports our proposal.

Although most bacteria synthesize arginine via N-acetylated intermediates, some bacteria use N-succinylated intermediates (Shi et al. 2006; Luque 2010). A single residue can determine whether the N-acylornithine transcarbamoylase (ArgF') acts on N-succinylornithine or N-acetylornithine: mutating position 92 of an N-acetylornithine carbamoyltransferase from glutamate to alanine, proline, serine, or valine switches its substrate specificity to N-succinylornithine (Shi et al. 2007). Among 70 ArgF' proteins encoded near ACG33_RS14135 or its homologs (from the *fast.genomics* database), 90% have A, P, S, or V at this position. If we also allow the chemically similar residues isoleucine or threonine, the proportion rises to 97%. (The two remaining homologs have a glutamine at this position.) So it appears that these organisms synthesize arginine via N-succinylated intermediates, and hence that these putative ArgA enzymes form N-succinylglutamate, not N-acetylglutamate. ACG33_RS14135 is included in the revised GapMind as a predicted ArgA.

S1C Text: Homoserine phosphotransferase HomK

As discussed in the main text, BT2402 is an alternative to homoserine kinase (HomK) that is required for threonine synthesis in *Bacteroides thetaiotaomicron* (Price et al. 2020). BT2402 rescued the growth of a *thrB::kan* strain of *E. coli* in minimal medium (Figure S3A), which confirms that BT2402 and related proteins (also known as TIGR02535 (Haft et al. 2013) or ThrB2 (Ashniev et al. 2022)) form O-phosphohomoserine. We named these proteins HomK. Close homologs of BT2402 (over 80% identical) from two species of a related genus, *Phocaeicola,* also seem to be involved in threonine synthesis. Specifically, both homologs have similar fitness patterns as threonine synthase, with cofitness = 0.93 in *P. dorei* CL03T12C01 (see Fitness Browser) and cofitness = 0.79 in *P. vulgatus* CL09T03C04 (data of (Huang et al. 2024)).

By analyzing the predicted structure of BT2402, we identified a likely specificity-determining residue that distinguishes HomK from homologs that are cofactor-independent phosphoglycerate mutases. Specifically, we identified the catalytic serine in BT2402 (S56) by aligning it to the phosphoglycerate mutase SSO0417 (Potters et al. 2003). This phosphorylated serine is thought to be an intermediate in the transfer of phosphate between substrates. When we folded BT2402 with phosphorylated S56 together with L-homoserine and two Mg^{2+} ions using Boltz-2 (Passaro et al. 2025), the phosphate group was placed near the hydroxyl group of homoserine, and the carboxylate side chain of a glutamate residue (E299) was just 2.8 Å from the amino group of homoserine (Figure S3B). As shown in Figure S3C, this glutamate is conserved in putative HomK proteins, while phosphoglycerate mutases almost always have a lysine at this position (consistent with a preference for negatively charged substrates).

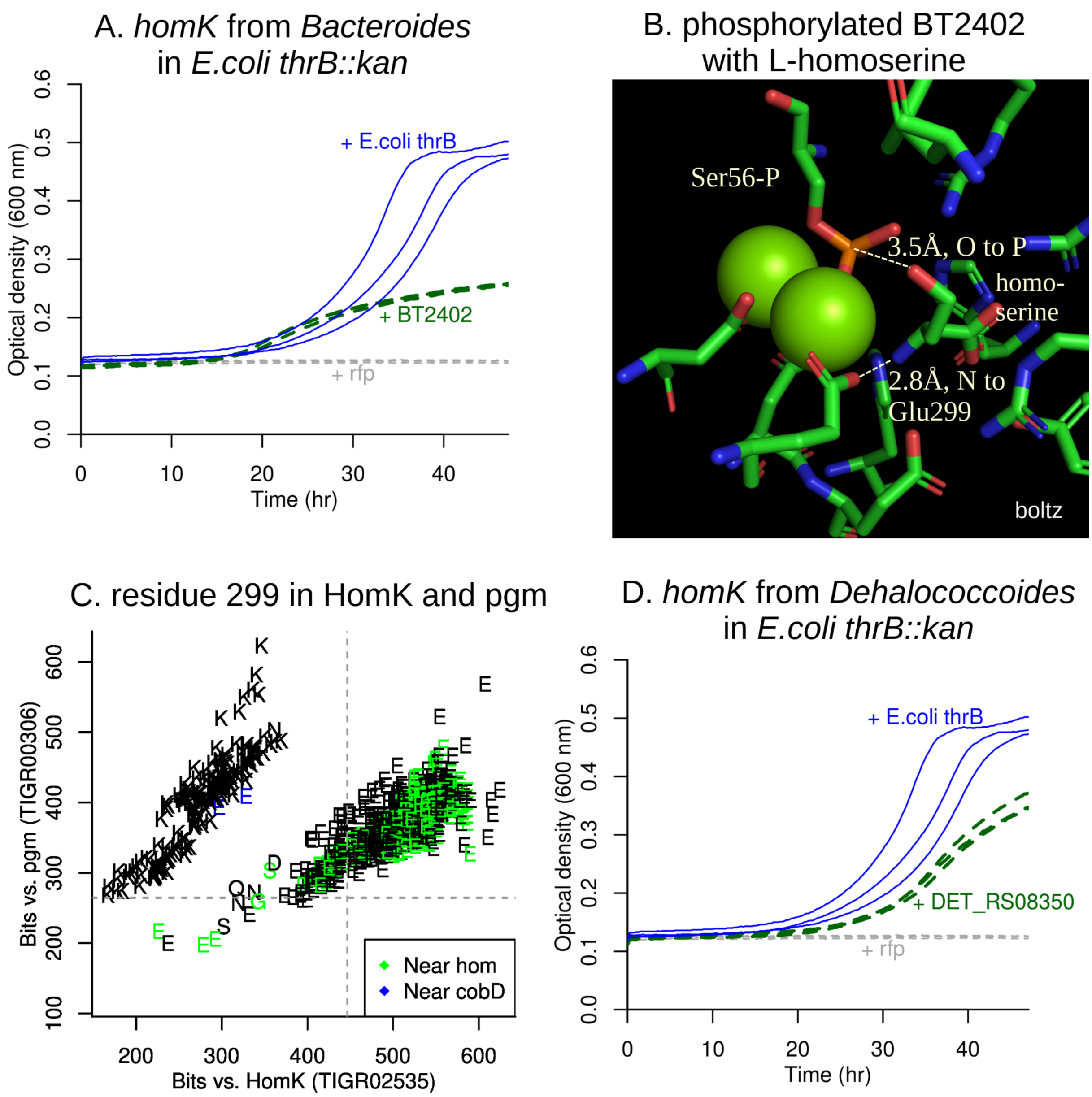

Figure S3: A putative specificity-determining residue differentiates HomK from cofactor-independent phosphoglycerate mutases. (A) Growth of a *thrB::kan* strain of *E. coli* in minimal M9 medium is complemented by BT2402. (B) Predicted model of BT2402 (HomK from *B. thetaiotaomicron*), with its catalytic serine 56 phosphorylated, bound to two Mg^{2+} ions (green spheres) and L-homoserine. (C) The putative specificity-determining residue (E299 in BT2402) across 1,012 homologs of BT2402 (30% amino acid identity or above and at least 300 a.a. aligning) from representatives of different genera (from *fast.genomics*). The *x* and *y* axes show how similar each protein is to the hidden Markov models for HomK (TIGR02535) or cofactor-independent phosphoglycerate mutases (TIGR00306) from TIGRFams (Haft et al. 2013), and the dotted lines show the "trusted" bit score cutoffs from TIGRFams. The plotting symbols show the specificity-determining residue. Colors highlight homologs that are encoded near and on the same strand as either homoserine dehydrogenase (*hom*), which forms the

substrate of HomK, or *cobD*. 13 homologs that lack the catalytic serine or threonine (corresponding to Ser56 in BT2402) were omitted. (D) Growth of a thrB::kan strain of E. coli in minimal M9 medium is complemented by DET_RS08350, a diverged HomK protein from *Dehalococcoides mccartyi*. In (A) and (D), each strain has three technical replicates, and 4 nM anhydrotetracycline was added as an inducer.

We did find two homologs of HomK that are more similar to the TIGRFam model for cofactor-independent phosphoglycerate mutases than to the model for HomK, yet have a glutamate aligning to E299. These are FWH37_03765 and G4O51_00460, both from metagenome-assembled genomes of Bathycorpusculaceae. These genes are encoded adjacent to L-threonine O-3-phosphate decarboxylase (*cobD)*, which is part of cobalamin synthesis. We speculate that FWH37_03765 and G4O51_00460 form L-threonine O-3-phosphate.

Among the homologs of BT2402 that are encoded near homoserine dehydrogenase and have conserved E299, we annotated several diverged homologs (41% identity or below) in GapMind. We verified that these homologs have conserved the other functional residues, namely the catalytic serine and the metal-coordinating residues. Cofactor-independent phosphoglycerate mutases rely on divalent metal cations for activity (van der Oost et al. 2002; Potters et al. 2003). The experimental structure of the enzyme from *Pyrococcus horikoshii* (PDB:2zkt) shows two zinc ions, while the enzymes from *Sulfolobus* or *Pyrococcus* prefer magnesium, cobalt, or manganese. The coordinating residues in BT2402 would be D9, S56 (the catalytic serine), D346, and H347 for one metal ion and D274, H278, and H325 for the other. One of the diverged HomK proteins that we identified is DET_RS08350, which was confirmed by a complementation assay (Figure S3D). The other diverged HomK proteins that we identified (UniProt:A0A2V1IX31, DCC48_11680, and UniProt:T2GDP6) are 33-41% identical to BT2402 or DET_RS08350 and are included as predictions in the revised GapMind.

What is the phosphate donor for HomK? Based on the homology to phosphoglycerate mutases, the most obvious donor would be 2- or 3-phosphoglycerate. In this case, the products of HomK would be O-phosphohomoserine and glycerate, and so we would expect that a glycerate kinase would be needed for the glycerate to reenter metabolism. We used AnnoTree (Mendler et al. 2019) to test the hypothesis that HomK (TIGR02535) co-occurs with glycerate kinases (KEGG ortholog groups K11529, K00865, or K15918). We found that only 53% of genomes that encode HomK encode one of the known forms of glycerate kinase. For comparison, 67% of genomes that encode that standard form of homoserine kinase (K00872; ThrB in *E. coli*) encode glycerate kinase. The absence of glycerate kinase from so many of the genomes with HomK suggests that phosphoglycerate is not the phosphate donor for HomK. Based on the putative specificity-determining residue E299, which should select for positively-charged substrates, we speculate that the donor is an amino acid such as phosphoserine. In fact, a phosphoserine:homoserine phosphotransferase (ThrH, not homologous to HomK) is able to substitute for ThrB in *Pseudomonas aeruginosa* (Singh et al. 2004).

S1D Text: Rationales for other previously-proposed alternate enzymes

The revised GapMind includes the thiocarboxylate pathway for converting O-phospho-homoserine to homocysteine, which was recently described in *Streptomyces albulus*$_C$ (Hasebe et al. 2024). The new steps are: sulfur carrier protein MetO; [MetO sulfur-carrier protein]-L-cysteine hydrolase Mec, which is required for the maturation of the N terminus of MetO; [MetO sulfur carrier protein]--sulfur ligase MoeZ; and MetO-thiocarboxylate-dependent L-homocysteine synthase MetM. Note that MetM is homologous to threonine synthases and is often misannotated as such.

Methanocaldococcus jannaschii synthesizes proline from serine via ornithine cyclodeaminase, but the protein responsible had not been identified (Graupner and White 2001). A recent study identified a novel family of ornithine cyclodeaminases in *Anabaena* and in *Methanococcus maripaludis* (Burnat et al. 2019). This family is present in *M. jannaschii* as well. Furthermore, the enzyme from *M. maripaludis* (UniProt Q6LXX7) is important for fitness unless proline is provided (data of (Day et al. 2024)). The revised GapMind includes these ornithine cyclodeaminases.

Ashniev and colleagues associated a family of putative transaminases with serine synthesis, via genome context, and named this family SerC2 (Ashniev et al. 2022). A mutant of SAUSA300_1669, which is a member of this family, is a serine auxotroph (Verstraete et al. 2018). This confirms that SerC2 is a family of phosphoserine transaminases.

As discussed in the main text, Ashniev and colleagues associated a family of putative transaminases with serine synthesis, which they named SerC3 (Ashniev et al. 2022). We cloned the representative of this family from *Clostridium*$_F$ *botulinum* (CBO1126, UniProt A5I0W7) into a *serC::kan* strain of E. coli (Baba et al. 2006) and found that it rescued growth in minimal medium (Figure S4A). We also identified a potential phosphoserine aminotransferase in *Paucidesulfovibrio gracilis* DSM 16080 (UniProt A0A1T4W7T3) that is 30% identical to the *serC3* from *C. botulinum*; *P. gracilis* grows in minimal defined medium and this gene would explain how it can make serine. While investigating this family, we also noticed a diverged homolog from a metagenome-assembled genome of a Methanomethylophilaceae sp. (UniProt A0A843E9R6, 38% identical to SerC3 from *C. botulinum*) that is encoded upstream of *serA*. The SerC3 homologs from *P. gracilis* and from Methanomethylophilaceae also rescued the growth of *serC::kan E.coli* (Figure S4B and S4C). These results confirm that SerC3 proteins can form phosphoserine.

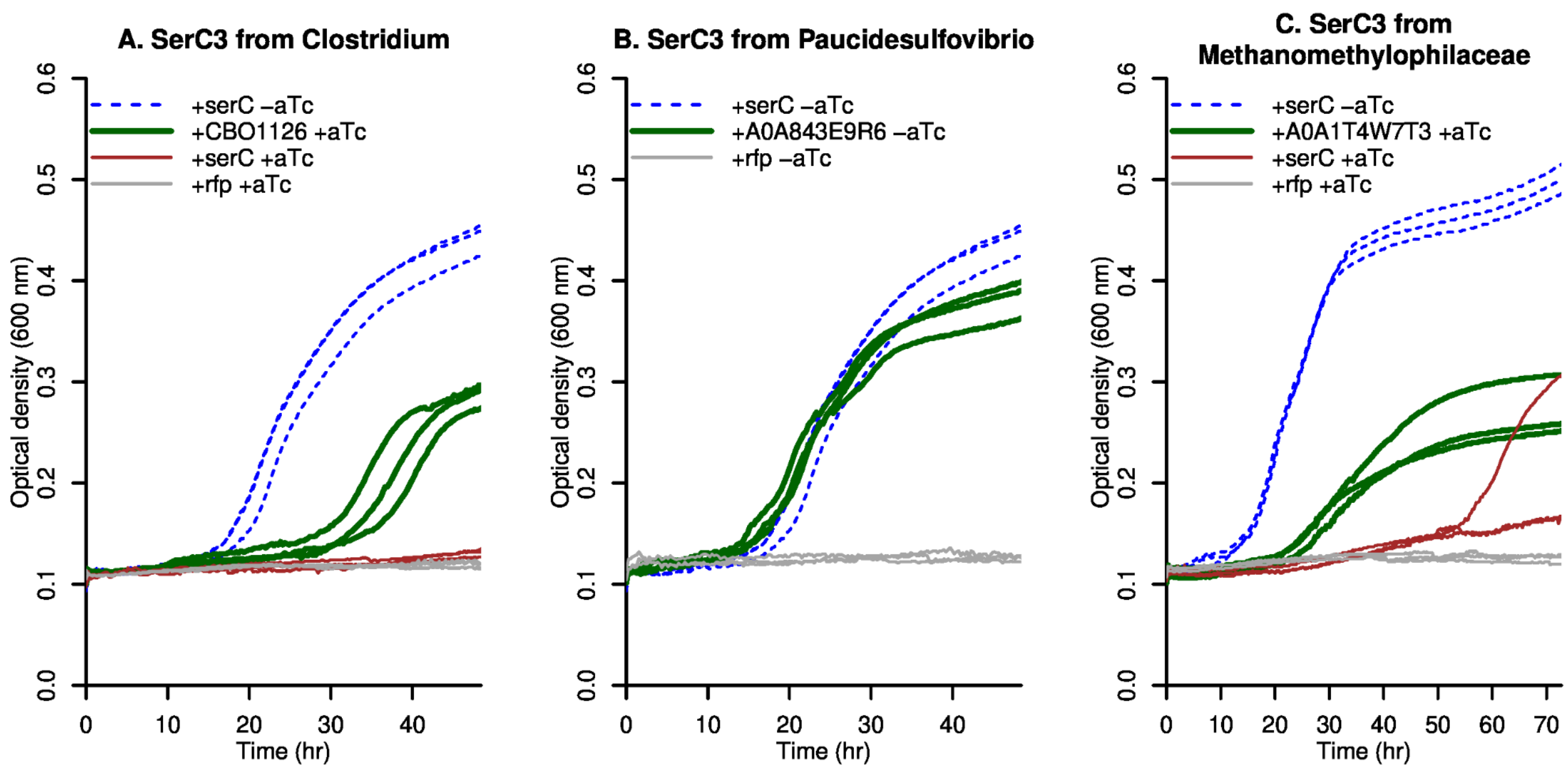

Figure S4: Complementation assays for *serC3*. Each panel shows growth curves, in minimal M9 glucose medium, for *E. coli serC::kan* carrying plasmids that express the indicated proteins. ("+serC" means that *E. coli serC* was on the plasmid; *rfp* is a negative control.) For some wells, 4 nM aTc was added as an inducer. For each plasmid and condition, three technical replicates are shown. Panels (A) and (B) are from the same experiment and the "+serC -aTc" data is the same.

We previously proposed several novel families of methionine synthases, including split MetE (cobalamin-independent synthase) and corrinoid-protein-dependent methionine synthase MesC (Price et al. 2021).

S1E Text: Diverged enzymes with experimental evidence

Eight of these enzymes were previously published. HisN in *Bifidobacterium breve* was recently identified by using RB-TnSeq (Shiver et al. 2025). TK0276 from *Thermococcus kodakarensis* is LysZ (LysW-glutamate kinase and LysW-2- aminoadipate 6-kinase) (Yoshida et al. 2016). Another six enzymes were confirmed using large-scale complementation assays (Biggs et al. 2024).

Seven enzymes were identified using fitness assays. First, two diverged enzymes in *Brevundimonas vesicularis*$_C$ GW460-12-10-14-LB2 were confirmed by conducting RB-TnSeq assays in minimal medium (see Methods): A4249_RS00005 is a diverged homoserine dehydrogenase (fitness = -4.3 to -5.5 in four experiments in defined media) and A4249_RS06835 is a diverged prephenate dehydratase (fitness = -3.7 to -4.5 in four experiments in defined media).

Second, for the missing phosphoserine phosphatase (SerB) from *Lysobacter soli* OAE881 or *Xanthomonas campestris* 8004, we identified proteins that are similar to the biochemically

characterized SerB from *Synechocystis* sp. PCC 6803 (Klemke et al. 2015). The genes from *L. soli* and *X. campestris* are in conserved operons with *serB* and both appear, based on RB-TnSeq data, to be essential ((Luneau et al. 2022); see Methods). These genes must encode the missing *serB*.

Third, acetolactate synthase / acetohydroxybutanoate synthase (AHAS) is a bifunctional enzyme that is involved in the biosynthesis of both valine and isoleucine. AHAS has a catalytic subunit and a regulatory subunit; the regulatory subunit is not strictly required for activity, but the catalytic subunit has far less activity on its own (Vyazmensky et al. 2009). The regulatory subunit (IlvH) usually has two domains, an ACT domain and an ACT-like domain, but some strains of *E. coli* encode an isozyme of AHAS whose regulatory subunit (IlvM) has a single ACT domain (Vyazmensky et al. 2009).

We noticed that some genera of Xanthomonadales that grow in minimal media lack the two-domain form of the regulatory subunit. Instead, we found proteins that were encoded near the catalytic domain but have only a single ACT domain, and are distantly related to *E.coli* IlvM (27-30% identity). To test the roles of these proteins, we used RB-TnSeq data for *Rhodanobacter denitrificans* FW104-10B01, *Lysobacter soli* OAE881, and *Xanthomonas campestris* pathovar *campestris* str. 8004 ((Luneau et al. 2022); see Methods). We found that in all three cases, mutants of the short *ilvM*-like gene (LRK54_RS10305, ABIE51_RS17555, or Xcc-8004.1058.1) had similar phenotypes as mutants in the adjacently-encoded catalytic subunit (Figure S5). This confirms that these are regulatory subunits.

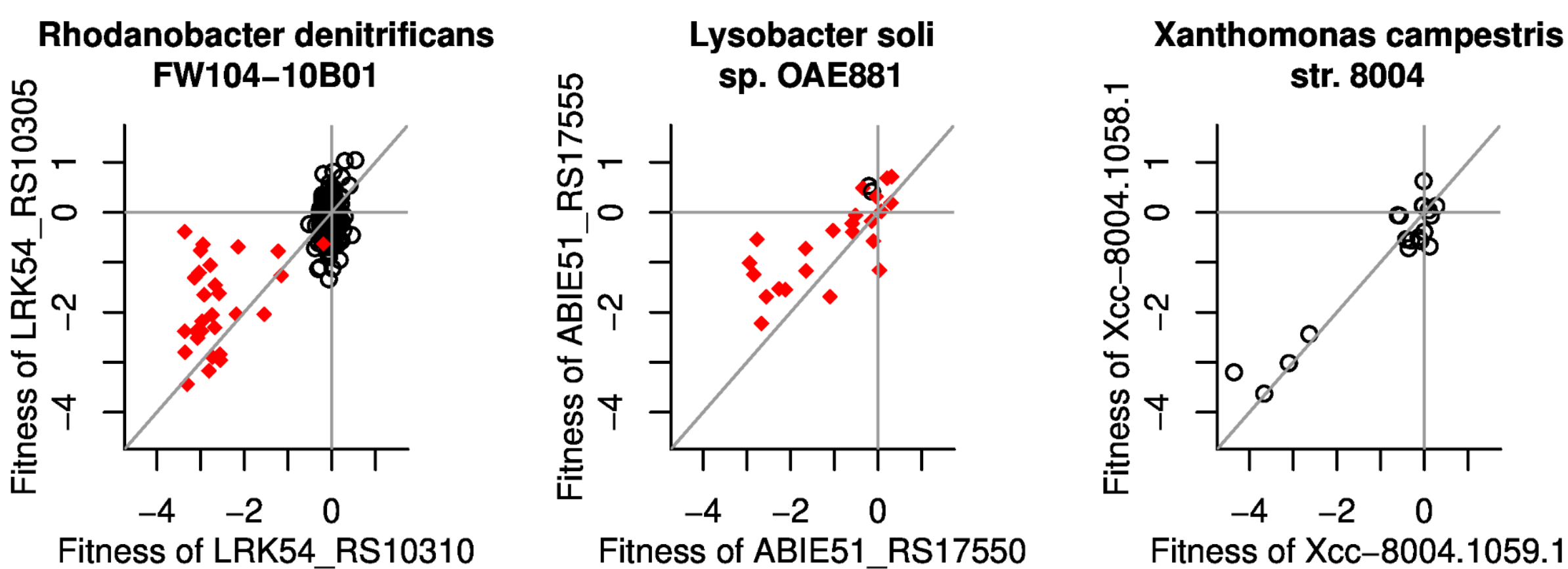

Figure S5: The putative short regulatory subunit of acetolactate synthase is cofit with the catalytic subunit in three different genera of Xanthomonadales. Fitness values (log2 ratios) are shown for the catalytic subunit (*x* axis) and the putative regulatory subunit (*y* axis). For *L. soli*, experiments in a defined minimal medium are highlighted in red. For *R. denitrificans*, experiments in a defined medium are highlighted; for many of these experiments, 100 µM of each amino acid was added. In each panel, the lines show $x = 0$, $y = 0$, and $x = y$.

Three additional enzymes were confirmed using complementation assays, see *homK* from *Dehalococcoides mccartyi* (S1C Text) and *serC3* from *Paucidesulfovibrio gracilis* and from an uncultured *Methanomethylophilaceae* (S1D Text).

S1F Text: Highly-diverged regulatory subunits of acetolactate synthase

We noticed that the regulatory subunit for acetolactate / acetohydroxybutanoate synthase appeared to be missing from many Thermoproteota, including from *Pyrolobus fumarii*, which is prototrophic for amino acids (Blöchl et al. 1997). In many Thermoproteota, we found an ACT domain protein conserved near the catalytic subunit. For instance, KCR_RS03285 from *Korarchaeum cryptofilum* has an ACT domain and is encoded adjacent to the catalytic subunit (KCR_RS03290). When we used the AlphaFold 3 server (Abramson et al. 2024) to predict a protein complex between the two putative subunits from *K. cryptofilum*, it reported a high-confidence interaction (ipTM, the predicted template modeling score for the interface, was 0.83). Furthermore, the predicted interaction is similar to the experimentally determined interaction of the AHAS subunits from yeast (Figure S6). So, in the revised GapMind, KCR_RS03285 is included as a predicted regulatory subunit of AHAS.

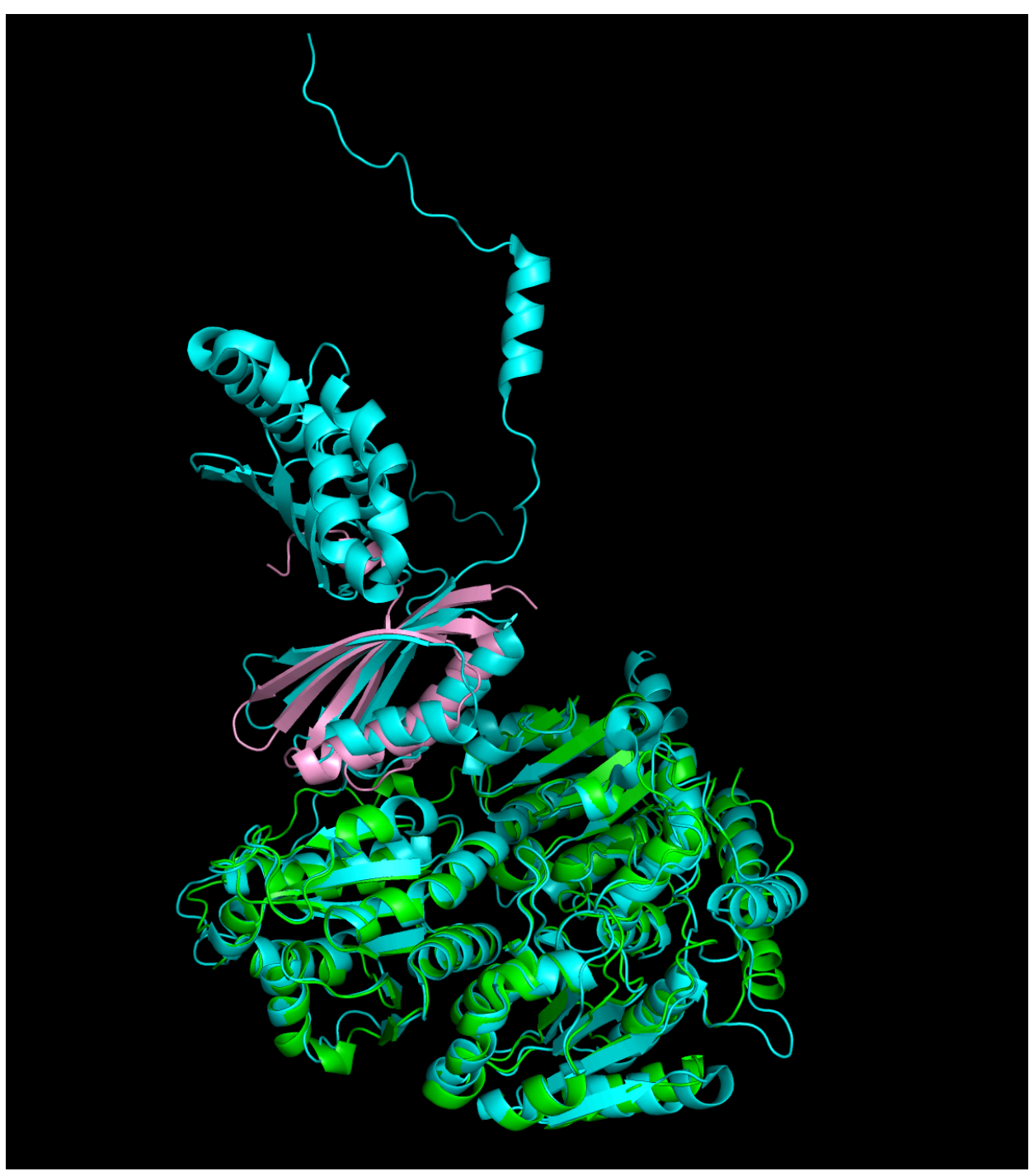

Figure S6: Acetolactate synthase (AHAS) from *Korarchaeum cryptofilum* with a putative short regulatory subunit. The predicted structure for the *K. cryptofilum* enzyme (with catalytic subunit in green and regulatory subunit in pink) was aligned to the experimental structure for the yeast enzyme (in cyan) by using the align command of the PyMOL Molecular Graphics System (Version 2.5.2, Schrödinger, LLC). The yeast enzyme includes five copies of each subunit; only one copy of each is shown here (chains A and C from PDB:6U9D).

We identified another putative truncated regulatory subunit in many strains of *Chryseobacterium* (i.e., UniProt A0A316WEF5). This gene is encoded adjacent to the catalytic subunit, and is over 40% identical to the N-terminal part of a regulatory subunit from *Pedobacter* (CA265_RS15810, which was confirmed by fitness data). We found that two of these strains, *C. viscerum* GW821-FHT04C04 *and C. viscerum GW821-FHT04A06*, grow in the absence of any one of the branched-chain amino acids, so the one-domain protein is presumably functional, and it is included in the revised GapMind as a prediction. Specifically, we inoculated each strain at OD_{600} = 0.02 into a 96-well plate with a defined base medium (NLDM_defined_noCarbon), 30 mM of PIPES sesquisodium salt as a buffer, 20 mM glucose as a carbon source, and 50 µM of each amino acid, with any one of isoleucine, leucine, or valine omitted from some wells. After aerobic growth with shaking at 30°C for 138 hours, the OD in the wells that lacked a branched-chain amino acid increased by 0.27 - 0.39.

Bibliography

Abramson, J., J. Adler, J. Dunger, R. Evans, T. Green, A. Pritzel, O. Ronneberger, L. Willmore, et al. 2024. Accurate structure prediction of biomolecular interactions with AlphaFold 3. *Nature* 630: 493–500. doi:10.1038/s41586-024-07487-w.

Ashniev, G. A., S. N. Petrov, S. N. Iablokov, and D. A. Rodionov. 2022. Genomics-Based Reconstruction and Predictive Profiling of Amino Acid Biosynthesis in the Human Gut Microbiome. *Microorganisms* 10. doi:10.3390/microorganisms10040740.

Biggs, B. W., M. N. Price, D. Lai, J. Escobedo, Y. Fortanel, Y. Y. Huang, K. Kim, V. V. Trotter, et al. 2024. High-throughput protein characterization by complementation using DNA barcoded fragment libraries. *Molecular Systems Biology* 20: 1207–1229. doi:10.1038/s44320-024-00068-z.

Blöchl, E., R. Rachel, S. Burggraf, D. Hafenbradl, H. W. Jannasch, and K. O. Stetter. 1997. Pyrolobus fumarii, gen. and sp. nov., represents a novel group of archaea, extending the upper temperature limit for life to 113 degrees C. *Extremophiles: Life Under Extreme Conditions* 1: 14–21. doi:10.1007/s007920050010.

Burnat, M., S. Picossi, A. Valladares, A. Herrero, and E. Flores. 2019. Catabolic pathway of arginine in Anabaena involves a novel bifunctional enzyme that produces proline from arginine. *Molecular Microbiology* 111: 883–897. doi:10.1111/mmi.14203.

Day, L. A., H. K. Carlson, D. R. Fonseca, A. P. Arkin, M. N. Price, A. M. Deutschbauer, and K. C. Costa. 2024. High-throughput genetics enables identification of nutrient utilization and accessory energy metabolism genes in a model methanogen. *mBio* 15: e0078124. doi:10.1128/mbio.00781-24.

Fahrbach, M., J. Kuever, M. Remesch, B. E. Huber, P. Kämpfer, W. Dott, and J. Hollender. 2008. *Steroidobacter denitrificans* gen. nov., sp. nov., a steroidal hormone-degrading gammaproteobacterium. *International Journal of Systematic and Evolutionary Microbiology* 58: 2215–2223. doi:10.1099/ijs.0.65342-0.

Graupner, M., and R. H. White. 2001. Methanococcus jannaschii generates L-proline by cyclization of L-ornithine. *Journal of Bacteriology* 183: 5203–5205. doi:10.1128/JB.183.17.5203-5205.2001.

Haft, D. H., J. D. Selengut, R. A. Richter, D. Harkins, M. K. Basu, and E. Beck. 2013. Tigrfams and genome properties in 2013. *Nucleic Acids Research* 41: D387-95. doi:10.1093/nar/gks1234.

Hasebe, F., K. Adachi, C. Maruyama, and Y. Hamano. 2024. Discovery of a novel methionine biosynthetic route via O-phospho-l-homoserine. *Applied and Environmental Microbiology* 90: e0124724. doi:10.1128/aem.01247-24.

Huang, Y. Y., M. N. Price, A. Hung, O. Gal-Oz, S. Tripathi, C. W. Smith, D. Ho, H. Carion, et al. 2024. Barcoded overexpression screens in gut Bacteroidales identify genes with roles in carbon utilization and stress resistance. *Nature Communications* 15: 6618. doi:10.1038/s41467-024-50124-3.

van Kempen, M., S. S. Kim, C. Tumescheit, M. Mirdita, J. Lee, C. L. M. Gilchrist, J. Söding, and M. Steinegger. 2024. Fast and accurate protein structure search with Foldseek. *Nature Biotechnology* 42: 243–246. doi:10.1038/s41587-023-01773-0.

Klemke, F., A. Baier, H. Knoop, R. Kern, J. Jablonsky, G. Beyer, T. Volkmer, R. Steuer, et al. 2015. Identification of the light-independent phosphoserine pathway as an additional source of serine in the cyanobacterium Synechocystis sp. PCC 6803. *Microbiology* 161: 1050–1060. doi:10.1099/mic.0.000055.

Liu, H., A. L. Shiver, M. N. Price, H. K. Carlson, V. V. Trotter, Y. Chen, V. Escalante, J. Ray, et al. 2021. Functional genetics of human gut commensal Bacteroides thetaiotaomicron reveals metabolic requirements for growth across environments. *Cell reports* 34: 108789. doi:10.1016/j.celrep.2021.108789.

Luneau, J. S., M. Baudin, T. Quiroz Monnens, S. Carrère, O. Bouchez, M.-F. Jardinaud, C. Gris, J. François, et al. 2022. Genome-wide identification of fitness determinants in the Xanthomonas campestris bacterial pathogen during early stages of plant infection. *The New Phytologist* 236: 235–248. doi:10.1111/nph.18313.

Luque, J. M. C. 2010. Discovery of novel pathways of microbial arginine biosynthesis. Doctoral dissertation, Children's National Medical Center.

Mendler, K., H. Chen, D. H. Parks, B. Lobb, L. A. Hug, and A. C. Doxey. 2019. AnnoTree: visualization and exploration of a functionally annotated microbial tree of life. *Nucleic Acids Research* 47: 4442–4448. doi:10.1093/nar/gkz246.

van der Oost, J., M. A. Huynen, and C. H. Verhees. 2002. Molecular characterization of phosphoglycerate mutase in archaea. *FEMS Microbiology Letters* 212: 111–120. doi:10.1111/j.1574-6968.2002.tb11253.x.

Passaro, S., G. Corso, J. Wohlwend, M. Reveiz, S. Thaler, V. R. Somnath, N. Getz, T. Portnoi, et al. 2025. Boltz-2: Towards Accurate and Efficient Binding Affinity Prediction. *BioRxiv*. doi:10.1101/2025.06.14.659707.

Potters, M. B., B. T. Solow, K. M. Bischoff, D. E. Graham, B. H. Lower, R. Helm, and P. J. Kennelly. 2003. Phosphoprotein with phosphoglycerate mutase activity from the archaeon Sulfolobus solfataricus. *Journal of Bacteriology* 185: 2112–2121. doi:10.1128/JB.185.7.2112-2121.2003.

Price, M. N., and A. P. Arkin. 2024. A fast comparative genome browser for diverse bacteria and archaea. *Plos One* 19: e0301871. doi:10.1371/journal.pone.0301871.

Price, M. N., A. M. Deutschbauer, and A. P. Arkin. 2020. Gapmind: automated annotation of amino acid biosynthesis. *mSystems* 5. doi:10.1128/mSystems.00291-20.

Price, M. N., A. M. Deutschbauer, and A. P. Arkin. 2021. Four families of folate-independent methionine synthases. *PLoS Genetics* 17: e1009342. doi:10.1371/journal.pgen.1009342.

Shiver, A. L., J. Sun, R. Culver, A. Violette, C. Wynter, M. Nieckarz, S. P. Mattiello, P. K. Sekhon, et al. 2025. Genome-scale resources in the infant gut symbiont Bifidobacterium breve reveal genetic determinants of colonization and host-microbe interactions. *Cell* 188: 2003-2021.e19. doi:10.1016/j.cell.2025.02.010.

Shi, D., H. Morizono, J. Cabrera-Luque, X. Yu, L. Roth, M. H. Malamy, N. M. Allewell, and M. Tuchman. 2006. Structure and catalytic mechanism of a novel N-succinyl-L-ornithine transcarbamylase in arginine biosynthesis of Bacteroides fragilis. *The Journal of Biological Chemistry* 281: 20623–20631. doi:10.1074/jbc.M601229200.

Shi, D., X. Yu, J. Cabrera-Luque, T. Y. Chen, L. Roth, H. Morizono, N. M. Allewell, and M. Tuchman. 2007. A single mutation in the active site swaps the substrate specificity of N-acetyl-L-ornithine transcarbamylase and N-succinyl-L-ornithine transcarbamylase. *Protein Science* 16: 1689–1699. doi:10.1110/ps.072919907.

Singh, S. K., K. Yang, S. Karthikeyan, T. Huynh, X. Zhang, M. A. Phillips, and H. Zhang. 2004. The thrH gene product of Pseudomonas aeruginosa is a dual activity enzyme with a novel phosphoserine:homoserine phosphotransferase activity. *The Journal of Biological Chemistry* 279: 13166–13173. doi:10.1074/jbc.M311393200.

Verstraete, M. M., C. Perez-Borrajero, K. L. Brown, D. E. Heinrichs, and M. E. P. Murphy. 2018. SbnI is a free serine kinase that generates O -phospho-l-serine for staphyloferrin B biosynthesis in Staphylococcus aureus. *The Journal of Biological Chemistry* 293: 6147–6160. doi:10.1074/jbc.RA118.001875.

Vyazmensky, M., Y. Zherdev, A. Slutzker, I. Belenky, O. Kryukov, Z. Barak, and D. M. Chipman. 2009. Interactions between large and small subunits of different acetohydroxyacid synthase isozymes of Escherichia coli. *Biochemistry* 48: 8731–8737. doi:10.1021/bi9009488.

Yoshida, A., T. Tomita, H. Atomi, T. Kuzuyama, and M. Nishiyama. 2016. Lysine Biosynthesis of Thermococcus kodakarensis with the Capacity to Function as an Ornithine Biosynthetic System. *The Journal of Biological Chemistry* 291: 21630–21643. doi:10.1074/jbc.M116.743021.

Yu, Y., P. Wang, H.-Y. Cao, Z.-J. Teng, Y. Zhu, M. Wang, A. McMinn, Y. Chen, et al. 2023. Novel D-glutamate catabolic pathway in marine Proteobacteria and halophilic archaea. *The ISME Journal* 17: 537–548. doi:10.1038/s41396-023-01364-6.
