# Supplementary material for: Novel enzymes for amino acid biosynthesis in diverse bacteria and archaea: GapMind 2026
Source: PLoS Genet. 2026 Nov 12;22(11):e1012372. doi: 10.1371/journal.pgen.1012372 (PMC13759103; doi:10.1371/journal.pgen.1012372)
Supplement: Supplementary file 2 [file pgen.1012372.s007.pdf]

"We noticed that you used the phrase 'unpublished data' in the manuscript."

We do not use that phrase, but our original submission did have four references to work in preparation.

1. "Close homologs of BT2402 ... are involved in threonine synthesis in the genus Phocaiecola" -- The data for P. vulgatus was already published. We updated the Fitness Browser and the figshare to include the data from P. dorei.

2. "For Rhodanobacter denitrificans FW104-10B01, we performed a variety of stress experiments in R2A medium, which will be described elsewhere" -- this data is publically available in the Fitness Browser. We also reworded the mention of this data.

3. "Close homologs of BT2402 (over 80% identical) from two species of a related genus, Phocaeicola, also seem to be involved in threonine synthesis..." Again, all of the data for Phocaeicola is now available. (Also, the public data set for P. dorei is larger than the one we originally analyzed and the cofitness value is now higher.)

4. "Because some of these strains of Chryseobacterium grow in the absence of any one of the branched-chain amino acids (i.e., GW821-FHT04C04 and GW821-FHT04A06; M. N. Price and H. P. Lesea, in preparation)" -- this text has been updated to include the increase in OD and the experimental details

Our original submission also referred to unpublished structures (PDB:6LD8, PDB:6LD9); this is not our data, and there is no associated publication. We changed the mention of these structures to "(PDB:6LD8, PDB:6LD9; there is no associated publication)"

Reviewer #1

The authors provide 7 sections describing novel or alternative enzymes for biosynthesis ... It is unclear though how and why these enzymes in the corresponding species were chosen for investigation. It would be great if the authors could connect these cases by some sort of summary in the beginning describing a "systematic approach" that they took to identify knowledge gaps with GapMind and elucidate these gaps in this study.

We added a new paragraph at the beginning of the results to describe our two main approaches for filling gaps. Since RB-TnSeq is our main approach, a lot of our work focuses on whatever organisms happen to have RB-TnSeq libraries. (Although a number of RB-TnSeq libraries are described in this manuscript for the first time, these were initially developed for other projects.)

We also added some text to clarify our definition of novel enzymes: we believe that these could not have been predicted from their similarity to known enzymes. So, the first part of the Results highlights the most novel findings.

"two genes which are important for fitness whenever glycine is not available...whether any other genes also fulfilled the criterion of being conditionally essential in glycine-free medium"

The data for Bifidobacterium breve show just three genes as important in the absence of glycine, but not in any of the other defined media experiments – *glyXL*, *glyXS*, and the potential glycine transaminase BBR_RS14535 that we discuss. Specifically, these are the only genes with fitness under -3 in both replicates for glycine-free medium and fitness of -2 or greater in all of the other 84 experiments with a defined medium. This is mentioned briefly in the revised results. In Methanococcus maripaludis, we examined the top 5 genes with the most similar fitness patterns to GlyXL (as defined by cofitness), besides GlyXS; all of them are still important for fitness when glycine is added, and based on their annotations, they are probably involved in other biosynthetic pathways. Also note that relatively few genes are shared between Methanococcus (an archaeon) and Bifidobacterium (a bacterium).

"Figure 1, it would be nice to add a schematic of GapMind workflow with inputs and outputs used to decide on the biosynthesis genes from bacterial genomes above the example output."

We added a schematic

Figure panel letters are part of titles above the graphs and are hard to notice. It would be better to put them next to the figure panels and align so it is clearer which letter corresponds to which panel.

To be honest, we find it easier to read if the figure panel letter is next to the title.

It would be helpful if the information about GlyXL and GlyXS co-occurrence mentioned on page 6 could be supported by a supplementary table.

For all of the comparative genomics results, the homologs (or lack theoreof) from individual genomes are now included in a figshare. For this case, see compare_glyXL_glyXS.tsv

It is not clear how the authors come to the conclusion that GlyXL-GlyXS produce glyoxylate (page 6) just from the fact that a putative transaminase is also essential in glycine-free conditions. Could the authors provide additional evidence that glyoxylate is indeed the intermediate of glycine biosynthesis, or at least discuss that this needs to be confirmed?

Besides the transaminase, there is also a putative negatively-charged binding pocket. But yes, we agree that biochemical studies are needed. We changed "propose" to "predict". Also, the caption for the new Table 1 mentions that all of our experimental evidence for the novel enzymes is genetic and that biochemical studies are needed.

It seems like the authors use different sets of representative genomes to perform analysis of different enzymes (Page 6 for GlyXL-GlyXS, page 7 for phosphoserine phosphatase from the DUF1015 family), which is a bit confusing. Ideally it would be nice to unify the set of genomes used for comparative genomics and provide supplementary tables supporting the claims about genes presence/absence, co-occurrence, and colocalization on the genome.

To ensure as much consistency as possible, for the revision, all of the comparative genomics analyses are based on the spring 2025 release of the *fast.genomics* database of representative genomes (one genome each for 7,312 genera of bacteria or archaea). The only exception is the comparison of the distribution of HomK and glycerate kinase, which relied on KEGG ortholog groups and so used AnnoTree instead of *fast.genomics*. The version of *fast.genomics* used is mentioned in the revised Methods and the tables of relevant homologs are provided in a figshare. (The genes for the DUF1015 figure are in browse_CDIF630erm_01132.tsv). For this specific instance -- because GlyXL/GlyXS are mostly present in different genomes than DUF1015, the genomes shown are different.

Related to the previous comment, why does a set of representative genomes mentioned on Page 7 contain a MAG? Wouldn't it make sense to filter representative genomes by quality, or even use only complete genomes of bacterial isolates for these types of analyses?

fast.genomics uses MAGs as a reference only if no isolate genome for that genus is available and only if the MAG is supposedly high-quality as defined by MIMAG, which means that besides the usual tests for completeness and contamination (from CheckM2), the MAG contains ribosomal RNAs and most tRNAs. Since most bacterial genera are known only from MAGs, excluding MAGs entirely might not be ideal. Also note that we used a complementation assay to validate a putative biosynthetic gene from a MAG (*serC* in Methanomethylophilaceae, listed in Table 2).

On page 13, authors use yet another representative set of genomes to investigate DapE. If possible, this analysis should be unified with others of that kind, and the corresponding supplementary table supporting the provided numbers of orthologs etc, should be provided.

Again, we used *fast.genomics* (the April 2025 release). The table in figshare is Echvi_1427_vs_dapE.tsv

Figure panels used to depict complementation assays should be unified across different figures – sometimes there are multiple lines, likely depicting replicates, and sometimes mean and min and max curves (Figure 5E). In all such panels, number of replicates should be reported in the legend.

Most of the complementation assays have 3 replicates of each condition, which is clarified in the revised figure legends. For Fig 5E, showing every replicate made for a busy plot. In particular, if every replicate is shown, it is very difficult to see the data for +CA265_RS23900 and +dapE, which are on top of each other.

On page 10, it is unclear why the authors "inferred that these IMPase-like proteins have another function. In particular, we predicted that these enzymes would be the missing phosphoserine phosphatases". Could the authors clarify their logic in more detail?

The revision explains that if the IMPases are histidinol phsophatases, then they should be genetically redundant with the HisN part of *hisBN*.

In Figure 4, it may be misleading to mark the genes "serA" and "serB" since serB is the predicted function. Would be more accurate to write locus tag or "IMPase-like serB".

We agree, the revised figure labels it "IMPase like".

On page 10, the authors write that the fact that serA and IMPase-like serB mutants increase their fitness in the presence of L-serine "confirms that these IMPase-like proteins are alternative phosphoserine phosphatases". While the fact that the mutants behave similarly suggests that they have similar function, I don't think the authors have enough evidence to claim that these data confirms the function of the enzyme.

We changed "confirms" to "supports our prediction"

In Figure 5, bacterial names should be in italics.

We made this change.

On Page 13, the authors write that they identified metB-like gene that has a similar fitness pattern as metA in four genera of Xanthomonadales. It would be helpful to provide more details on how this was achieved.

The revised text mentions that MetBX was annotated as MetB by the original GapMind

While it is nice to see Tables 1-3 with new enzymes added to GapMind, it is unclear how this section is connected to the rest of the manuscript. The authors write about "a number" of enzymes (Table 1 contains 13 lines, so it would be more accurate to write the number explicitly), of which 6 were functionally characterized in the literature and 3 were functionally characterized by the authors. But these are not the enzymes described in the first part of the manuscript. Why are the newly discovered enzymes not part of Table 1? And why do not the authors provide figures with experimental validation of three enzymes which they just describe in the text?

Since we describe the novel enzymes in detail in the main text, they were not included in a table, but we see

how this could be confusing. We added a new Table 1 near the beginning of the results to list the novel enzymes.

"...why do not the authors provide figures with experimental validation of three enzymes which they just describe in the text?"

In our original submission, there were four complementation assays that were only provided as final OD values. Growth curves for these are now in Figure S3D, S4A, S4B, and S4C. We also added the fitness values for two genes from *Brevundimonas vesicularis*$_C$ GW460-12-10-14-LB2 (in Appendix 5).

Figure 8, please add the number of genomes in each category to the figure.

The figure (now Fig 9) now includes this information.

Page 24, Updating GapMind section should also provide the versions of the other databases included in the current edition.

The revision includes version numbers for Swiss-Prot, MetaCyc, and BRENDA. (The Fitness Browser reannotations database does not have version numbers.)

The authors provide primer sequences and descriptions of cloning strategies...but the complete sequences themselves are not provided. I strongly recommend...providing these with the paper

A zip file with the maps and sequences of the plasmids constructed for this study, in genbank and snapgene formats, is now in S14 Dataset.

Fig 2: The inset labels are adequate but not very friendly to a reader who is eager to look at the data before reading the full caption.

We revised the labels

For (D), the caption should describe the nature of the replicates

The revised caption specifies that all three technical replicates are shown

Because this is the first data set / figure describing a complementation experiment, it might be worth adding a cartoon depicting the general scheme that will be used repeatedly (chromosomal knockout; SBOL Visual map of the complementation plasmid.

We added some more explanation to the text and to the legend of Figure 2. Since we're using a pretty standard approach for complementation, we don't think a plasmid map is necessary.

At a minimum, the fact that the complementation plasmid has the potential complement gene(s) on a pTet promoter is essential to include in the caption, not just Methods. This also applies to the Fig 3 caption and others.

We modified the caption of Fig 2 to mention the pTet promoter, and the first mention of the expression vector in the text of the Results now mentions that it is anhydrotetracycline-inducible expression vector. Since all of our complementation assays use the same vector, we don't think it's necessary to mention the pTet promoter in the other figures, but do we mention that our vector (pBb2A2c) is aTc-inducible.

Fig 4: Is there a serC on this plot? It also seems like it begs the question a little to label the red point as serB since the evidence is being drawn on to assign the label.

We relabeled serB to IMPase like and we labeled serC.

Fig 4, Fig 5: red and green contrasting labels should be avoided for color blind readers.

For Fig 4, there are now three labels, so it's difficult to avoid this issue, but we think the label placement makes things clear.

For Fig 5 A-D, the color information is not essential as the plotting symbol also shows which strand the insertions are on (and, the strand information is not central to our analysis anyway).

"This confirms that DapF2 is a diaminopimelate epimerase." I've noted generally that I myself would use weaker language than "confirm" in some cases than the authors do, but here I really think the claim needs to be weakened. There could definitely be other plausible explanations for why overexpression of a gene that is NOT a diaminopimelate epimerase could suppress the secretion of diaminopimelate through effects on other pathways or general cell stress. I would say the evidence here is strongly suggestive but not conclusive.

We changed "confirm" to "supports our hypothesis that"

"We used mass spectrometry to compare the supernatant from various strains of E. coli" I think moving the supplementary figure into the main text would improve readability. I also think dataset S1 should be referred to explicitly in the text as well as in the figure S4 caption.

The revision's Fig 8 (in the main text of the Results) includes some extracted ion chromatograms and an MS2 mirror plot, as well as the plots of peak height across samples. The relevant text of the Results refers to the Methods, which includes additional details.

"we harvested 20 OD600 units of the culture" 20 OD600 * mL?

yes, we corrected this

Were media pH adjusted? To what pH and with what acid/base? Final concentrations should have appropriate significant figures for the precision used.

It was adjusted to pH 7.0 with NaOH. We double checked the description of RCH2_defined_noCarbon, but we didn't understand the reviewer's concern about final concentrations and significant figures. It seems clear to us.

I may have missed it, but media "(RCH2_defined_noCarbon)" should be defined here even if part of another manuscript in preparation.

RCH2_defined_noCarbon is only mentioned in the RB-TnSeq assays section, in close proximity to where it is defined.

"https://gnps2.org/status?task=64d1b4b524674e42870ee88a4d6d8d6e;" the hyperlink has an extra ;

we edited this

"Most of the data is from (Liu et al. 2021)" How about the remaining data?

We revised this to "Except for the new nitrogen source data, the data is from (Liu et al. 2021)"

"Potential functional orthologs (above 25% of the maximum bit score) were never found together in the same genome." What is n here?

Steroidobacter ArgA was found in 109 genera and Pseudomonas ArgA was found in 970 genera.

Also the underlying data table, compareHomologs_argA_Steroidobacter_Pseudomonas.tsv, is in the comparative genomics figshare

Fig S3B: I haven't checked the red and green colors here for color blindness visibility but suspect they should be changed. The inset doesn't match the green color precisely.

The colors have been updated

Fig S4: Overlaid ion traces (with retention time) and integrated areas below would be a more familiar way to represent this data, at least to me. What's not clear from the caption is that retention time AND an external standard are used to validate the assay; the supplementary dataset S1 does make that clear but that's a bit too buried away I think.

The revised Fig 8 (now in the Results) includes ion intensity vs. retention time for two supernatants and the reference standard, as well as mirror plot for the MS2 fragmentation. The new Fig 10 (in the Methods) includes all of the extracted ion chromatograms (including for the standard).

A sentence or two more on the importance of / motivation for / possible applications of a full understanding of amino acid biosynthesis pathways could be useful.

We added an introductory sentence

"describe how we improved GapMind's coverage by annotating diverged enzymes," Glossing "diverged" could be helpful here.

GapMind considers candidates to be lower confidence if they are under 40% identical to a characterized enzyme (unless there is a curated HMM for this step). So, we use 40% identity as the threshold for diverged. This is briefly mentioned in the revised Introduction.

"Across diverse bacteria and archaea," ... It might be worth noting how many genera contain one of GlyXL or GlyXS but not both (to support the statement that they "usually" co-occur).

For consistency, we redid this analysis with the 2025 release of *fast.genomics*. Of 1,131 genomes with potential orthologs of GlyXL or GlyXS, 978 (86%) contain both.

An illustration of the glycine synthesis pathway could be helpful. Wherever these pathways are shown (e.g. Fig 3A), this biochemist and organic chemist would appreciate structures rather than just names of the metabolites. Adding gene names with the steps consistently (present in 6A, absent in 3A) would be helpful too.

Both 3A and 6A do have gene names (SerCAB and Hom, MesDX, MetABCFEHZ)

For glycine synthesis, we don't have a strong proposal for the molecular function of GlyXL, but we don't expect it to involve the folate pool, so we don't think that showing the detailed reaction of serine hydroxymethyltransferase (GlyA) would be so helpful. More generally, we agree that compound structures can be helpful, but fitting them all into many of our figures would be challenging. The revision recommends using EcoCyc to see pathways together with compound structures.

"prediction tool Boltz-2x (Passaro et al. 2025) identified a positively-charged binding pocket for glyoxylate in GlyXL," Adding "(see Methods)" would direct readers to the details about which structures/homologs

are being used.

we made this change

"BBR_RS14535 is annotated as a glyoxylate transaminase." > "putative glyoxylate transaminase"?

We agree that our experimental evidence for this protein is more suggestive than proof. But, in GapMind, BBR_RS14535 *is* annotated as a glyoxylate transaminase, in the sense that homologs (above 40% identity) are considered high-confidence candidates for this step.

"as well as another potential histidinol-phosphate phosphatase from the inositol monophosphatase (IMPase) family." I wondered about the operon/genome location of this relative to other biosynthetic genes

There's no gene neighoborhood link. It's in an apparent operon with a type II secretion protein N, ADP-sugar diphosphatase nudE, 3'(2'),5'-bisphosphate nucleotidase cysQ, and NTP pyrophosphohydrolase mazG. These genes mostly don't seem to be functionally related to each other or to serine synthesis (although cysQ is involved in sulfate assimilation, which is linked to the biosynthesis of other amino acids).

Fig 5: I find the full-height 5E distracting. Maybe a 3-row x 2-column plot with the transposon panels A-D and then E centered below it?

We changed the plot's layout.

Fig 6: Why not put an inset on the RB-TnSeq data as in Fig 2 that describes the color highlighting in addition to the caption? Keeping consistency between figures throughout would help the reader settle into some of the common patterns. Adding a cofitness statistic to the panels would be helpful.

For 6B, we added an inset that describes the color coding.

For cofitness -- as mentioned in the text, in the four mutant libraries from Xanthomonadales shown in Fig 6B, cofitness(metA, metBX) ranges from 0.79 to 0.94. But we think the reader can see that the fitness patterns are similar; we don't think the exact values are so important.

For Fig 6C, is there a hom analog on the plot?

Yes, but it's complicated, and it doesn't lead to any definite conclusion. First, homoserine is a precursor for both methionine and threonine. Second, there are two likely hom proteins in the genome, which we suspect are partially genetically redundant. These are OKGIIK_01520 (encoded downstream of *metA* and *metBX*) and OKGIIK_13560 (encoded upstream of *thrBC*). OKGIIK_01520 has no mutant phenotypes and OKGIIK_13560 is moderately important for fitness regardless of whether methionine is added (fitness values around -2). We suspect that under our growth conditions, OKGIIK_13560 is the main source of homoserine for the synthesis of both methionine and threonine.

"a mutant library of Rhodanobacter sp. FW510-T8" I'm not clear on the rationale for choosing this strain particularly.

There's no particular reason; we were working with this mutant library at the time we designed this experiment.

"Just four steps account for 65% of the remaining gaps:" might be clearer as "the 143 remaining gaps" or "the gaps remaining from the latest revision of GapMind"

Yes, we changed it to "143 remaining gaps"

"Even for the Pseudomonadota, ... (the average goes from 1.27 to 1.26)." I found this section a bit confusing while reading, although I think I grasp the idea.

We added "In other words, we filled a higher proportion of the gaps in the studied Pseudomonadota than in the typical Pseudomonadota"

Fig 8: Why not add the exact numbers 3, 4 and 5? I don't think the figure would be too crowded.

We made this change.

"For metB, GapMind identified two medium-confidence candidates, both of which have greater similarity to methionine gamma-lyases (73-77% identity) than to known MetB proteins (44-48%)." Is there an automated step involved in GapMind which could perform this kind of check?

These are medium confidence *because* of this suspicious similarity to proteins with other functions. We changed the text to reflect this.

"We moved the pKTO49_NN1 plasmid library into the E. coli conjugation donor WM3064 via electroporation to make the conjugation donor strain AMD1385." If available, estimating the library size at this point (based on number of transformants) could be useful for others.

The revision mentions that we used barcode sequencing to estimate the diversity of AMD1385 (>7 million barcodes).

Adding the closure type for tubes, the bottom geometry of well plates (2 mL deep well?), and the stroke length of the shaker would be helpful.

We added experimental details.

"Although this homology is remote" I would prefer "sequence similarity" here myself, I try to treat "homology" as binary.

In this case, there's no detectable sequence similarity; it's structural similarity. We changed it to "the structural similarity is remote"

"We speculate that other kinases, phosphatases, or pyrophosphatases have evolved to act on these substrates and sometimes replace the known families. In any case, the absence of these genes should not be used to predict auxotrophies." I wonder whether GapMind already does, or could, include an automated recommendation when the only gaps remaining in a pathway are phosphotransfer-related, to this effect.

We'd prefer a solution that applies to all gaps, not just these four steps. GapMind already has a concept of "known gaps" that is intended to address this issue. If an organism is known to be prototrophic despite lacking (say) *serB*, then if a related organism (roughly speaking, from the same taxonomic family) is missing serB, then GapMind warns that the gap might not be genuine. This can be seen as the question marks for *serC* and *serB* in Fig 1B. In our current collection of ~200 confirmed prototrophs, we can label all the gaps as known gaps, but this probably doesn't generalize well, given the incredible diversity of bacteria and archaea. The hope is that over time, prototrophy will be confirmed experimentally for more diverse prokaryotes and GapMind will be able to label most of the spurious gaps (in prototrophic genomes) as known gaps. But now, there are all these metagenome-assembled genomes, many of which belong to taxonomic families that have never been cultured, and there's papers about what happens when you run GapMind on MAGs. If we can't culture the organisms, then our plan for "known gaps" isn't going to work. So, we're thinking about what to do, but we don't have a great plan yet.
