# Supplementary material for: Novel enzymes for amino acid biosynthesis in diverse bacteria and archaea: GapMind 2026
Source: PLoS Genet. 2026 Nov 12;22(11):e1012372. doi: 10.1371/journal.pgen.1012372 (PMC13759103; doi:10.1371/journal.pgen.1012372)
Supplement: Peer review history

# Peer review: Decision Letter 0

**DOI:** 10.1371/journal.pgen.1012372.r001

**Authors:** Sean Crosson, Ankur B. Dalia

23 Sep 2026

PGENETICS-D-26-00751

Novel enzymes for amino acid biosynthesis in diverse bacteria and archaea: GapMind 2026

PLOS Genetics

Dear Dr. Price,

Thank you for submitting your manuscript to PLOS Genetics. After careful consideration, we feel that it has merit but does not fully meet PLOS Genetics's publication criteria as it currently stands. Therefore, we invite you to submit a revised version of the manuscript that addresses the points raised during the review process.

Please submit your revised manuscript within by Oct 23 2026 11:59PM. If you will need more time than this to complete your revisions, please reply to this message or contact the journal office at plosgenetics@plos.org. When you're ready to submit your revision, log on to https://www.editorialmanager.com/pgenetics/ and select the 'Submissions Needing Revision' folder to locate your manuscript file.

Please include the following items when submitting your revised manuscript:

* A letter that responds to each point raised by the editor and reviewer(s). You should upload this letter as a separate file labeled 'Response to Reviewers'. This file does not need to include responses to formatting updates and technical items listed in the 'Journal Requirements' section below.

* A marked-up copy of your manuscript that highlights changes made to the original version. You should upload this as a separate file labeled 'Revised Manuscript with Track Changes'.

* An unmarked version of your revised paper without tracked changes. You should upload this as a separate file labeled 'Manuscript'.

If you would like to make changes to your financial disclosure, competing interests statement, or data availability statement, please make these updates within the submission form at the time of resubmission. Guidelines for resubmitting your figure files are available below the reviewer comments at the end of this letter.

As the corresponding author, your ORCID iD is verified in the submission system and will appear in the published article. PLOS supports the use of ORCID, and we encourage all coauthors to register for an ORCID iD and use it as well. Please encourage your coauthors to verify their ORCID iD within the submission system before final acceptance, as unverified ORCID iDs will not appear in the published article. Only the individual author can complete the verification step; PLOS staff cannot verify ORCID iDs on behalf of authors.

We look forward to receiving your revised manuscript.

Kind regards,

Ankur B. Dalia

Academic Editor

PLOS Genetics

Sean Crosson

Section Editor

PLOS Genetics

Aimée Dudley

Editor-in-Chief

PLOS Genetics

Anne Goriely

Editor-in-Chief

PLOS Genetics

**Additional Editor Comments (if provided):**

As you will see below, both reviewers were highly enthusiastic and supportive of your initial submission. But they each raise minor, but important, points that should be addressed in a revised draft.

**Journal Requirements:**

If the reviewer comments include a recommendation to cite specific previously published works, please review and evaluate these publications to determine whether they are relevant and should be cited. There is no requirement to cite these works unless the editor has indicated otherwise.

1) We ask that a manuscript source file is provided at Revision. Please upload your manuscript file as a .doc, .docx, .rtf or .tex. If you are providing a .tex file, please upload it under the item type u2018LaTeX Source Fileu2019 and leave your .pdf version as the item type u2018Manuscriptu2019.

2) We noticed that you used the phrase 'unpublished data' in the manuscript. We do not allow these references, as the PLOS data access policy requires that all data be either published with the manuscript or made available in a publicly accessible database. Please amend the supplementary material to include the referenced data or remove the references.

3) Please upload all main figures as separate Figure files in .tif or .eps format. For more information about how to convert and format your figure files please see our guidelines:

https://journals.plos.org/plosgenetics/s/figures

4) Please upload a copy of Figure Fig 1-8 which you refer to in your text on pages 2, 4, 6, 10, 11, 13, 16, and 22. Or, if the figure is no longer to be included as part of the submission please remove all reference to it within the text.

5) Please amend your detailed Financial Disclosure statement. This is published with the article. It must therefore be completed in full sentences and contain the exact wording you wish to be published.

i) State what role the funders took in the study. If the funders had no role in your study, please state: "The funders had no role in study design, data collection and analysis, decision to publish, or preparation of the manuscript."

ii) If any authors received a salary from any of your funders, please state which authors and which funders..

If you did not receive any funding for this study, please simply state: u201cThe authors received no specific funding for this work.u201d

**Reviewers' comments:**

Reviewer's Responses to Questions

**Comments to the Authors:**

**Please note here if the review is uploaded as an attachment.**

Reviewer #1: Price and colleagues present an updated version of GapMind, the tool for predicting enzymes involved in amino acid biosynthesis in prokaryotes based on the genome sequence, which now include novel enzymes which the authors identified with high-throughput genetics and comparative genomics approaches. The authors identified and experimentally confirmed ten novel genes involved in glycine, serine, methionine and branched-chain amino acids; this information was incorporated in GapMind. This update lead to improved predictions of amino acid biosynthesis pathways and auxotrophies for a subset of species for which experimental information is available.

Overall, this is an important contribution to unraveling novel enzymes and enzymatic functions in bacteria, and experimental tour-de-force to elucidate the remaining gaps in our knowledge on bacterial amino acid biosynthesis. While the authors refer to a systematic approach to fill these gaps, it is not always understandable from the sections of the paper how they belong together, and why certain genes were selected for investigation. Below are a few suggestions that may help to improve the flow of the manuscript and clarify the importance of the findings.

1. The authors provide 7 sections describing novel or alternative enzymes for biosynthesis of different amino acids, which they discover with different approaches (comparative genomics, transposon mutant libraries) and experimentally confirm by expression the enzymes in E.coli mutants. It is unclear though how and why these enzymes in the corresponding species were chosen for investigation. It would be great if the authors could connect these cases by some sort of summary in the beginning describing a “systematic approach” that they took to identify knowledge gaps with GapMind and elucidate these gaps in this study. It is also understandable if all these different cases came from different studies using GapMind for bacteria of interest, but this can also be mentioned in the beginning, in the first paragraph of the results.

2. Page 4, novel genes for glycine synthesis via glyoxylate, the authors write that they identified “two genes which are important for fitness whenever glycine is not available” and refer to Figure 2A and B. While Figures 2A and 2B show fitness of the identified genes, they do not illustrate how these particular genes were selected, and whether any other genes also fulfilled the criterion of being conditionally essential in glycine-free medium. It would be helpful and illustrative to add a supplementary figure with genes on the x-axis, and fitness across different conditions on the y-axis, where genes could be sorted by average fitness in glycine-free condition. This will demonstrate how many genes are glycine-specific and hopefully explain why the two chosen by the authors are the best (or even the only) candidates.

3. Figure 1, it would be nice to add a schematic of GapMind workflow with inputs and outputs used to decide on the biosynthesis genes from bacterial genomes above the example output.

4. Figure panel letters are part of titles above the graphs and are hard to notice. It would be better to put them next to the figure panels and align so it is clearer which letter corresponds to which panel.

5. It would be helpful if the information about GlyXL and GlyXS co-occurrence mentioned on page 6 could be supported by a supplementary table.

6. It is not clear how the authors come to the conclusion that GlyXL-GlyXS produce glyoxylate (page 6) just from the fact that a putative transaminase is also essential in glycine-free conditions. Could the authors provide additional evidence that glyoxylate is indeed the intermediate of glycine biosynthesis, or at least discuss that this needs to be confirmed?

7. It seems like the authors use different sets of representative genomes to perform analysis of different enzymes (Page 6 for GlyXL-GlyXS, page 7 for phosphoserine phosphatase from the DUF1015 family), which is a bit confusing. Ideally it would be nice to unify the set of genomes used for comparative genomics and provide supplementary tables supporting the claims about genes presence/absence, co-occurrence, and colocalization on the genome.

8. Related to the previous comment, why does a set of representative genomes mentioned on Page 7 contain a MAG? Wouldn’t it make sense to filter representative genomes by quality, or even use only complete genomes of bacterial isolates for these types of analyses?

9. On page 13, authors use yet another representative set of genomes to investigate DapE. If possible, this analysis should be unified with others of that kind, and the corresponding supplementary table supporting the provided numbers of orthologs etc, should be provided.

10. Figure panels used to depict complementation assays should be unified across different figures – sometimes there are multiple lines, likely depicting replicates, and sometimes mean and min and max curves (Figure 5E). In all such panels, number of replicates should be reported in the legend.

11. On page 10, it is unclear why the authors “inferred that these IMPase-like proteins have another function. In particular, we predicted that these enzymes would be the missing phosphoserine phosphatases”. Could the authors clarify their logic in more detail?

12. In Figure 4, it may be misleading to mark the genes “serA” and “serB” since serB is the predicted function. Would be more accurate to write locus tag or “IMPase-like serB”.

13. On page 10, the authors write that the fact that serA and IMPase-like serB mutants increase their fitness in the presence of L-serine “confirms that these IMPase-like proteins are alternative phosphoserine phosphatases”. While the fact that the mutants behave similarly suggests that they have similar function, I don’t think the authors have enough evidence to claim that these data confirms the function of the enzyme.

14. In Figure 5, bacterial names should be in italics.

15. On Page 13, the authors write that they identified metB-like gene that has a similar fitness pattern as metA in four genera of Xanthomonadales. It would be helpful to provide more details on how this was achieved.

16. While it is nice to see Tables 1-3 with new enzymes added to GapMind, it is unclear how this section is connected to the rest of the manuscript. The authors write about “a number” of enzymes (Table 1 contains 13 lines, so it would be more accurate to write the number explicitly), of which 6 were functionally characterized in the literature and 3 were functionally characterized by the authors. But these are not the enzymes described in the first part of the manuscript. Why are the newly discovered enzymes not part of Table 1? And why do not the authors provide figures with experimental validation of three enzymes which they just describe in the text?

17. Figure 8, please add the number of genomes in each category to the figure.

18. Page 24, Updating GapMind section should also provide the versions of the other databases included in the current edition.

Reviewer #2: I am reviewing this manuscript as a biochemist and researcher with experience in bacterial genetics and metabolic engineering, although I am not a metabolism specialist. I have no connection to the authors other than their affiliation with my PhD institution. I have no professional stake or interest in the subject matter of this paper. (I provide this context and sign my reviews because I believe my background and experience as a scientist can’t help but have a major impact on my perspective and that some idea of what I’m more and less qualified to comment on makes my comments more useful to the authors. I also believe it’s my responsibility as a reviewer to stand behind any critique I offer and to try to provide it in a supportive way to help improve the work.)

The manuscript presents a very wide-ranging collection of results and discussions aimed at closing gaps in annotation of amino acid biosynthesis pathways across bacteria and archaea, integrated through the authors’ tool GapMind. The sources of evidence for new annotations include collated literature results, bioinformatic predictions, new or previously published transposon library sequencing, and in many cases, experimental validations at the phenotypic level through growth complementation. This manuscript illustrates both the importance and difficulty of integrating information about microbial genomes and making it broadly useful to the scientific community. Publishing findings is not enough on its own because finding and connecting the results to new problems and questions is a project in itself that many people won’t attempt. Instead, as the authors show, considerable original followup work – some of it experimental, but the non-experimental work is not unimportant – needs to be done to draw connections and maximize the predictive value of data sets. That work is entirely appropriate subject matter for a paper like this one. Then it must be made available in database form, with an accessible and low-barrier interface, to be as useful as possible. The authors have accomplished all of this. Having tried out a number of queries on GapMind I find it easy to use, transparent, and thorough, with strong links to accessory and related tools directly in the user-facing output. The complete web implementation makes access easy.

There are definitely some challenges to collecting and publishing this kind of investigation, particularly in terms of crafting narratives and shifting between different levels of evidence and certainty during the discussion, and the manuscript seems mindful of these challenges. Similar approaches and experiments across organisms and enzymes are described pretty consistently which helps with clarity. I find the practical validation of the new GapMind database in terms of average gaps per genome in a test set of microbes to be persuasive and compelling, and the manuscript draws out themes appropriately where they exist. It is pretty clear to me that there is a lot that needs to be laid out and discussed, and not all the vignettes can be equally solidly supported or complex. The manuscript uses a tiered approach where many smaller stories (but also some substantial ones) are placed in the Appendix, grouped by theme, I guess with an eye to the paper’s overall readability. I’m not sure it wouldn’t read better to me if everything was simply laid out at the same level of prominence (almost no Appendix sections) despite the differences in length/detail, though the journal may have some opinions about this. It seems like a tough or even arbitrary call in some cases what to put where, and I’m not sure it’s possible to avoid substantial shagginess, but I wouldn’t cut any of this material out or further condense it. Probably deciding what to put in this manuscript instead of elsewhere, and when to stop at medium-certainty predictions, has posed some challenges. But all this just seems like it is in the nature of the problem, which has to be chipped away at with this kind of variability.

The manuscript is circumspect and thorough in its reasoning, suiting the claims to the level of evidence available. I myself would use less conclusive language around many of the functional claims, since even genetic complementation assays are not as definitive as direct biochemical experiments that show a specific activity, even if they are very convincing (and appropriately controlled). From the background I can supply as a reviewer, I do not see any issues with the authors’ interpretation of RB-TnSeq data or the various bioinformatic steps, which are clearly explained. In some cases it seemed while reading like there was not enough specific documentation about the sequences and residues being invoked in homolog comparisons, but I see that the SitesBLAST tool basically reproduces the analysis with very low barrier to getting the full details, so that seems like it should be sufficient. The figures are consistent, sufficient, and clear, with some room in a few places to tweak for greater clarity.

Overall I have suggestions for minor changes but consider this manuscript nearly ready to publish. I appreciate the authors’ pursuit of this project and their attention to detail and consistency.

B Thuronyi, Williams College

No LLMs or generative AI were used as part of my review process or preparation. I have not reproduced the authors’ analysis or substantially reviewed their raw data other than spot checking to make sure it is available. I have not verified the accuracy of references.

I include numbered points below separated into categories. The references are to page numbers using the authors’ numbering.

Major points to address: I believe these must be addressed before publication because they present serious problems for soundness, reproducibility, or clarity.

Minor points to address: In my opinion, these should definitely be addressed, and hopefully the authors agree, but I don’t consider any of them foundational or critical (although some of them are clearly errors).

Suggestions & Comments: I do not want to put any pressure on the authors to make any revisions to respond to or revise based on these points. I offer them based on my own experience and opinions and as a colleague who has read the manuscript in detail and may have a useful perspective that the authors are free to take or leave. Suggestions are meant to be actionable, Comments are usually not.

Copy editing: I include these because I happened to notice them in my close reading and felt I might as well note them down in case they are useful to the authors in proofreading. I don’t mean to imply by listing them that the manuscript has insufficient attention to detail or that typos or other small errors have any bearing on the quality of the work.

Major points to address:

1. 31 - The authors provide primer sequences and descriptions of cloning strategies that are probably sufficient to piece together the exact sequences of their plamids, but the complete sequences themselves are not provided. I strongly recommend, where they are readily available, providing these with the paper, and including the authors' annotations. I coauthored a piece calling for publishing complete annotated sequences (certainly of plasmids, supported by sequencing, but also of modified loci) that lays out the rationale for this: https://journals.plos.org/plosbiology/article?id=10.1371/journal.pbio.3002376.

Minor points to address:

2. 5 - Fig 2: The inset labels are adequate but not very friendly to a reader who is eager to look at the data before reading the full caption. For (D), the caption should describe the nature of the replicates -- especially since in other, similar figures the lines do NOT represent individual biological replicate traces (e.g. Fig 5E). Because this is the first data set / figure describing a complementation experiment, it might be worth adding a cartoon depicting the general scheme that will be used repeatedly (chromosomal knockout; SBOL Visual map of the complementation plasmid. At a minimum, the fact that the complementation plasmid has the potential complement gene(s) on a pTet promoter is essential to include in the caption, not just Methods. This also applies to the Fig 3 caption and others.

3. 11 - Fig 4: Is there a serC on this plot? It also seems like it begs the question a little to label the red point as serB since the evidence is being drawn on to assign the label.

4. 11 - Fig 4, Fig 5: red and green contrasting labels should be avoided for color blind readers.

5. 19 - "This confirms that DapF2 is a diaminopimelate epimerase." I've noted generally that I myself would use weaker language than "confirm" in some cases than the authors do, but here I really think the claim needs to be weakened. There could definitely be other plausible explanations for why overexpression of a gene that is NOT a diaminopimelate epimerase could suppress the secretion of diaminopimelate through effects on other pathways or general cell stress. I would say the evidence here is strongly suggestive but not conclusive.

6. 19 - "We used mass spectrometry to compare the supernatant from various strains of E. coli" I think moving the supplementary figure into the main text would improve readability. I also think dataset S1 should be referred to explicitly in the text as well as in the figure S4 caption.

7. 27 - "we harvested 20 OD600 units of the culture" 20 OD600 * mL?

8. 30 - Were media pH adjusted? To what pH and with what acid/base? Final concentrations should have appropriate significant figures for the precision used.

9. 30 - I may have missed it, but media "(RCH2_defined_noCarbon)" should be defined here even if part of another manuscript in preparation.

10. 33 - "https://gnps2.org/status?task=64d1b4b524674e42870ee88a4d6d8d6e;" the hyperlink has an extra ;

11. 34 - "Most of the data is from (Liu et al. 2021)" How about the remaining data? (There is a joke about a farmer who always describes his white sheep first, and when asked about the black ones says the same thing, but I'll avoid retelling it here.)

12. 35 - "Potential functional orthologs (above 25% of the maximum bit score) were never found together in the same genome." What is n here?

13. 37 - Fig S3B: I haven't checked the red and green colors here for color blindness visibility but suspect they should be changed. The inset doesn't match the green color precisely.

14. 40 - Fig S4: Overlaid ion traces (with retention time) and integrated areas below would be a more familiar way to represent this data, at least to me. What's not clear from the caption is that retention time AND an external standard are used to validate the assay; the supplementary dataset S1 does make that clear but that's a bit too buried away I think.

Suggestions:

15. 1 - A sentence or two more on the importance of / motivation for / possible applications of a full understanding of amino acid biosynthesis pathways could be useful.

16. 4 - "describe how we improved GapMind’s coverage by annotating diverged enzymes," Glossing "diverged" could be helpful here.

17. 6 - "Across diverse bacteria and archaea," ... It might be worth noting how many genera contain one of GlyXL or GlyXS but not both (to support the statement that they "usually" co-occur).

18. 6 - An illustration of the glycine synthesis pathway could be helpful. Wherever these pathways are shown (e.g. Fig 3A), this biochemist and organic chemist would appreciate structures rather than just names of the metabolites. Adding gene names with the steps consistently (present in 6A, absent in 3A) would be helpful too.

19. 6 - "prediction tool Boltz-2x (Passaro et al. 2025) identified a positively-charged binding pocket for glyoxylate in GlyXL," Adding "(see Methods)" would direct readers to the details about which structures/homologs are being used.

20. 6 - "BBR_RS14535 is annotated as a glyoxylate transaminase." > "putative glyoxylate transaminase"?

21. 10 - "as well as another potential histidinol-phosphate phosphatase from the inositol monophosphatase (IMPase) family." I wondered about the operon/genome location of this relative to other biosynthetic genes

22. 12 - Fig 5: I find the full-height 5E distracting. Maybe a 3-row x 2-column plot with the transposon panels A-D and then E centered below it?

23. 14 - Fig 6: Why not put an inset on the RB-TnSeq data as in Fig 2 that describes the color highlighting in addition to the caption? Keeping consistency between figures throughout would help the reader settle into some of the common patterns. Adding a cofitness statistic to the panels would be helpful. For Fig 6C, is there a hom analog on the plot?

24. 15 - "a mutant library of Rhodanobacter sp. FW510-T8" I'm not clear on the rationale for choosing this strain particularly.

25. 22 - "Just four steps account for 65% of the remaining gaps:" might be clearer as "the 143 remaining gaps" or "the gaps remaining from the latest revision of GapMind"

26. 22 - "Even for the Pseudomonadota, ... (the average goes from 1.27 to 1.26)." I found this section a bit confusing while reading, although I think I grasp the idea.

27. 23 - Fig 8: Why not add the exact numbers 3, 4 and 5? I don't think the figure would be too crowded.

28. 24 - "For metB, GapMind identified two medium-confidence candidates, both of which have greater similarity to methionine gamma-lyases (73-77% identity) than to known MetB proteins (44-48%)." Is there an automated step involved in GapMind which could perform this kind of check?

29. 29 - "We moved the pKTO49_NN1 plasmid library into the E. coli conjugation donor WM3064 via electroporation to make the conjugation donor strain AMD1385." If available, estimating the library size at this point (based on number of transformants) could be useful for others.

30. 30 - Adding the closure type for tubes, the bottom geometry of well plates (2 mL deep well?), and the stroke length of the shaker would be helpful.

31. 35 - "Although this homology is remote" I would prefer "sequence similarity" here myself, I try to treat "homology" as binary.

Comments:

32. 22 - "We speculate that other kinases, phosphatases, or pyrophosphatases have evolved to act on these substrates and sometimes replace the known families. In any case, the absence of these genes should not be used to predict auxotrophies." I wonder whether GapMind already does, or could, include an automated recommendation when the only gaps remaining in a pathway are phosphotransfer-related, to this effect.

Copy editing:

33. 4 - "So GlyXL may use a different mechanism." "so" > "for this reason" or "accordingly"?

34. 6 - "(But they are not encoded nearby in M. maripaludis.)" "but" > "however"?

35. 13 - "In most Gammaprotebacteria," missing o in Gammaproteobacteria

36. 16 - "chloramphenicol to retain the plasmid.." extra .

37. 29 - "10 ug/mL kanamycin," here and elsewhere, should be μg/mL

38. 29 - "was recovered from the freezer in the rich media that used to make the library," add "was"

**********

**Have all data underlying the figures and results presented in the manuscript been provided?**

Large-scale datasets should be made available via a public repository as described in the *PLOS Genetics*  data availability policy, and numerical data that underlies graphs or summary statistics should be provided in spreadsheet form as supporting information.

Reviewer #1: **No:**  It would be helpful to provide more data behind the figures and information in the text (see reviewer comment 7), such as information on gene presence/absence in the genomes, or transposon insertion mutant library results from which the figures are made

Reviewer #2: Yes

**********

PLOS authors have the option to publish the peer review history of their article (what does this mean?). If published, this will include your full peer review and any attached files.

If you choose “no”, your identity will remain anonymous but your review may still be made public.

**Do you want your identity to be public for this peer review?**  For information about this choice, including consent withdrawal, please see our Privacy Policy.

Reviewer #1: No

Reviewer #2: **Yes:**  B Thuronyi

[NOTE: If reviewer comments were submitted as an attachment file, they will be attached to this email and accessible via the submission site. Please log into your account, locate the manuscript record, and check for the action link "View Attachments". If this link does not appear, there are no attachment files.]

**Figure resubmission:**

**Reproducibility:**

To enhance the reproducibility of your results, we recommend that authors deposit laboratory protocols in protocols.io, where a protocol can be assigned its own identifier (DOI) such that it can be cited independently in the future. Additionally, PLOS ONE offers an option to publish peer-reviewed clinical study protocols. Read more information on sharing protocols at https://plos.org/protocols?utm_medium=editorial-email&utm_source=authorletters&utm_campaign=protocols

# Author comment: Author response to Decision Letter 1

**DOI:** 10.1371/journal.pgen.1012372.r002

23 Oct 2026

Attachment Submitted filename: responses.pdf

# Editor report: Decision Letter 1

**DOI:** 10.1371/journal.pgen.1012372.r003

**Authors:** Sean Crosson, Ankur B. Dalia

27 Oct 2026

Dear Dr Price,

We are pleased to inform you that your manuscript entitled "Novel enzymes for amino acid biosynthesis in diverse bacteria and archaea: GapMind 2026" has been editorially accepted for publication in PLOS Genetics. Congratulations!

Before your submission can be formally accepted and sent to production you will need to complete our formatting changes, which you will receive in a follow up email. Please be aware that it may take several days for you to receive this email; during this time no action is required by you. Please note: the accept date on your published article will reflect the date of this provisional acceptance, but your manuscript will not be scheduled for publication until the required changes have been made.

Once your paper is formally accepted, an uncorrected proof of your manuscript will be published online ahead of the final version, unless you’ve already opted out via the online submission form. If, for any reason, you do not want an earlier version of your manuscript published online or are unsure if you have already indicated as such, please let the journal staff know immediately at plosgenetics@plos.org.

In the meantime, please log into Editorial Manager at https://www.editorialmanager.com/pgenetics/, click the "Update My Information" link at the top of the page, and update your user information to ensure an efficient production and billing process. Note that PLOS requires an ORCID iD for all corresponding authors. Therefore, please ensure that you have an ORCID iD and that it is validated in Editorial Manager. To do this, go to ‘Update my Information’ (in the upper left-hand corner of the main menu), and click on the Fetch/Validate link next to the ORCID field.  This will take you to the ORCID site and allow you to create a new iD or authenticate a pre-existing iD in Editorial Manager.

If you have a press-related query, or would like to know about making your underlying data available (as you will be aware, this is required for publication), please see the end of this email. If your institution or institutions have a press office, please notify them about your upcoming article at this point, to enable them to help maximise its impact. Inform journal staff as soon as possible if you are preparing a press release for your article and need a publication date.

Thank you again for supporting open-access publishing; we are looking forward to publishing your work in PLOS Genetics!

Yours sincerely,

Ankur B. Dalia

Academic Editor

PLOS Genetics

Sean Crosson

Section Editor

PLOS Genetics

Aimée Dudley

Editor-in-Chief

PLOS Genetics

Anne Goriely

Editor-in-Chief

PLOS Genetics

www.plosgenetics.org

BlueSky: @plos.bsky.social

----------------------------------------------------

Comments from the reviewers (if applicable):

----------------------------------------------------

**Data Deposition**

If you have submitted a Research Article or Front Matter that has associated data that are not suitable for deposition in a subject-specific public repository (such as GenBank or ArrayExpress), one way to make that data available is to deposit it in the Dryad Digital Repository. As you may recall, we ask all authors to agree to make data available; this is one way to achieve that. A full list of recommended repositories can be found on our website.

The following link will take you to the Dryad record for your article, so you won't have to re‐enter its bibliographic information, and can upload your files directly:

http://datadryad.org/submit?journalID=pgenetics&manu=PGENETICS-D-26-00751R1

More information about depositing data in Dryad is available at http://www.datadryad.org/depositing. If you experience any difficulties in submitting your data, please contact help@datadryad.org for support.

Additionally, please be aware that our data availability policy requires that all numerical data underlying display items are included with the submission, and you will need to provide this before we can formally accept your manuscript, if not already present.

----------------------------------------------------

**Press Queries**

If you or your institution will be preparing press materials for this manuscript, or if you need to know your paper's publication date for media purposes, please inform the journal staff as soon as possible so that your submission can be scheduled accordingly. Your manuscript will remain under a strict press embargo until the publication date and time. This means an early version of your manuscript will not be published ahead of your final version. PLOS Genetics may also choose to issue a press release for your article. If there's anything the journal should know or you'd like more information, please get in touch via plosgenetics@plos.org.

# Editor report: Acceptance letter

**DOI:** 10.1371/journal.pgen.1012372.r004

**Authors:** Sean Crosson, Ankur B. Dalia

PGENETICS-D-26-00751R1

Novel enzymes for amino acid biosynthesis in diverse bacteria and archaea: GapMind 2026

Dear Dr Price,

We are pleased to inform you that your manuscript entitled "Novel enzymes for amino acid biosynthesis in diverse bacteria and archaea: GapMind 2026" has been formally accepted for publication in PLOS Genetics! Your manuscript is now with our production department and you will be notified of the publication date in due course.

The corresponding author will soon be receiving a typeset proof for review, to ensure errors have not been introduced during production. Please review the PDF proof of your manuscript carefully, as this is the last chance to correct any errors. Please note that major changes, or those which affect the scientific understanding of the work, will likely cause delays to the publication date of your manuscript.

Soon after your final files are uploaded, unless you have opted out or your manuscript is a front-matter piece, the early version of your manuscript will be published online. The date of the early version will be your article's publication date. The final article will be published to the same URL, and all versions of the paper will be accessible to readers.

For Research Articles, you will receive an invoice from PLOS for your publication fee after your manuscript has reached the completed accept phase. If you receive an email requesting payment before acceptance or for any other service, this may be a phishing scheme. Learn how to identify phishing emails and protect your accounts at https://explore.plos.org/phishing.

Thank you again for supporting PLOS Genetics and open-access publishing. We are looking forward to publishing your work!

With kind regards,

Anitha Samidurai

PLOS Genetics

On behalf of:

The PLOS Genetics Team

Carlyle House, Carlyle Road, Cambridge CB4 3DN | United Kingdom

plosgenetics@plos.org | +44 (0) 1223-442823

plosgenetics.org | Twitter: @PLOSGenetics
